# The genome of the stable fly, *Stomoxys calcitrans*, reveals potential mechanisms underlying reproduction, host interactions, and novel targets for pest control

**DOI:** 10.1186/s12915-021-00975-9

**Published:** 2021-03-10

**Authors:** Pia U. Olafson, Serap Aksoy, Geoffrey M. Attardo, Greta Buckmeier, Xiaoting Chen, Craig J. Coates, Megan Davis, Justin Dykema, Scott J. Emrich, Markus Friedrich, Christopher J. Holmes, Panagiotis Ioannidis, Evan N. Jansen, Emily C. Jennings, Daniel Lawson, Ellen O. Martinson, Gareth L. Maslen, Richard P. Meisel, Terence D. Murphy, Dana Nayduch, David R. Nelson, Kennan J. Oyen, Tyler J. Raszick, José M. C. Ribeiro, Hugh M. Robertson, Andrew J. Rosendale, Timothy B. Sackton, Perot Saelao, Sonja L. Swiger, Sing-Hoi Sze, Aaron M. Tarone, David B. Taylor, Wesley C. Warren, Robert M. Waterhouse, Matthew T. Weirauch, John H. Werren, Richard K. Wilson, Evgeny M. Zdobnov, Joshua B. Benoit

**Affiliations:** 1 Livestock Arthropod Pests Research Unit, USDA-ARS, Kerrville, TX USA; 2 Department of Epidemiology of Microbial Diseases, Yale School of Public Health, New Haven, CT USA; 3 Department of Entomology and Nematology, University of California – Davis, Davis, CA USA; 4 The Center for Autoimmune Genomics and Etiology, Cincinnati Children’s Hospital Medical Center, Cincinnati, OH USA; 5 Department of Entomology, Texas A & M University, College Station, TX USA; 6 Department of Biological Sciences, Wayne State University, Detroit, MI USA; 7 Department of Electrical Engineering & Computer Science, University of Tennessee, Knoxville, TN USA; 8 Department of Biological Sciences, University of Cincinnati, Cincinnati, OH USA; 9 Department of Genetic Medicine and Development, University of Geneva Medical School and Swiss Institute of Bioinformatics, 1211 Geneva, Switzerland; 10 The European Molecular Biology Laboratory, The European Bioinformatics Institute, The Wellcome Genome Campus, Hinxton, CB10 1SD UK; 11 Department of Entomology, University of Georgia, Athens, GA USA; 12 Department of Biology and Biochemistry, University of Houston, Houston, TX USA; 13 National Center for Biotechnology Information, National Library of Medicine, National Institutes of Health, Bethesda, MD USA; 14 Arthropod-borne Animal Diseases Research Unit, USDA-ARS, Manhattan, KS USA; 15 Department of Microbiology, Immunology and Biochemistry, University of Tennessee Health Science Center, Memphis, TN USA; 16 Section of Vector Biology, Laboratory of Malaria and Vector Research, National Institute of Allergy and Infectious Diseases, Rockville, MD USA; 17 Department of Entomology, University of Illinois at Urbana-Champaign, Urbana, IL USA; 18 Department of Biology, Mount St. Joseph University, Cincinnati, OH USA; 19 Informatics Group, Faculty of Arts and Sciences, Harvard University, Cambridge, MA USA; 20 Department of Entomology, Texas A&M AgriLife Research and Extension Center, Stephenville, TX USA; 21 Department of Computer Science & Engineering, Department of Biochemistry & Biophysics, Texas A & M University, College Station, TX USA; 22 Agroecosystem Management Research Unit, USDA-ARS, Lincoln, NE USA; 23 University of Missouri, Bond Life Sciences Center, Columbia, MO USA; 24 Department of Ecology and Evolution, University of Lausanne, and Swiss Institute of Bioinformatics, 1015 Lausanne, Switzerland; 25 Center for Autoimmune Genomics and Etiology, Divisions of Biomedical Informatics and Developmental Biology, Cincinnati Children’s Hospital Medical Center, Cincinnati, OH USA; 26 Department of Pediatrics, University of Cincinnati College of Medicine, Cincinnati, OH USA; 27 Department of Biology, University of Rochester, Rochester, NY USA; 28 Institute for Genomic Medicine, Nationwide Children’s Hospital, Columbus, OH USA; 29 College of Medicine, Ohio State University, Columbus, OH USA

**Keywords:** Stable fly genome, Muscid genomics, Insect orthology, Chemoreceptor genes, Opsin gene duplication, Metabolic detoxification genes, Insect adaptation, Gene regulation, Insect immunity

## Abstract

**Background:**

The stable fly, *Stomoxys calcitrans*, is a major blood-feeding pest of livestock that has near worldwide distribution, causing an annual cost of over $2 billion for control and product loss in the USA alone. Control of these flies has been limited to increased sanitary management practices and insecticide application for suppressing larval stages. Few genetic and molecular resources are available to help in developing novel methods for controlling stable flies.

**Results:**

This study examines stable fly biology by utilizing a combination of high-quality genome sequencing and RNA-Seq analyses targeting multiple developmental stages and tissues. In conjunction, 1600 genes were manually curated to characterize genetic features related to stable fly reproduction, vector host interactions, host-microbe dynamics, and putative targets for control. Most notable was characterization of genes associated with reproduction and identification of expanded gene families with functional associations to vision, chemosensation, immunity, and metabolic detoxification pathways.

**Conclusions:**

The combined sequencing, assembly, and curation of the male stable fly genome followed by RNA-Seq and downstream analyses provide insights necessary to understand the biology of this important pest. These resources and new data will provide the groundwork for expanding the tools available to control stable fly infestations. The close relationship of *Stomoxys* to other blood-feeding (horn flies and *Glossina*) and non-blood-feeding flies (house flies, medflies, *Drosophila*) will facilitate understanding of the evolutionary processes associated with development of blood feeding among the Cyclorrhapha.

**Supplementary Information:**

The online version contains supplementary material available at 10.1186/s12915-021-00975-9.

## Background

Livestock ectoparasites are detrimental to cattle industries in the USA and worldwide, impacting both confined and rangeland operations. Flies from the Muscidae family commonly occupy these settings, including the nonbiting house fly and face fly and the blood-feeding (hematophagous) stable fly and horn fly. These muscid flies exhibit different larval and adult biologies, including diversity in larval developmental substrates, adult nutrient sources, and feeding frequency [1, 2]. As such, control efforts against these flies are not one size fits all. The stable fly, *Stomoxys calcitrans* (L.), in particular, is a serious hematophagous pest with a cosmopolitan host range, feeding on bovids, equids, cervids, canines, and occasionally humans throughout much of the world [2]. The stable fly’s painful bites disrupt livestock feeding behavior [3–6]; these bites can be numerous during heavy infestation, leading to reductions of productivity by over $2 billion USD [7].

Stable fly larvae occupy and develop in almost any type of decomposing vegetative materials that are often contaminated with animal wastes [8]. In Australia, Brazil, and Costa Rica, dramatic increases in stable fly populations have coincided with the expansion of agricultural production where the vast accumulation of post-harvest byproducts are recognized as nutrient sources for development of immature stages [9–11]. The active microbial communities residing in these developmental substrates (e.g., spent hay, grass clippings, residues from commercial plant processing, manure) are required for larval development and likely provide essential nutrients [12]. Even though stable flies are consistently exposed to microbes during feeding and grooming activities, biological transmission (uptake, development, and subsequent transmission of a microbial agent by a vector) of pathogens has not been demonstrated for organisms other than the helminth *Habronema microstoma* [13, 14]. Stable flies have been implicated in mechanical (non-biologic) transmission of Equine infectious anemia, African swine fever, West Nile, and Rift Valley viruses, *Trypanosoma* spp., and *Besnoitia* spp. (reviewed by [13]). The apparent low vector competence of stable flies implicates the importance of immune system pathways not only in regulating larval survival in microbe-rich environments but also in the inability of pathogens to survive and replicate in the adult midgut following ingestion [15–17].

Stable flies rely on chemosensory input to localize nutritional resources, such as volatiles emitted by cattle [18–23] and volatiles/tastants produced from plant products [24–26]. Stable fly mate location and recognition are largely dependent upon visual cues and contact pheromones [27, 28], and gravid females identify suitable oviposition sites through a combination of olfactory and contact chemostimuli along with physical cues [12, 19, 20, 23, 29]. Since stable flies infrequently associate with their hosts, feeding only 1 to 2 times per day, on-animal and pesticide applications are less effective control efforts than those that integrate sanitation practices with fly population suppression by way of traps [30]. Given the importance of chemosensory and vision pathways, repellents have been identified that target stable fly chemosensory inputs and current trap technologies exploit stable fly visual attraction [31–33]. However, despite these efforts, consistent control of stable fly populations remains challenging and development of novel control mechanisms is greatly needed.

Although both sexes feed on sugar, adults are reliant on a bloodmeal for yolk deposition and egg development, as well as seminal fluid production [26, 34]. Blood feeding evolved independently on at least five occasions within the Diptera, in the Culicimorpha, Psychodomorpha, Tabanomorpha, Muscoidea, and Hippoboscoidea [35]. The Muscinae appear to have a high propensity for developing blood feeding; which has occurred at least four times within this subfamily—once in each of the *domestica*-, *sorbens*- and *lusoria*- groups and again in the Stomoxini [36]. Unlike other groups of blood-feeding Diptera where non-blood feeding ancestors are distantly related and / or difficult to discern, stomoxynes are imbedded with the subfamily Muscinae of the Muscidae, featuring many non-blood feeding species. Contrasting blood-feeding culicimorphs and tabanimorphs, stable flies exhibit gonotrophic discordance [37, 38], requiring three to four blood meals for females to develop their first clutch of eggs and an additional two to three for each subsequent clutch of eggs. These unique aspects of stable flies offer opportunities for comparative analysis of the genomic features underlying these key biological traits.

Even with the importance of the stable fly as a pest, little is known about the molecular mechanisms underlying the biology of *S. calcitrans*. To further our understanding of this critical livestock pest, we report a draft genome sequence of the stable fly. The quality of this genome is high and includes in silico annotation that was aided by extensive developmental and tissue-specific RNA-Seq data focusing on the feeding and reproduction of *S. calcitrans*. Manual curation and comparative analyses focused on aspects underlying the role of this fly as a pest related to host interactions, reproduction, control, and regulation of specific biological processes. Our study significantly advances the understanding of stable fly biology including the identification of unique molecular and physiological processes associated with this blood-feeding fly. These processes can serve as novel targets which will assist in both developing and improving control of this important livestock pest.

## Results and discussion

### Genome assembly and annotation supported by comparative and functional genomics

Whole genome shotgun sequencing of pooled, teneral adult males from an *S. calcitrans* line developed for this project resulted in the 66x coverage draft assembly of 971 MB of total sequence (see Additional file 1: Table S1). Scaffolds (12,042) and contigs (125,702) had N50 lengths of 504.7 and 11.3 kb, respectively. The sequence was ~ 20% smaller compared to the genome predicted by propidium iodide analyses (~ 1150 MB, [39]). This difference is likely the result of heterochromatin and other repetitive regions that were unassembled, as genome size is not significantly different between the sexes [39] and is comparable to differences documented for other insect genomes [40–42]. There were 16,102 predicted genes/pseudogenes that included 2003 non-protein coding genes, and a total of 22,450 mRNA transcripts were predicted with over 94% supported by RNA-Seq (see Additional file 2). The *S. calcitrans* mitochondrial genome has been previously described [43]. Its 18 kb genome encodes 13 protein-coding, 2 rRNA and 23 tRNA gene sequences. In addition, sequence analysis revealed an extra copy of the *trnI* gene, similar to a blow fly duplication, and partial sequences of the control region. Manual curation and analyses of the nuclear genome sequences allowed preliminary chromosome arm assignment and identification of repeat elements from the genome (see Additional file 1: Tables S2 and S3 [44–61];). A select set of protein coding genes (*n* = 1600) were manually annotated to identify genes associated with functional classes including immunity, host sensing, reproduction, feeding, and metabolic detoxification.

**Fig. 1:**
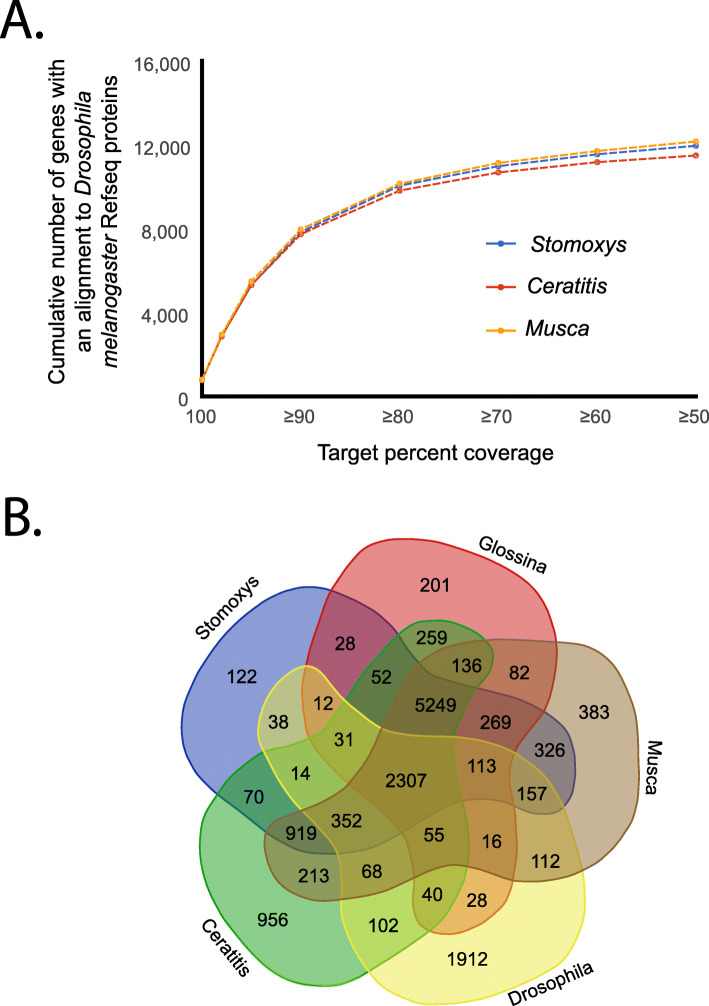
Quality assessment of the *Stomoxys calcitrans* genome. **a** Number of genes with alignment to *Drosophila melanogaster* genome. **b** Ortholog group comparison between *S. calcitrans* (*Stomoxys*), *Musca domestica* (*Musca*), *Ceratitis capitata* (*Ceratitis*), *Glossina morsitans* (*Glossina*), and *D. melanogaster* (*Drosophila*) based on comparison to the OrthoDB8 database

Completeness of the genome assembly was assessed via comparison of the *S. calcitrans* genome and predicted gene set against a database of fly derived benchmarking universal single-copy ortholog genes (BUSCOs [62]). Based on the near-universal single-copy orthologs from dipterans (OrthoDB v8, [63]), 95.1% were found in the assembly and 92.2% in the final predicted gene set (see Additional file 1: Figure S1 [63–67];).Comparison of the *Stomoxys* gene set against that of *Drosophila melanogaster* revealed approx. 10,000 *Stomoxys* genes with at least 75% target coverage, which is a similar number of genes with significant alignments relative to that of other published fly genomes (Fig. 1). Lastly, CEGMA genes and those associated with autophagy were all identified from the *S. calcitrans* genome (see Additional file 1: Table S4 and Additional file 3). These gene set comparisons provide an additional metric of genome completeness as these are highly conserved among flies [40, 68]. These metrics indicate that the genome is of sufficient quality for subsequent comparative analyses with other insects, specifically in relation to gene content. Comparison of protein orthologs revealed 122 *Stomoxys* species-specific protein families relative to other higher flies (Fig. 1). Based on gene ontology, there was enrichment for zinc finger transcription factors in relation to all genes (*p* = 0.02–0.007; see Additional file 2), which has also been documented in other insect systems [69, 70].

Evaluation of gene expression by RNA-Seq produced a comprehensive catalog of sex-biased gene expression as well as genes enriched in different developmental stages and organs (see Additional file 4). Samples of RNA were sequenced from the following conditions: whole females (teneral and mated, reproductive), whole males (teneral), male reproductive tracts (mated), female reproductive tracts (mated), male heads (fed, mated), female heads (fed, mated), a third instar larva, and female/male salivary glands. Each condition consisted of a single replicate. These datasets will serve as a valuable resource for future studies. A stringent statistical significance cutoff (FDR, *P* < 0.01) was used as only a single replicate was analyzed for each treatment [40, 69, 70]. A significant correlation (Pearson’s *R*^2^ = 0.8643, *P* < 0.0001; Figure S2) was observed between log_2_ fold changes of reverse transcription quantitative real-time PCR (RT-qPCR) versus normalized expression values of 25 genes (see Additional file 1; Figure S2, Table S5), validating that we can glean biological relevance from the RNA-Seq datasets.

### Limited evidence for lateral gene transfer and no evidence of endosymbionts in the *Stomoxys* genome of a laboratory-colonized strain

Stable fly larvae are absolutely dependent on bacteria for survival and development [12, 29, 71], and larval physiology impacts the composition of this microbial community irrespective of the substrate in which the larvae are developing [17]. As such, use of bacterial communities to deliver targeted control technologies, e.g., gene silencing constructs, is an approach being considered for use with stable flies [72], which led us to analyze culturable bacteria from surface-sterilized, adult *S. calcitrans* collected from Texas dairies. This revealed a variety of genera representing the adult gut bacterial community (see Additional file 5). Among those cultured, the most abundant phyla represented were Proteobacteria and Firmicutes with only 3% of isolates classified as Actinobacteria and Bacteroidetes. Similar to mosquitoes, harbored bacterial communities were likely ingested while grooming or acquired during ingestion of nectar or water sources, as strict blood feeders usually have reduced gut microbiota [73–75]. As such, the most prevalent genus was *Aeromonas* sp., which are frequently found in aquatic and wet environments, such as irrigation water, fecal matter, and moist organic waste, and have been cultured from other arthropods [76]. In addition, the adult *S. calcitrans* bacterial communities share similarity with those isolated from other flies associated with filth and decomposition (e.g., blow flies and other related species, [77, 78]), suggesting these bacteria may also be acquired by *S. calcitrans* adults when visiting oviposition sites.

Given this association with microbial communities, *Stomoxys* may contain laterally transferred genes for bacteria that impact on their biology, as in other reported insect genomes [79]. In addition to identifying potential symbiotic associations revealed in the genome assembly, it is also important to rule out bacterial contaminating scaffolds that could mistakenly be attributed as components of the *Stomoxys* genome. For these reasons, a DNA based pipeline was used to screen for potential contaminating bacteria scaffolds and possible lateral gene transfers (LGTs) from bacteria into the *Stomoxys* genome. The pipeline revealed only trace bacterial contaminants in the genome assembly (see Additional file 1: Table S6). This contrasts with some other arthropod genome assemblies, which contained complete or nearly complete bacterial genomes, such as the parasitoid *Trichogramma pretiosum* [80], amphipod *Hyalella azteca* [81], and the bedbug *Cimex lectularius* [40]. The results suggest that a maternally inherited and/or environmentally acquired microbiota does not occur in any abundance in teneral *Stomoxys* males. Whether circumstances are different in adult females would require further study.

The LGT analysis revealed presence of three candidates (see Additional file 1: *Stomoxys* lateral gene transfer, Table S7), all of which were derived from *Wolbachia*, a common endosymbiont found in arthropods [82] that infects 40–60% of insect species [83, 84]. *Wolbachia* are a common source of LGTs [85, 86], likely due to their association with the germline of their insect hosts. The three candidates were examined for sequencing read depth in the candidate LGT and flanking DNA to determine if there were large changes in read depth across the junction that would be indicative of miss-assembly to contaminating bacterial DNA (see Additional file 1: Figure S3). The results did not reveal large changes in read depth across the junctions. Further, there is no evidence that any of the three LGTs contain functional protein coding genes, and only one had detectable expression in our RNA-Seq datasets that occurred within the 3′ UTR of XM_013245585 (see Additional file 1: Table S7). This gene encodes a transcription factor containing a basic leucine zipper domain*,* but whether expression of the LGT is a consequence of its location in the UTR or is biologically significant is unknown. While *Wolbachia* was described from the closely related horn fly, *Haematobia irritans* [87], the *Stomoxys* strain used for the genome sequencing was not infected with *Wolbachia*, no *Wolbachia* scaffolds were found in the assembly, and there are no reports of natural occurrence of *Wolbachia* in *Stomoxys* populations [88]. Presence of these LGTs, then, likely reflects LGT events from a past *Wolbachia* infection in the ancestors of *Stomoxys*. Stable flies will consume blood and nectar for nourishment [24, 26, 89], and this is different from the closely-related tsetse flies, which are obligate blood feeders. Due to this limited food source, tsetse flies (*Glossina morsitans*) harbor an obligate symbiont, *Wigglesworthia glossinidae*, that provides B vitamins that are present at low levels in blood [41, 90, 91]. Analysis of the assembled *S. calcitrans* genome, described above, did not reveal a distinct microbial symbiont.

### The *Stomoxys* immune system encodes gene family expansions that may reflect adaptation to larval development in microbe-rich substrates

The closely related house fly, *Musca domestica*, occupies microbe-rich environments that overlap with those of the stable fly, particularly in livestock production settings. Noted expansions in immune system-related gene families of the house fly are hypothesized to be a consequence of this ecology [92–94]. Analysis of the *S. calcitrans* genome revealed extensive conservation of immune system signaling pathways coupled with dramatic expansions of some gene families involved in both recognition and effector functions. The insect immune system—best characterized from work in the model organism *D. melanogaster*—includes both cellular defenses (e.g., macrophage-like cells that phagocytose pathogenic microorganisms) and a humoral defense system that results in the production of antimicrobial effector molecules [95]. The humoral immune system can be divided into recognition proteins, which detect pathogenic bacteria and fungi; signaling pathways, which are activated by recognition proteins and result in the translocation of transcription factors to the nucleus to induce gene expression; and effectors, which are (typically) secreted and ultimately act to clear infections.

Previous comparative work suggests that at least some parts of the immune system are deeply conserved across Dipterans and indeed most insects. Genes encoding immune signaling proteins, in particular, are generally preserved as single-copy orthologs across a wide range of insects [41, 93, 94, 96–101], with only rare exceptions [102]. Despite the strong conservation of the basic structure of the main signaling pathways in insect immunity, there is considerable evidence for variation in both the gene content and protein sequence of the upstream inputs (recognition proteins) and downstream outputs (effector proteins) of the immune system (e.g., [93, 98–100, 103, 104]).

Major components of the Toll, Imd, JAK/STAT, p38, and JNK pathways in the *S. calcitrans* genome (see Additional file 1: Table S8) were found to be largely conserved as single-copy orthologs [95]. A description and full lists of putative computationally annotated and manually curated immune-related genes in *S. calcitrans* is provided (see Additional file 1: Table S9, [15, 105–112]; Additional files 6 and 7). These findings are consistent with previous reports for many other Dipterans and supports the conclusion that the intracellular signaling mechanisms of innate immunity have been stable during the evolutionary history of Dipterans [97, 98, 100]. In contrast, the gene families encoding upstream recognition proteins and downstream effector proteins tend to be expanded in *S. calcitrans* and *M. domestica* relative to other Dipterans (Table 1).

**Table 1:** Number of immune-related gene family members from sequenced Dipteran genomes, annotated by hidden Markov models

	***Scal***	***Mdom***	***Gmor***	***Aaeg***	***Dmel***
**Canonical pattern recognition**					
Nimorod (NIM)	25	23	10	8	17
Peptidoglycan recognition protein (PGRP)	17	17	4	10	13
beta-1,3-glucan-binding proteins (BGBP)	4	3	3	7	7
Thioester containing proteins (TEP)	16	22	4	8	6
**Other recognition**					
C-type lectin (CTL)	78	41	11	43	38
Fibrinogen-related proteins (FREP)	49	38	7	34	14
Galectins (GALE)	15	13	8	12	6
Immunoglobulin superfamily (IGSF)	1	1	1	0	1
MD2-like proteins (MD2L)	8	12	5	26	8
Scavenger receptor class A (SRCA)	3	3	2	2	3
Scavenger receptor class B (SRCB)	15	18	11	13	14
Scavenger receptor class C (SRCC)	7	8	4	5	9
**Canonical effectors**					
Attacin antimicrobial peptides (ATT)	11 (12)*	10	4	1	4
Defensin antimicrobial peptides (DEF)	5 (11)*	5	0	4	1
Diptericin antimicrobial peptides (DIPT)	3 (1)*	4	0	1	3
Cecropin antimicrobial peptides (CEC)	5 (10)*	12	2	9	5
Lysozymes (LYS)	23	32	4	7	13
**Non-canonical effectors**					
Thioredoxin peroxidases (TPX)	5	6	6	5	8
Prophenoloxidases (PPO)	19	23	4	25	10
Glutathione peroxidases (GPX)	1	1	0	3	2
Heme peroxidases (HPX)	12	12	8	19	10
Transferrins (TSF)	4	6	3	5	3

*Numbers in parentheses are those numbers annotated after manual curation of the *S. calcitrans* genome. *Scal: Stomoxys calcitrans*, *Mdom: Musca domestica*, *Gmor: Glossina morsitans*, *Aaeg: Aedes aegypti*, *Dmel: Drosophila melanogaster*

Hidden Markov Model profiles and manual curation were used to analyze four canonical recognition families with well-characterized immune roles and an additional eight families with less well-defined roles (Table 1). For three of the four canonical pattern recognition receptor families (NIM, PGRP, and TEP), and four of the other families (CTL, FREP, GALE, and SRCB), the *S. calcitrans* genome encodes either the most members or second-most members after *M. domestica* among the 5 Dipteran genomes screened (*S. calcitrans* plus *Aedes aegypti*, *D. melanogaster*, *M. domestica*, and *G. morsitans*). A similar pattern holds for downstream effector proteins: the *S. calcitrans* genome encodes either the most or second-most after *M. domestica* for attacins (ATT), defensins (DEF), cecropins (CEC), and lysozymes (LYS). In contrast, comparable numbers of non-canonical effector gene family members were identified across the Dipteran genomes screened. Not unexpectedly, three additional classes of antimicrobial peptides (AMP) were originally characterized in *D. melanogaster* but are missing from the *M. domestica* genome ([94]; Metchnikowin, Drosocin, Drosomycin) and are also not detected in the *S. calcitrans* genome.

Several of the expanded AMP gene families were found tandemly arranged on individual scaffolds (see Additional file 1: Table S9). For example, the 11 *Stomoxys defensin* genes are located on a single scaffold (KQ079966) in two clusters, one of which includes five *defensins* present upstream of the other that includes six *defensins* (see Additional file 1: Figure S4). Within the downstream cluster, three genes were detected by RNA-Seq exclusively in larva and have 84–97% amino acid identity to each other, while the other three share 81–87% amino acid identity and were detected in heads of fed adults and male reproductive tracts but not in teneral adults (see Additional file 1: Table S10). The sequence identity and shared expression pattern support the likelihood that these sub-groups arose separately from gene duplication events. In contrast, the five *defensin* genes in the upstream cluster share 30–66% amino acid identity, and four were detected in teneral adults with variable detection in tissues of fed adults. This expression pattern is consistent with that reported for *Stomoxys midgut defensin 1* (*Smd1*) and *Smd2*, both of which are present in this upstream cluster [109].

In combination with the previously reported expansions of many effector and recognition immune components in the house fly [93, 94], our analysis of the *S. calcitrans* genome suggests that Muscidae likely have expanded the diversity of their immune repertoires, sometimes dramatically, despite differences in adult feeding ecology (blood feeder vs generalist). Muscid flies complete their life cycle in animal waste and other septic environments that are rich in communities of pathogenic and non-pathogenic bacteria. These communities and their metabolic products serve as the essential nutrient source for larval development, and larvae are repeatedly interacting with these bacteria. Further, the communities are in constant flux within the substrate over the course of this development. One hypothesis is that the shared diversification of immune receptors and effectors is driven by this larval ecology, while additional *M. domestica* specific expansions (e.g., in TEPs) are accounted for by the saprophytic adult feeding behavior of that species.

### Gustatory and ionotropic receptor gene family expansions support importance of bitter taste perception in *Stomoxys*

**Fig. 2:**
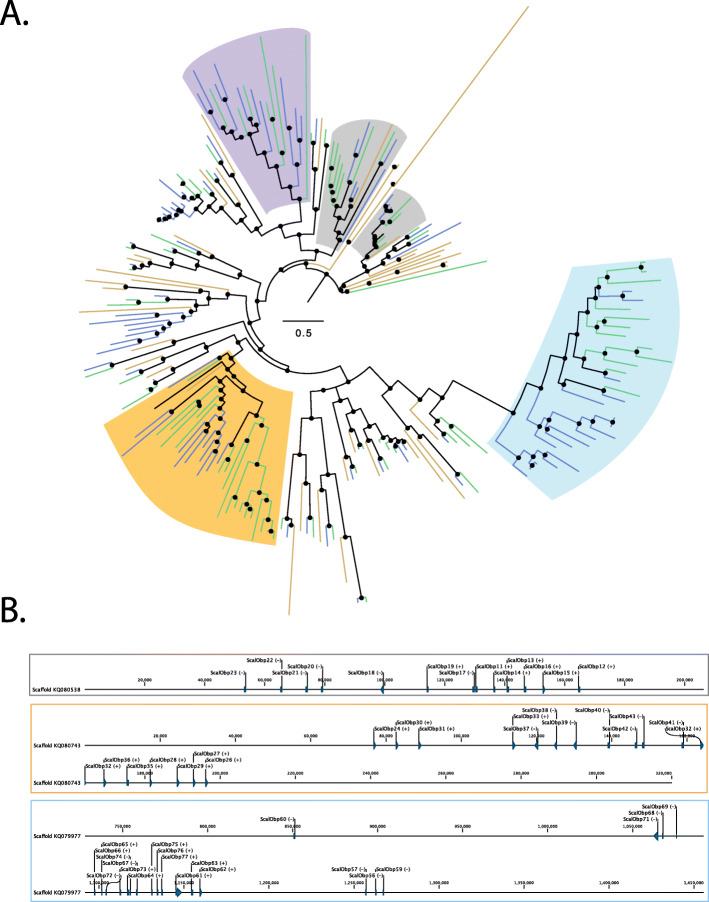
Phylogenetic tree of the *Stomoxys calcitrans* OBPs with those of *Drosophila melanogaster* and *Musca domestica* and locations of OBPs on *Stomoxys* scaffolds. **a** Maximum likelihood phylogeny was constructed using the web server version of IQ-TREE software ([114]; best-fit substitution model, branch support assessed with 1000 replicates of UFBoot bootstrap approximation). The full phylogenetic tree can be found in Additional file 1: Figure S8. The *S. calcitrans* and *M. domestica* lineages are in green and blue, respectively, while *D. melanogaster* lineages are in mustard. Clades that are expanded in the muscids relative to *Drosophila* or lost in *Drosophila* are shaded in orange and gray. Purple shading comprises a clade of muscid OBPs with no apparent ortholog in *Drosophila*, while blue shading comprises a clade that includes the *Drosophila* Minus-C OBP subfamily and represents an apparent muscid expansion relative to *DmelObp99c*. **b** Three *Stomoxys* scaffolds on which 51 of 90 OBPs are organized. The location of each OBP gene is indicated by a horizontal line. Transcriptional directions are indicated by (+) for same direction as the scaffold or (−) for the opposite direction. The color of the box reflects the shaded clades in **a**

**Fig. 3:**
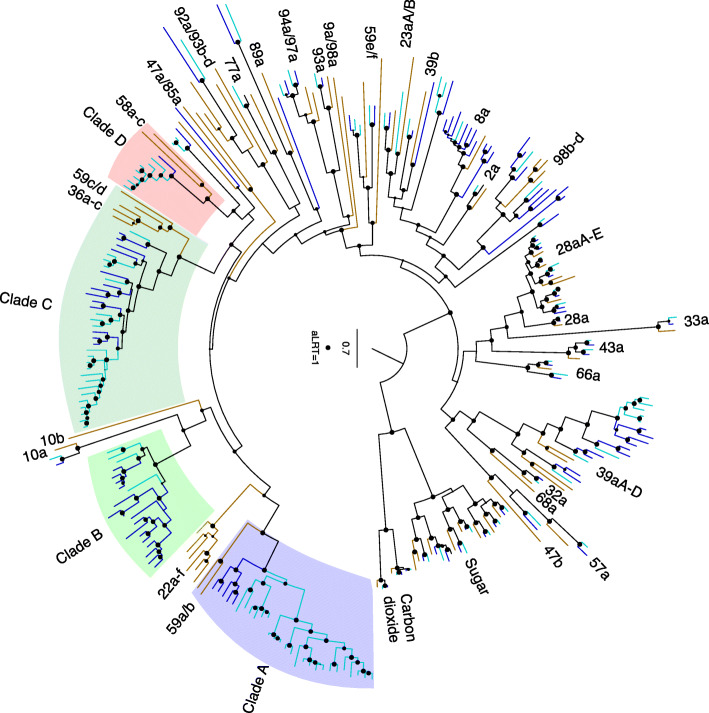
Phylogenetic tree of the *Stomoxys calcitrans* GRs with those of *Drosophila melanogaster* and *Musca domestica. Maximum* likelihood tree rooted by divergent carbon dioxide and sugar receptor subfamilies as the outgroup. The *S. calcitrans* and *M. domestica* lineages are highlighted in teal and blue, respectively, while *D. melanogaster* lineages are in mustard. Support levels from the approximate likelihood-ratio test (aLRT) from the PhyML v3.0 web server are shown. Subfamilies and individual or clustered *Drosophila* genes are indicated outside the circle to facilitate finding them in the tree. Four clades of candidate bitter receptors that are expanded in the muscids are highlighted. Scale bar indicates amino acid substitutions per site. The full phylogenetic tree can be found in Additional file 1: Figure S11

**Fig. 4:**
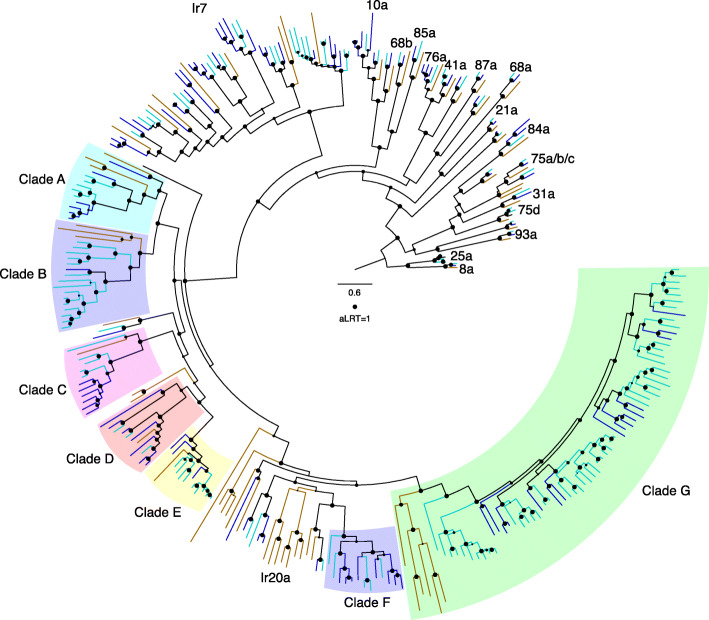
Phylogenetic tree of the *Stomoxys calcitrans* IRs with those of *Drosophila melanogaster* and *Musca domestica. Maximum* likelihood tree rooted with the Ir8a/25a lineage as the outgroup. The *S. calcitrans* and *M. domestica* lineages are in teal and blue, respectively, while *D. melanogaster* lineages are in mustard. Support levels from the approximate likelihood-ratio test from the PhyML v3.0 are shown. Subfamilies, clades, and individual *Drosophila* genes are indicated outside the circle to facilitate finding them in the tree. Pseudogenic sequences are indicated with the suffix P. Scale bar indicates amino acid substitutions per site. The full phylogenetic tree can be found in Additional file 1: Figure S12

Insect ecology and environment impact the size of chemosensory gene families with evidence for specialist insects having a smaller number of genes compared with generalists, with exceptions [113]. These gene families encode odorant binding proteins (OBP), carrier proteins for lipid molecules, as well as odorant (OR), gustatory (GR), and ionotropic (IR) receptors that display different affinities for ligands that mediate the insect’s response. *Glossina* are obligate blood-feeders and have a reduced number of chemoreceptors relative to more polyphagous insects that have been sequenced [41], while the *Musca* genome harbors an expanded number of chemoreceptors relative to *Drosophila* [94]. Analysis of the *S. calcitrans* genome revealed that it shares with *Musca* the presence of lineage-specific expansions and signatures of many pseudogenizations/deletions of chemosensory pathway genes relative to *Drosophila* (Figs. 2, 3 and 4, see Additional file 1: *Stomoxys* Chemosensory Gene Families [18–20, 23, 42, 94, 115–187] and Additional file 8), consistent with the birth-and-death model of evolution proposed for these gene families [188].

More than half of the 90 *S. calcitrans* OBP gene models are organized as tandem clusters across three scaffolds, consistent with OBP gene organization in other dipteran genomes [189] (Fig. 2, see Additional file 9 and Additional file 1: Figure S8). While this represents an increase in number of OBPs relative to *Drosophila*, it is comparable to the OBP gene family size in *M. domestica* [94], and it also corresponds with increases in gene family size for *S. calcitrans* chemoreceptor gene families described below. Expansions relative to and losses in *Drosophila* were evident in these clustered OBPs (Fig. 2). For example, an *S. calcitrans* cluster that resides on scaffold KQ079977 are in a lineage that includes the *Drosophila* Minus-C OBP subfamily and represent an apparent expansion of 17 *Stomoxys* and 16 *Musca* OBPs relative to *DmelObp99c*. Eighteen *Stomoxys* OBPs (ScalObp24-43) reside on scaffold KQ080743 and form a clade with nine Musca OBPs and *DmelObp56h*, depicting an expansion in this muscid lineage. Further, a separate clade comprised of seven *Stomoxys* OBPs (ScalObp79-85) and 11 *Musca* OBPs (MdomObp77-87) have no obvious *Drosophila* ortholog. Future studies to define functional roles for these OBPs are warranted, especially given the diverse tissues in which their expression was detected (see Additional file 1: Figure S8). Larval expression was detected by RNA-Seq for 35 *Obp*s, while 63 and 67 *Obp*s were detected in heads of mated adult males and females. However, 77 *Obps* were detected in the reproductive tract of mated adult, males and females (see Additional file 1: Figure S8), and this expression pattern supports non-chemosensory roles reported for OBPs in other dipteran species [190, 191]. For example, the transfer of OBPs from males to females in seminal fluid occurs in *Drosophila*, *Glossina*, and *Ae. aegypti* [192–194], and this may account for detection of *Obp* expression in tissues of *S. calcitrans* male and female reproductive tracts.

The OR and GR families make up the insect chemoreceptor superfamily [115, 117–119], with the OR family arising from a GR lineage at the base of the Insecta [113, 195]. Olfactory sensory neurons that detect volatile compounds express ORs, while GRs are mostly involved in taste, but some have an olfactory role [116]. The GR family is generally divided into three major and divergent subfamilies of sugar or sweet receptors, the carbon dioxide receptors, and the bitter taste receptors. A lineage within the bitter taste receptor clade has evolved into an important receptor for fructose [133], and there are others involved in courtship [123]. The ionotropic receptor family is a variant lineage of the ancient ionotropic glutamate receptor family [115, 138, 140, 169] and, like the GRs, they are involved in both olfaction and gustation, as well as sensing light, temperature, and humidity [169].

The carbon dioxide, sugar, and fructose receptors are relatively well conserved in *Stomoxys* and *Musca*, as is the case for many other insects. However, the bitter taste receptors reveal considerable gene family evolution both with respect to the available relatives of these muscid flies (*Drosophila* and the Mediterranean fruit fly, *Ceratitis capitata*) and between these two muscids. For example, a major expansion (*ScalGr29-57*) encoding 49 candidate bitter taste receptors occurs in *S. calcitrans*, comparable to a similarly complicated set in *M. domestica* (*MdomGr43-64*, encoding 35 proteins). Together, these form four major expanded clades in the muscids (Clades A–D, Fig. 3).

In *Drosophila* and most other insects examined to date, ionotropic receptors Ir8a and 25a, both of which function as co-receptors with other IRs [169], are highly conserved both in sequence and length and in being phylogenetically most closely related to the ionotropic glutamate receptors [138, 140]. While *Stomoxys* has the expected single conserved ortholog of Ir8a, surprisingly it has four paralogs of Ir25a (*ScalIr25a1-4*), the functions of which are enigmatic, as such duplications of Ir25a have seldom been observed in other insects (Fig. 4).

The *Ir7a-g* and *11a* genes in *Drosophila* are expressed in larval and adult gustatory organs [140], but ligands for these receptors are unknown. This subfamily is considerably expanded in both *S. calcitrans* and *M. domestica* and, given their complexities, they are not named for their *Drosophila* relatives. Rather, these are part of the numbered series from Ir101, in the case of *Stomoxys* to Ir121 and in *Musca* to Ir126 (Fig. 4). These IR gene family expansions strongly suggest an expanded gustatory capacity. A large clade of “divergent” IRs in *Drosophila* is involved in gustation and is known as the Ir20a subfamily of 33 proteins [165, 170, 171]. This clade of mostly intronless genes is considerably expanded in *M. domestica* to 53 members (*MdomIr127-179*), and even more so in *S. calcitrans* to 96 members (*ScalIr122-217*), labeled clades A-G in the tree (Fig. 4). Putative IR ligands in *Drosophila* are involved in sensing carbonation, amino acids, and specific carbohydrates [159, 162, 170].

Transcript detection by RNA-Seq provided some insight into those *Stomoxys* tissues expressing chemosensory receptor gene family members. Seventeen *Or* genes were enriched in the reproductive tract of mated males, and these may have a role in sperm activation, as proposed for *Anopheles gambiae* [196]. Interestingly, three *Or*s (*ScalOr22, 54,* and *55*) were highly enriched in the reproductive tract of mated females relative to all other tissues examined (see Additional file 1: Figure S10 and Additional file 9), suggesting these *Or*s may have a role in female reproduction, possibly perceiving male derived chemicals transferred during copulation. Eleven *Ors* were detected in third instar larvae, and pilot evaluation by non-quantitative RT-PCR detected an additional nine *Or*s expressed in first and second instar but not in third instar larvae (see Additional file 9). This suggests that stable flies differentially utilize odorant receptors throughout immature development. Expression of all 20 of these *Ors* was not exclusive to the larval stages, and the apparent absence of larval-specific receptors in the stable fly may be a result of exposure to related compounds during the immature and adult stages (e.g., host dung, detritus).

The expression of 35 *Gr* transcripts was detected by RNA-Seq in heads of mated females and males, and 27 *Gr*s were enriched in the reproductive tracts of mated males (see Additional file 1: Fig. S11 and Additional file 9). Evidence from *Drosophila* supports the expression of GRs in neurons that innervate testes and oviducts [146], suggesting that these *S. calcitrans* GRs may have a role in mediating reproductive system function. Twenty-three *Grs* were detected in larvae, all of which clustered as candidate bitter taste receptors, suggesting they may mediate larval bitter sensing. Determination of the ligand specificities of these muscid receptors is required to fully understand the ecological significance of the differential expansions and contractions of their bitter taste abilities.

Twenty-three *Ir*s were detected by RNA-Seq in heads of mated females and males of which two and seven were enriched in the female and male tissue, respectively. Interestingly, five *Ir*s were detected in the female reproductive tract while 56 *Ir*s were detected in the male reproductive tract with 46 highly enriched in this male tissue (see Additional file 1: Figure S12 and Additional file 9), suggesting a potential critical role in fly reproduction or reproductive behaviors. Given the striking expansions and expression pattern of genes within this family, functional studies in *S. calcitrans* are warranted. Further characterization of these chemosensory gene families will facilitate the design of behavior-based control technologies that can be employed as part of integrated pest management strategies.

### Expansion of the long wavelength-sensitive Rh1 opsin subfamily in *Stomoxys* with evidence of tuning for diversified wavelength sensitivities via substitution

**Fig. 5:**
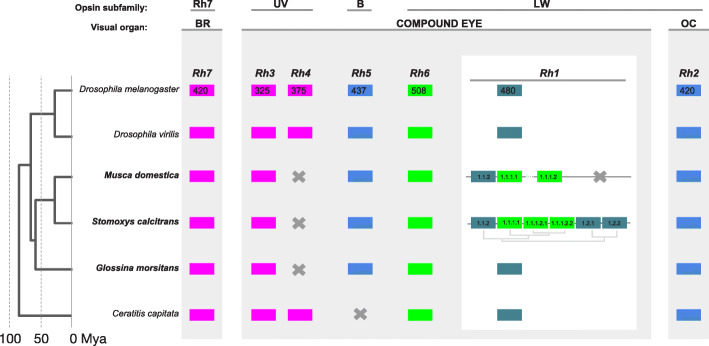
Opsin gene family members detected in *Stomoxys calcitrans* and other higher Diptera. A phylogenetic tree of dipteran opsin gene relationships is presented, as is the genomic organization and evolution of the *S. calcitrans* Rh1 opsin subfamily [200]. Protein sequences of *S. calcitrans* Rh1 genes were aligned with MUSCLE [201], and ambiguous alignment regions were filtered with Gblocks [202] using least stringent settings. Maximum likelihood tree was estimated in MEGA version 6.0 [203] applying the Jones-Taylor-Thornton (JTT) model of amino acid sequence evolution and assuming Gamma Distributed substitution rates across sites with 3 categories. Rhabdomeric opsins depicted: Rh7, Rh7 gene; UV, UV-sensitive; B, Blue-sensitive; LW, long wavelength-sensitive expressed in BR, compound eye, and OC, ocelli

Visual cues are integral to stable fly mating and host orientation [35], and stable fly attraction to particular wavelengths of light and UV reflectance has been manipulated for the development of trapping technologies to suppress populations [197]. As is typical for the generally fast flying calyptrate flies, stable fly adults of both sexes are equipped with large laterally positioned compound eyes in the head and three ocelli positioned in the dorsal head cuticle [198, 199]. Both achromatic motion tracking and color-specific perception tasks begin with the harvest of photons by members of the opsin class of G-protein coupled transmembrane receptors, which differ in their wavelength absorption optima. The genomic survey in the stable fly revealed conservation of most opsin gene subfamilies observed in *Drosophila* (Fig. 5; see Additional file 1: Figure S13 and Table S11). This included the UV sensitive opsin paralog *Rh3*, the blue sensitive opsin *Rh5*, several homologs of the long wavelength (LW) sensitive opsin *Rh1* and a 1:1 ortholog of LW opsin *Rh6*, all of which are expressed in subsets of photoreceptors in the compound eye retina [204]. In addition, we found 1:1 orthologs of the ocellus-specific opsin *Rh2* and of the recently characterized UV-sensitive, deep brain opsin *Rh7* [205] (Fig. 5; see Additional file 1: Figure S13 and Table S11). Overall, these findings are consistent with the electrophysiological and positive phototactic sensitivity of *S. calcitrans* to light in the UV, blue, and green range of visible light [33, 206].

*Drosophila* and other higher Diptera, including *C. capitata*, possess a second UV sensitive opsin gene *Rh4* (Fig. 5) [42, 207], which is not detected in the stable fly genome. The same result was previously obtained in the tsetse fly [41]. Global BLAST searches of calyptrate genomes were conducted (i.e. *G. morsitans, M. domestica*, the black blowfly (*Phormia regina*), and the flesh fly (*Sarcophaga bullata*)), and they failed to detect Rh4 orthologs. Thus, it can be concluded that the Rh4 opsin subfamily was lost during early calyptrate evolution.

#### An Rh1 opsin gene cluster in muscid Diptera

A unique aspect of the *S. calcitrans* opsin gene repertoire is the existence of six homologs of the LW opsin *Rh1* (Fig. 5; see Additional file 1: Figure S14, Table S11). Most higher Diptera sampled so far, including related species like the tsetse fly [41] and the black blowfly [208], possess a singleton Rh1 gene. Three Rh1 homologs, however, were detected in the *M. domestica* draft genome [94]. Moreover, in both *S. calcitrans* and *M. domestica*, these Rh1 homologs are closely linked and anchored as a cluster by homologous flanking genes (Fig. 5; see Additional file 1: Figure S14). This suggests that the Rh1 tandem gene clusters of the two species are homologous and date back to an ancestral cluster in the last common ancestor of muscid Calyptratae. Consistent with this, the *S. calcitrans* and *M. domestica* Rh1 homologs form a monophyletic unit in maximum likelihood trees estimated from amino acid or nucleotide sequence alignments of dipteran Rh1 homologs (see Additional file 1: Figure S14). Moreover, each of the three *Musca* Rh1 homologs grouped with strong support as 1:N orthologs with different members of the *Stomoxys* Rh1 gene cluster; however, two of the *Stomoxys* Rh1 genes (Rh1.2.1 and Rh1.2.2) lack *Musca* orthologs (see Additional file 1: Figure S14). Integrating the information on gene linkage, it is possible to conclude that the six Rh1 paralogs of *Stomoxys* originated by three early duplications before separating from the *Musca* lineage. While the latter subsequently lost one of the two earliest paralogs, the *Stomoxys* Rh1 cluster continued to expand by minimally one but possibly two subsequent tandem gene duplications (Fig. 5).

As expected, RNA-Seq results indicated that transcripts for all *Stomoxys* opsin genes, quantified by transcripts per million (TPM), were more abundant in the head relative to whole teneral adults of both sexes or male reproductive tracts (see Additional file 1: Table S11). The singleton opsin Rh1 from *Drosophila* is expressed in six outer photoreceptors (R1-6) that are found within each ommatidium. These receptors are specialized for motion detection. Opsins Rh5 and Rh6 are differentially expressed in a single color-vision specialized photoreceptor (R8) that are also within each ommatidium [204]. In the *Drosophila* modENCODE expression catalog, this is reflected as an up to 200 fold higher transcript abundance of Rh1 opsin compared to Rh5 or Rh6 in the adult heads of both sexes [209]. The *S. calcitrans* RNA-Seq data derived from head tissue provided evidence that a single member of the *S. calcitrans* Rh1 paralog cluster, named Rh1.1.1.1, likely maintains the ancestral function of Rh1 as the major motion detection specific opsin. This is based on comparison of its TPM values with that of the other Rh1 paralogs, as well as with those of the putative *S. calcitrans* opsins Rh5 and Rh6 (see Additional file 1: Table S11). The remaining *S. calcitrans* Rh1 cluster member genes showed relatively low to very low expression values (see Additional file 1: Table S11).

#### An amino acid modification at a tuning site differentiates two muscid Rh1 paralog subclusters

While exceptional for other higher Diptera, tandem duplicated LW opsin gene clusters have been found in mosquito and water strider species [210, 211]. In both cases, evidence of functional paralog diversification has been detected in the form of amino acid changes that affect opsin wavelength sensitivity (i.e., at tuning sites). Integrating data from butterflies and *Drosophila*, the water strider study identified one high confidence tuning site that very likely affects the blue vs green range wavelength specificity in LW opsins. The site is residue 17 based on the numbering system developed for butterflies, which corresponds to residue 57 in *Drosophila* Rh1 [211–213]. Based on this criterion, the three oldest *S. calcitrans* Rh1 gene cluster paralogs preserve the blue-shifted wavelength specificity (λ_max_ 480 nm in *Drosophila*) of the Rh1 singleton homologs of other dipteran species due to conservation of the ancestral methionine state at tuning site 17 (see Additional file 1: Figure S15). In contrast, the three younger *S. calcitrans* Rh1 paralogs share a leucine residue at tuning site 17, which is extremely rare across insect LW opsins. In a survey of over 100 insect LW opsins, it was detected only in the two corresponding Rh1 orthologs from *M. domestica* in addition to one in the distantly related species of thrips (Thysanoptera) [211]. The physicochemical similarity of the leucine at tuning site 17 in the three youngest *S. calcitrans* Rh1 paralogs relative to the pervasively conserved isoleucine residue at tuning site 17 in the green-sensitive Rh6 opsins (λ_max_ 515 nm in *Drosophila*) represents compelling evidence that this shared derived replacement substitution defines a green-sensitive subcluster in the *S. calcitrans* Rh1 paralog group (see Additional file 1: Fig. S15).

### Unique duplications in the *Stomoxys* yolk protein gene family

Cyclorrhaphan flies such as *Stomoxys* [214], *Drosophila* [215], *Musca* [216], *Calliphora* [217], and *Glossina* [218] utilize yolk proteins (YPs) as a primary source of nutrients for developing embryos. While many insects utilize YPs classified as vitellogenins, Cyclorrhaphan flies utilize an alternative class of proteins derived from lipase enzymes [219]. These proteins function as a source of amino acids and also as transporters of essential nutrients such as lipids and vitamins [220]. The number of yolk protein genes varies among higher fly species. The species-specific expansions/contractions observed within this class of genes may reflect reproductive demand within those species. Analysis of the *Stomoxys* genome identified eight putative *S. calcitrans* YP homologs relative to seven YP gene family members in *M. domestica* [94]; four of the seven *M. domestica* genes were annotated as part of this study.

**Fig. 6:**
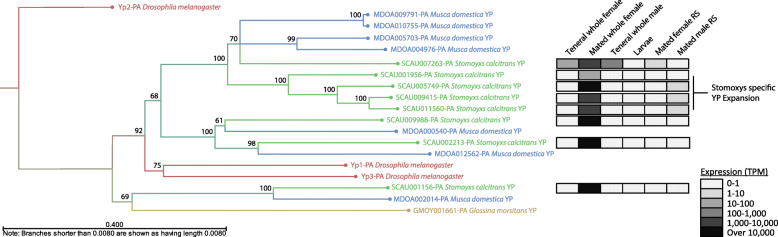
Maximum likelihood phylogenetic analysis of yolk protein genes from *Drosophila melanogaster*, *Glossina morsitans*, *Musca domestica*, and *Stomoxys calcitrans*. Gene sequences with significant homology were aligned using the ClustalO software package [221], and the alignment used to estimate a Maximum likelihood phylogeny in CLC Main Workbench (construction method: neighbor joining, Protein substitution model: WAG, Bootstrap analysis: 1000 replicates). Numerical annotations indicate bootstrap values for each branch point in the tree. Heat map of gene expression (transcripts per million, TPM) is based on RNA-Seq data (see Additional file 4). RS, reproductive system

Prediction of the evolutionary relationships between the predicted YPs from *S. calcitrans*, *M. domestica*, *G. morsitans*, and *D. melanogaster* by phylogenetic analysis (Fig. 6) suggests the yolk protein gene family expanded in *S. calcitrans* and *M. domestica* sometime after their divergence from *Drosophila*, which has three YP gene family members [222]. Of the *Stomoxys* and *Musca* specific YPs, three members are orthologous between the two species suggesting derivation from a common ancestor. However, the remaining yolk protein genes are paralogous and may originate from independent duplication events occurring after the divergence of the *Stomoxys* and *Musca* lineages. An alternative explanation is that these genes were originally orthologs, but have been altered via gene conversion resulting from homology based DNA repair mechanisms [223]. The lineage-specific expansions suggest that duplications in this gene family may confer a reproductive advantage by increasing reproductive capacity. In support of this role, all eight YP genes were detected by RNA-Seq in reproductively active *S. calcitrans* females, but not teneral females. Further, expression was not detected by RNA-Seq analysis of the female reproductive tract, which suggests these genes are expressed and translated in the fat body, secreted into the hemolymph and transported to the ovaries as observed in other higher flies (Fig. 6).

### Evidence of *Stomoxys* male-biased reproductive tract genes with putative seminal fluid function

**Fig. 7:**
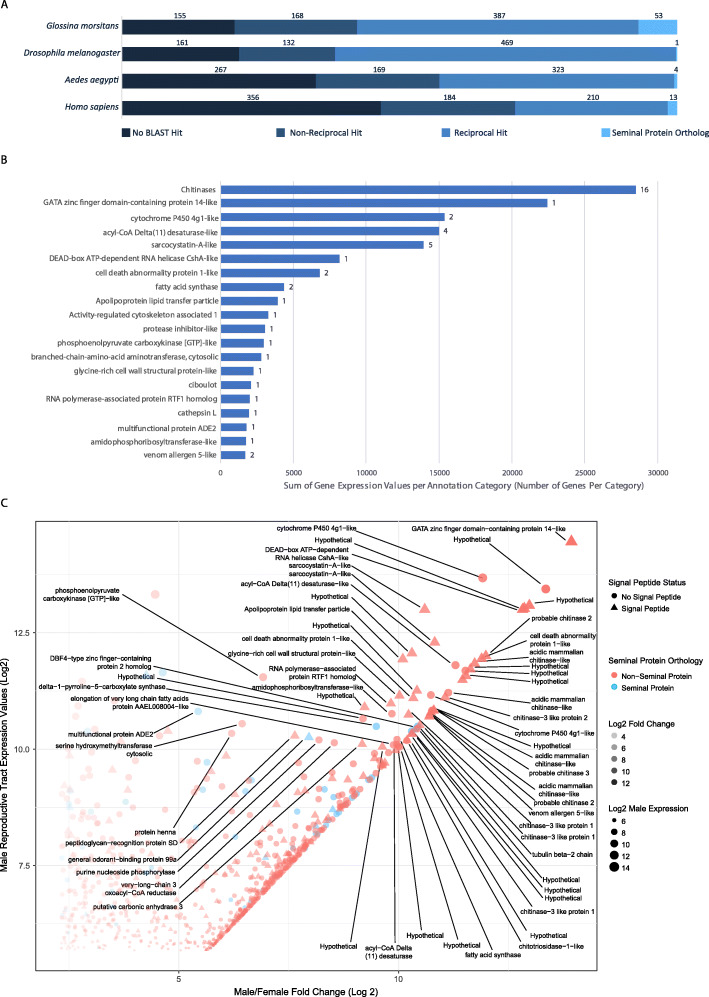
Analysis of male reproductive biased genes in *Stomoxys calcitrans*. **a** Results of reciprocal BLAST analysis of male reproductive tract biased genes. **b** Expression analysis of top 20 most abundant gene classes in the male reproductive tract RNA-Seq dataset versus the female reproductive tract, as annotated by BLAST best hits. Bar length represents combined expression values in TPM for the genes included in that category. Numbers associated with the bars represent the number of genes in that functional classification that had a male reproductive tract-biased expression. **c** Scatter plot of the 763 *Stomoxys* male reproductive biased genes. The plot shows on the *x*-axis - log_2_ fold change expression in males relative to females and the *y*-axis represents the log_2_ transformed expression value in TPM in the male reproductive tissue. Triangular points are genes predicted to contain signal peptides and blue points are genes with orthology to seminal proteins in other species. Genes with a log_2_ expression value above 10 and log_2_ Male/female fold change value above 5 are annotated with putative functional descriptions

Insect seminal fluid proteins are produced by the male reproductive tract and are transferred to females during mating. The seminal fluid proteins induce a post-mating response in females that results in behavioral and physiological changes including refractoriness to additional matings. The *S. calcitrans* reproductive tract is comprised of testes, vas deferens, and an ejaculatory duct, the latter of which has a region that produces the seminal fluid and serves an accessory gland function [224]. While Meola [224] observed storage of proteinaceous accessory gland material in the *S. calcitrans* ejaculatory duct, its composition has not been described. To identify male-biased reproductive tract genes encoding potential seminal proteins, RNA-Seq data derived from *S. calcitrans* male and female reproductive tract tissues were compared (Fig. 7). Genes in the male reproductive tract dataset were filtered to identify those expressed at least five-fold higher than in the female reproductive tract dataset. This analysis resulted in the classification of 763 genes with male reproductive tract-biased expression (see Additional file 10).

A reciprocal BLAST analysis identified orthologs of the male reproductive tract-biased genes in other species in which seminal proteins are characterized. These include *G. morsitans* [194], *D. melanogaster* [192, 225], *A. aegypti* [226], and *Homo sapiens* [227] (Fig. 7a). The overall number of identified orthologs corresponds roughly to the evolutionary distances between the species tested. However, these relationships did not hold for the number of gene orthologs associated with seminal function. Reciprocal analysis with *Drosophila* identified 469 1:1 orthologs of male reproductive tract-biased *S. calcitrans* genes, amounting to the largest number of orthologs identified between species included in this analysis. In contrast, of those 469 orthologs only one is associated with seminal fluid function in *D. melanogaster*. Comparison with *G. morsitans* identified the second highest number of orthologous proteins (*n* = 387). Of those, 53 were associated with seminal function, suggesting a greater similarity in the constitution of seminal secretions between *Glossina* and *Stomoxys* consistent with their closer phylogenetic relationship compared to *Drosophila*. Of note, none of the *S. calcitrans* male reproductive tract-biased proteins were orthologous to seminal proteins across all four species. In *Drosophila*, male-biased genes evolve at a faster rate, especially those expressed in reproductive tissues. These genes tend to lack identifiable orthologs relative to genes expressed in an unbiased pattern [228, 229]. There is evidence for this in *M. domestica* as well [94], and this phenomena is likely due to sexual selective pressure resulting in rapid evolution of male-biased genes [229]. However, one gene within this group encoding a catalase (XM_013259723) is orthologous to seminal protein genes in *Aedes*, *Glossina*, and *H. sapiens*. In *S. calcitrans*, two catalase genes were identified on separate scaffolds, one for which expression was detected in all life stages and tissues analyzed (XM_013257324) and the other, described above, that was detected primarily in the male reproductive tract. As catalases function to reduce oxidative stress, this finding could reflect a conserved mechanism that protects the sperm from oxidative damage.

The 763 *S. calcitrans* male reproductive tract-biased genes were annotated by BLAST and gene ontology analysis and then categorized by best hit annotation. Of those genes, 216 lacked significant BLAST hits or were homologous to hypothetical proteins with no functional associations. Of the remaining genes that had significant hits to annotated proteins, certain categories were highly expressed in terms of both the number of genes and the relative level of expression within the male reproductive tract (Fig. 7b, c). These genes were also tested for the presence of a secretion signal peptide within the predicted open reading frame to differentiate between secreted and non-secreted proteins. Approximately 22% of the male reproductive tract-biased genes (165/763) were found to include a predicted signal peptide. Gene ontology analysis of this group’s constituents revealed several significant functional enriched categories (see Additional file 10). The most highly significant groups included chitin binding, serine/cysteine-type endopeptidase inhibitors, metalloendopeptidases, antibacterial humoral response, and innate immune response among others. While the 1:1 orthologs to most of these genes do not act as seminal proteins in other organisms, they represent generally conserved functions in seminal proteins. Functions such as protease inhibition, immunity, protease activity and chitin binding have been characterized in the seminal secretions of other flies and insects [194, 226, 230–233].

Chitinases represented the most highly expressed functional group within the transcriptome and comprise 16 chitinase-like genes, of which 12 are clustered on scaffolds KQ079939 (seven genes) and KQ080089 (five genes). Of the approximately 37 genes annotated as chitinase in the *S. calcitrans* genome, the RNA-Seq results suggest these 16 are biased towards the male reproductive tract tissue (see Additional file 10). Chitinases confer anti-fungal activity in honey bee seminal secretions, preventing the transfer of pathogenic spores during copulation [234]. Although it is unclear if these have the same role, such antimicrobial properties would be beneficial to *Stomoxys* given the high probability of exposure to fungi in the moist and microbe rich substrates in which females oviposit. The second most highly expressed category consists of a single gene, XM_013245551, which is the most highly expressed gene in the male reproductive tract dataset. While it is annotated as a GATA zinc-finger domain containing protein, further analysis reveals little in the way of conserved domains to indicate its function. Interestingly, “domesticated” transposable elements tend to have a number of zinc-finger domains [235] and further studies are needed to evaluate whether this transcript may actually be a highly expressed transposable element. This analysis provides some insight into genetic associations with male reproductive functions in *Stomoxys* and further highlights several interesting targets for functional analysis in the future.

### Immunomodulatory and anti-hemostatic products are prominent in the *Stomoxys* sialome

**Fig. 8:**
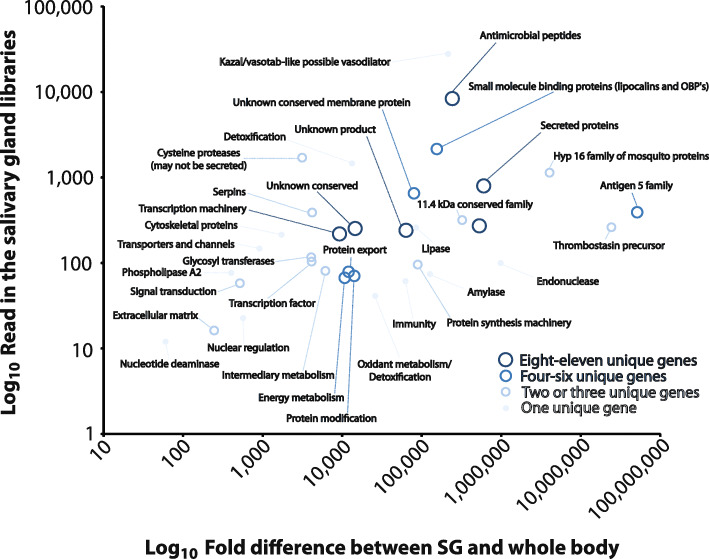
Analysis of salivary gland biased genes in *Stomoxys calcitrans*. Number of Illumina reads versus fold enrichment in the salivary gland related to the whole body. Each point represents the average among all genes in that specific category. Expression levels are based on results in Additional file 11

Blood-feeding insects salivate while probing their host skin for a blood meal. Development of a sophisticated salivary potion that disarms their hosts’ hemostasis is among the adaptations to blood feeding found in hematophagous animals [236, 237]. Blood clotting inhibitors, anti-platelet compounds, vasodilators, and immunomodulators are found in salivary gland homogenates or saliva of blood sucking arthropods [237]. To determine the genes associated with salivation, transcripts from male and female salivary glands (SG) were compared with those from teneral male and female whole bodies (WB). To be consistent with salivary gland transcriptome analyses completed in other blood-feeding arthropods, an Χ^2^ test was employed to identify those that were significantly over-expressed in salivary glands (Fig. 8), as in [238] (see Additional file 11). A subset of SG transcripts with 100-fold higher expression compared with teneral adults was analyzed in more detail (see Additional file 11). The SG 100-fold overexpressed set was comprised of 139 transcripts, 18 of which were found to be splice variants, or identical to other transcripts, as verified by their scaffold coordinates. The non-redundant set comprised of 121 transcripts was classified into three major groups: Putative Secreted, Putative Housekeeping, and Unknown; these groups were further classified into finer functional categories (see Additional file 11).

In congruence with *S. calcitrans* SG polypeptides previously sequenced from one-dimensional protein gel fragments [239], the antigen 5 family comprised 62% of the total reads mapped to the 121 SG-enriched transcripts (Fig. 8). Members of this family in *S. calcitrans* may function as inhibitors of the classical complement system [240]. Thrombostasin [241] members, which are precursors for anti-thrombin peptides previously identified in *S. calcitrans*, were represented by two transcripts. They accrued 29% of the reads and are strongly represented in the protein study [239]. The Hyp 16 family of peptides (unknown function, 4.9% of accrued reads) and one transcript encoding an endonuclease (1.2% of accrued reads) were also noted. Together, these groups of transcripts account for 97% of the reads that are over expressed in the salivary glands relative to the whole body of *S. calcitrans.*

There was a wide variety of other transcripts represented in the last 3% of the reads, and serine proteases, nucleotide deaminase, amylase, phospholipase A2, and lipases were found enriched in the *S. calcitrans* salivary gland transcriptome. These enzymes are also found enriched in other sialomes and their functions have been reviewed [237, 242, 243]. Two of eight serine proteases were found 5–15 times overexpressed in female salivary glands when compared to male glands. These two products produce best matches to vitellin-degrading proteases from *M. domestica* (XM_005191887.2) and may be indeed female enriched enzymes that were hitchhiked to the salivary set due to their similarities to overexpressed salivary enzymes. No other peptides were found above fivefold expressed in either salivary gland gender.

Several antimicrobial peptides appeared enriched in the *S. calcitrans* sialome, including lysozyme, attacins, defensins, diptericin, a GGY rich peptide, and sarcotoxin. Of these, only the GGY peptide and diptericin were identified in the previously reported Sanger-based *S. calcitrans* sialotranscriptome [239]. Given that a bloodmeal can be stored in the stable fly midgut for up to 48 h [244], these peptides may be a first line of defense to control microbial growth in the ingested meal. Regarding polypeptides with anti-proteolytic activity, in addition to thrombostasin precursors discussed above, two transcripts encode serpins (however, with very low expression) and one encodes a Kazal domain-containing peptide (accruing 0.3% of the reads). While serpins may modulate clotting and inflammation-related proteases, the Kazal domain peptide may be related in function to vasotab, a vasodilatory peptide from a tabanid fly [245].

Finally, 24 transcripts accruing 0.19% of the reads could not be functionally classified and were thus assigned to the “unknown” class. These include membrane proteins XP_013114823.1 and XP_013117270.1 that are over one thousand-fold over expressed in the salivary glands relative to whole body, the former of which was identified in the Sanger sialotranscriptome. Given their abundance in salivary gland tissue, these are attractive targets for gene disruption experiments to elucidate the contribution of these proteins to the salivary function of *S. calcitrans*.

### Expanded cytochrome P450 gene family suggests enhanced metabolic detoxification in *Stomoxys*

**Fig. 9:**
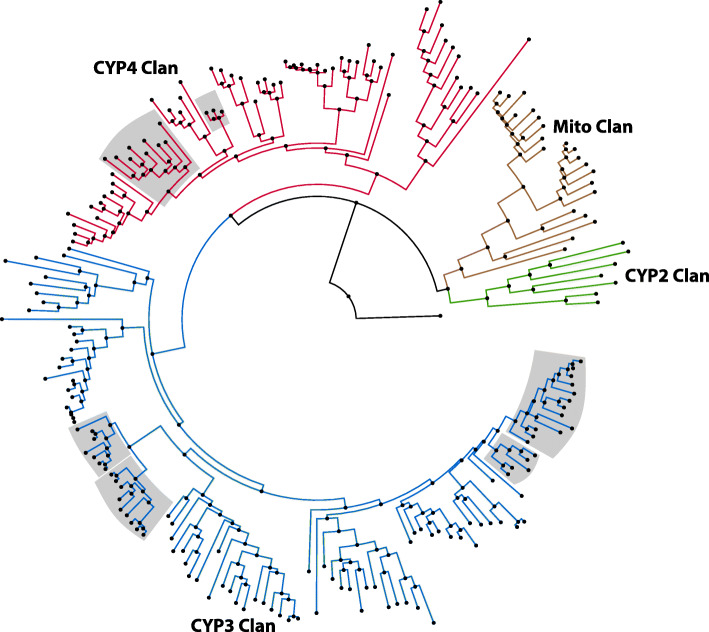
Phylogenetic analysis of cytochrome P450 genes from *Stomoxys calcitrans*. Amino acid sequences from each family were aligned with the MUSCLE algorithm [201], and the alignments trimmed with the trimAl tool using the –strictplus option [266]. The trimmed alignment was used to construct a maximum likelihood phylogeny, rooted with *Mus musculus* CYP51 as the outgroup, with the web server version of IQ-TREE software (best-fit substitution model, branch support assessed with 1000 replicates of UFBoot bootstrap approximation; bootstrap percentages reported [114]). The CYP clades are identified by different colored lineages, and CYP gene clusters that are found in tandem within the genome are shaded in gray. P450 gene names were assigned based on comparative analyses (see Additional file 13), and the full phylogenetic tree can be found in Additional file 1: Figure S21

Various detoxification mechanisms have evolved in insects to enable their survival upon exposure to environmental toxins. Metabolic detoxification is mediated by members of the carboxylesterase and glutathione-S-transferase gene families (identified and described from *S. calcitrans*; see Additional file 1: Gene families associated with metabolic detoxification, Figures S16 - S19 [246–263] and Additional file 12), as well as the cytochrome P450 (CYP) gene family. Arthropod CYPs have diverse roles in insect physiology, including ecdysteroid biosynthesis and xenobiotic detoxification [264, 265]. The CYP gene family size varies among insects, with dipterans having large arrays (i.e., 145 in *Musca*, 86 in *Drosophila*, and 77 in *Glossina*). The 214 *S. calcitrans* CYPs that were identified from genome analysis encode representatives from each of the CYP clans that are typically found in insects (i.e., mitochondrial, CYP2, CYP3, and CYP4), and they represent a substantial increase in number relative to other sequenced Dipteran genomes (Fig. 9; see Additional files 13 and 14). The *S. calcitrans* mitochondrial and CYP2 clans contain orthologs of the Halloween genes that mediate the pathway for ecdysteroid biosynthesis, namely Cyp306A1, Cyp302A1, Cyp314A1, and Cyp315A1, and the mitochondrial clan (21 genes) contains tandemly arranged genes along three scaffolds: nine CYP12A genes on KQ080363 and seven CYP12G genes on KQ080439 and KQ082110. As in *M. domestica*, expansions in *S. calcitrans* were primarily observed in clans 3 and 4. The CYP4 clan (62 genes) was represented by the CYP4 (51 genes) family, while the CYP3 clan (107 genes) comprised the largest increase in number of CYPs in *Stomoxys*, predominated by the CYP6 (81 genes) and CYP9 (16 genes) families. Together, members of the CYP4 and CYP6 families represent 62% of the *S. calcitrans* CYPs, which is comparable to *M. domestica* [94].

At least three families of tandemly arranged CYP genes were present in the *S. calcitrans* genome (Fig. 9, shaded) and illustrate duplication and loss processes in this superfamily. The *S. calcitrans* and *M. domestica* CYP9F genes encode 15 and 4 proteins, respectively, and they are tandemly arranged on *Stomoxys* scaffold KQ080085 and *Musca* scaffold KB855374 with microsynteny of flanking genes DNA ligase 3, fatty acyl co-A reductase, and a cluster of glutathione-S-transferase delta genes (see Additional file 1: Figure S20). A similar arrangement is found for the two *D. melanogaster* CYP9F proteins on chromosome 3R, and phylogenetic comparison suggests an expansion in *Stomoxys* and *Musca* that was lost in *Drosophila* (see Additional file 1: Figure S20-A). Genes encoding 24 CYP6A proteins in *S. calcitrans* are clustered on two scaffolds (KQ080770 and KQ080111) that are likely on one array in the genome, and a comparable cluster of genes encoding 19 CYP6A proteins on two *M. domestica* scaffolds (KB855857 and KB859418) were identified. Phylogenetic comparison with the 13 CYP6A *D. melanogaster* genes depicted expansion in *Stomoxys* and *Musca* relative to *Drosophila* CYP6A2, but a loss in *Drosophila* relative to 15 *Stomoxys* and 8 *Musca* CYP6As (see Additional file 1: Figure S20-B). While upregulation of genes in the CYP4, CYP6, and CYP9 families have been associated with resistance to spinosad and pyrethroid insecticides in *Musca*, *Anopheles*, and *Drosophila* [267–269], this has yet to be investigated in *Stomoxys*. Regardless, the large CYP gene family suggests *Stomoxys* may have an enhanced capacity for metabolic detoxification.

### Evidence for transcription factors with putative role in regulation of reproduction and salivation

**Fig. 10:**
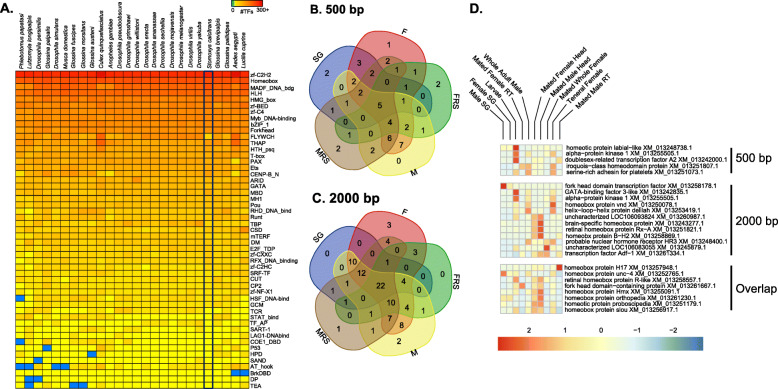
Transcription factors associated with *Stomoxys calcitrans*. **a** Number of transcription factors identified in *S. calcitrans* compared to other flies. **b**, **c** Overlap between transcription factors with increased binding sites in differentially expressed genes that have noted expression in the same tissue (F, teneral female; FRS, female reproductive system; M, male; MRS, male reproductive system; SG, salivary glands). **d** Expression of specific TFs associated with female, male, and salivary glands among multiple tissues and developmental stages

To determine transcription factors (TFs) that might control specific gene expression profiles in *S. calcitrans*, TF-encoding genes were first predicted by identifying putative DNA binding domains (DBDs), using a previously described approach [40, 70]. These analyses resulted in 837 predicted TFs, with the highest number coming from the C_2_H_2_ zinc finger and homeobox structural families (Fig. 10), consistent with previously analyzed insect genomes [40, 69, 70]. DNA binding motifs were subsequently predicted for as many of these putative TFs as possible using a previously developed method [270], and “inferred” motifs were identified for the *S. calcitrans* TFs. For example, the DBD of the uncharacterized XP_013101333 protein is 92.3% identical to the DBD of the *D. melanogaster* gene *cropped* (FBgn0001994). Since the DNA binding motif of *cropped* has already been experimentally determined, and the cutoff for the bHLH family of TFs is 60% (see the “Methods” section), we can infer that XP_013101333 will have the same binding motif as *cropped*. This procedure resulted in inferred motifs for 285 of the *S. calcitrans* TFs (34%).

TF binding site motif enrichment was then completed using promoter regions for groups of genes with similar gene expression patterns in our RNA-Seq datasets. Promoters were defined as either 500 or 2000 bp upstream of the predicted transcription start site for each gene and gene set/motif pairs were filtered as described in the “Methods” section. Expression of each TF was verified in specific tissues using our RNA-Seq datasets (see Additional file 15). Based on these criteria and comparative analyses between samples, seven and nine TFs, respectively, were enriched in SG tissues for the 2000 bp and 500 bp promoter regions (Fig. 10). Based on the 500 bp promoter regions, two specific TFs, *proboscipedia* (XM_013251179) and *orthopedia* (XM_013261230), likely regulate SG-based transcript expression. These two TFs have been associated with head and salivary development in *Drosophila* [271, 272], and the increased binding sites and specific expression profile suggest a role in *S. calcitrans* saliva production.

Male- and female-enriched analysis based on stage and tissue specific RNA-Seq datasets identified TF targets in each of the 500 bp and 2000 bp promoter regions (Fig. 10). The four most likely TFs associated with female specific genes are XM_013251807.1 (*iroquois-class homeodomain protein*) and XM_013252765.1 (*Unc4 homeodomain protein*) based on the 500 bp promoter region and two other likely homeodomain proteins, XM_013245879.1 (uncharacterized) and XM_013261334.1 (uncharacterized), in the 2000 bp regulatory region. The latter have high TPM values in females and female reproductive tract tissues (Fig. 10). Two TFs within the 500 bp promoter region were for male enriched genes XM_013258869.1 (*BarH-2*) and XM_013251073.1 (uncharacterized), both of which are highly expressed in *S. calcitrans* teneral males, male heads, and/or the male reproductive tract tissue. When expanded to the 2000 bp promoter region, two additional putative TFs related to male enriched genes were XM_013260987.1 (uncharacterized) and XM_013257948.1 (*drop*), which are both highly expressed in male samples. Functional characterization of these TFs is warranted, as they could provide novel targets for the control of stable fly reproduction or the prevention of feeding, including development of genetically modified strains based on reproductive tract-specific regulatory elements.

## Conclusions

This study advances the knowledge of stable fly genomics and genetics to the breadth of other non-model, but extremely important, dipterans such as tsetse and house flies. Analysis of the genome sequence of stable fly males coupled with the life-stage and tissue-specific gene expression datasets revealed unique aspects of muscid fly biology that will guide future research within the livestock pest community. The distinct increase in the number of chemosensory receptors that are putatively involved in bitter taste perception is intriguing (Figs. 3 and 4), as it may support the stable fly’s cosmopolitan distribution by enhancing avoidance behaviors and enabling a more discerning host and ovipositional substrate selection process [12, 19, 20]. This distribution is likely further supported by putative enhanced metabolic detoxification mediated by an increase in number of CYPs encoded by the genome (Fig. 9). Downstream functional analyses are critically essential to clarifying these roles. Importantly, this study provides the resources to support development of novel control approaches for this livestock pest. Identifying stable fly chemosensory factors that invoke aversion could inform development of species-specific behavior modifying compounds and/or strategies [273], while the unique stable fly catalog of putative seminal proteins reported here (see Additional file 10) could be targeted for development of reproductive inhibitors. Conventional sterile insect technique methods are not sustainable for a stable fly control program, as stable flies are broadly dispersed in areas where they are problematic and both sexes of *Stomoxys* blood-feed. A continuous release of large numbers of irradiated, sterile males would be required to achieve successful population reduction [274–276]. Gene drive-based systems are attractive technologies, and availability of the genome enables identification of regulatory regions to develop such strains. Lastly, the recent analysis of sex chromosome evolution in stable flies and the recently sequenced horn fly underscores the unique opportunity that this genomic resource provides to enable future comparative genome analyses [49, 277].

## Methods

### Genome sequencing, assembly, and annotation

Total genomic DNA was isolated from pooled, teneral adult males (*n* = 120) of a *Stomoxys calcitrans* line that resulted from single paired mating for 7 generations (Sc8C7A2A5H3J4). High quality/high molecular weight DNA was isolated from pooled flies using the Genomic-tip purification column and the associated buffer kit (QIAGEN, Valencia CA), and samples were processed according to the protocol for tissue-based DNA extraction. The pooled DNA isolates were utilized for sequencing on Illumina® HiSeq2000 instruments. The sequencing plan followed the recommendations provided in the ALLPATHS-LG assembler [278]. Using this model, we targeted 45x sequence coverage each of fragments (overlapping paired reads ~ 180 bp length) and 3 kb paired end (PE) sequences as well as 5x coverage of 8 kb PE sequences. The first draft assembly scaffold gaps were closed where possible with mapping assembly input sequences (overlapping paired reads ~ 180 bp length) and local gap assembly [279]. Contaminating sequences and contigs 200 bp or less were removed. The genome assembly, Stomoxys_calcitrans-1.0.1, was made publicly available in the Genbank sequence database, accession number GCF_001015335.1. All sequence files associated with the project can be accessed on Genbank under BioProject PRJNA188117 ([280]; see Additional file 1: Table S1).

Automated gene annotation of the *Stomoxys* genome assembly (Stomoxys_calcitrans-1.0.1) was completed using the NCBI Eukaryotic Genome Annotation Pipeline, version 6.4 with supporting RNA-Seq from existing transcripts in the expressed sequence tag [281] database and a de novo assembled transcriptome (Trinity-v2; transcriptome shotgun assembly (TSA) sequence database, Accession number GDIM00000000.1). The resulting *Stomoxys* Annotation Report 100 can be accessed at https://www.ncbi.nlm.nih.gov/genome/annotation_euk/Stomoxys_calcitrans/100/

The Web Apollo tool [282], adopted by VectorBase [283], was used by our community to review and edit automated gene predictions. Edited gene models were incorporated into a community annotation patch build, ScalU1.6 (release date: November 5, 2020).

### Lateral gene transfer prediction

A DNA-based computational pipeline was used to identify “contaminating” bacterial scaffolds and bacterial to *Stomoxys* lateral gene transfer candidates in the *Stomoxys* genome assembly. The pipeline was originally developed by David Wheeler and John Werren [284] and has subsequently been modified. Details of the pipeline are provided in [81], and the procedure is summarized here. First, genome scaffolds were broken into 1000-bp units, which are screened against a bacterial genome database (see Additional file 16) containing over 2100 representative bacterial species. Scaffolds were then evaluated for proportion of bacterial matches along their length – those with greater than 20% bacterial matches were identified as likely bacterial scaffolds in the insect genome assembly and were manually examined for average coverage depth compared to all the scaffolds in the genome. That analysis supported twelve very small scaffolds (1028–3546 bp) as bacterial contamination (see Additional file 1: Table S6). The small size and low read depth compared to genome scaffold average supports the conclusion that these represent trace bacterial contamination (see Additional file 1: Tables S6 and S7).

To examine the assembly for candidate bacterial LGTs, analyses were conducted on scaffolds of greater than 100 kb in length, because confidently assigning a candidate LGT requires confirmation of eukaryotic flanking sequences to the candidate. Positive bacterial hits in each 1000-bp region with bit scores greater than 50 were compared to an “eukaryotic” database (see Additional file 16), which contains transcripts from the following eukaryotes: *Xenopus*, *Daphnia*, *Strongylocentrotus*, *Mus*, *Homo sapiens*, *Aplysia*, *Caenorhabditis*, *Hydra*, *Monosiga*, and *Acanthamoeba*. The purpose of this step is to exclude highly conserved sequences that are shared between bacterial and eukaryotes from further analysis. Focus was then placed on strong LGT candidates with bitscore > 75 in the bacterial match and zero bitscore in the eukaryotic match. Regions were combined when adjacent 1Kb fragments each had a zero “animal” match and a bacterial match to the same or similar bacterial source. The 1Kb fragments were also screened against transcripts from seven arthropod species to identify possible conserved arthropod genes (see Additional file 16). The initial candidate LGTs were then manually curated, through a series of steps including (1) BLASTn to NCBI nr database, (2) BLASTx to NCBI protein database, (3) examination of flanking regions in the scaffold to confirm presence of flanking eukaryotic (insect) orthologous sequences and gene models, (4) removal of matches due to repetitive DNA, and (5) examination of RNA-Seq datasets for evidence of expression. In addition, LGT candidates were examined for sequencing read depth in the candidate LGT and flanking 1 kb up and downstream regions to determine if there were large changes in read depth across the junction. Sequencing reads were aligned to the reference genome using BWA v 0.7.17 [285]. Paired end alignment was called using the -mem function and aligned to the reference genome using default parameters. The coverage software program Mosdepth [286] was then used to calculate the average 50 bp read depth spanning the LGT and its junctions. Coverage ratio was calculated as the average read depth spanning a 50 bp window divided by the average 50 bp window read depth of the entire scaffold.

### RNA isolation and sequencing

A variety of developmental stages and dissected tissues were collected for total RNA isolation, and the accession numbers and metadata associated with these samples is summarized in see Additional file 1: Table S1. Specifically, RNA collected from whole females (teneral and mated, reproductive), whole males (teneral), male reproductive tracts, female reproductive tracts, male heads (fed, mated), female heads (fed, mated), and a single third instar larva were examined to assist in addressing core questions of this study and to assist with genome annotation. RNA was extracted with the use of TRizol. DNA contamination was reduced via DNase treatment according to methods previously described [287, 288]. Poly(A)+ RNA was isolated, then measured with the Agilent Bioanalyzer for quality and only those samples with a minimum RIN score of 7 were used to build non-normalized cDNA libraries using a modified version of the Nu-GEN Ovation® RNA-Seq System V2 (http://www.nugeninc.com). We sequenced each cDNA library (0.125 lane) on an Illumina HiSeq 2000 instrument (~ 36 Gb per lane) at 100 base pair length. RNA-Seq datasets used in gene prediction have been deposited to the NCBI Sequence Read Archive under the accession codes SRX995857–5860, SRX229930, SX229931, and SRX275910 (see Additional file 1: Table S1).

### RNA-Seq analyses

In conjunction with the genomic sequencing, RNA-Seq analyses were performed to examine specific transcript differences between different stages and tissues (see Additional file 4). RNA-Seq analyses were conducted based on methods in Benoit et al. [287] and updated according to Rosendale et al. [288, 289]. RNA-Seq datasets analyzed are summarized within Table S1. The main goals for analyzing these datasets were to (1) determine male, female, and larva-enriched gene sets, (2) identify reproductive specific genes, and (3) establish chemosensory genes associated with sexes and specific tissue. Quality of each RNA-Seq dataset was assessed with FastQC prior to analyses, and RNA-Seq datasets were trimmed and low-quality reads were removed with trimmomatic [290]. Each dataset was mapped to the predicted gene set (NCBI Annotation Release 100) for *S. calcitrans* using CLC Genomics with each read requiring at least 95% similarity for over 60% of the read length with only two mismatches allowed. Splicing was allowed as long as 60% of each read mapped to the respective mRNA sequence. Read alignments were converted to per million mapped to allow comparison between RNA-Seq data sets with varying coverage (see Additional file 1: Table S1). Expression was based upon transcripts per million (TPM) and fold changes were determined as the TPM in one sample relative to the TPM of another dataset [288]. Significant enrichment was based on a Baggerly’s test (compares proportional changes between groups) followed by Bonferroni correction at 0.01 (number of genes x α value). This similar, stringent analysis was used on previous samples with only a single replicate and proved to be useful in the identification of genes of interest associated with specific developmental stages and sexes [41, 70]. RNA-Seq analyses are summarized in Additional file 4.

Transcripts in the official gene set were identified by BLASTx searching against an NCBI non-redundant proteins database for arthropods with an expectation value (*e* value) of at least 0.001. Gene ontology assessment for specific groups were conducted by the use of g:Profiler following conversion of *Stomoxys* gene IDs to *D. melanogaster* gene IDs [291]. The proteome of the stable fly was organized on a hyperlinked spreadsheet (see Additional file 2) with accompanying information (e.g., the presence or absence of signal peptide indicative of secretion, presence of transmembrane domains, and similarities to several databases). Expression values present within this sheet are based on the RNA-Seq data sets (see Additional file 1: Table S1) and analyzed according to previously described methods [238, 243].

### Reverse transcription quantitative PCR (RT-qPCR) verification of RNA-Seq results

Since each RNA-Seq dataset is based on a single replicate, the results were validated by RT-qPCR (see Additional file 1: Figure S2, Table S5). RNA-Seq and RT-qPCR results showed Pearson correlation of 0.8643, indicating the RNA-Seq results are valid. Twenty-five transcripts were randomly selected to evaluate correlation between log_2_ fold changes of RT-qPCR versus RNA-Seq. Total RNAs were isolated from tissues to represent those used for RNA-Seq {i.e. female or male heads (7d fed, mated), female or male reproductive systems (7d fed, mated, dissected), adult female or male (whole, teneral), adult female or male (whole, 7d fed, mated), third instar larvae}. Samples were placed in TRIzol™, macerated, and stored at − 80 °C until isolation using the Zymo Direct-zol**™** method (Zymo Research, Irvine CA) with on-column DNAse treatment (TURBO DNase, ThermoFisher Scientific, Waltham MA). cDNA templates were synthesized from 500 ng total RNA in a 20ul volume using SuperScript**®** III reverse transcriptase (ThermoScientific) primed with a dTV_n_ oligonucleotide. Primers for RT-qPCR were designed with Beacon software to span exon-intron junctions (see Additional file 1: Table S5), and primer efficiencies were estimated using serially diluted cDNAs. Reactions were prepared in a 20ul volume consisting of 250 nM each of the forward and reverse primers, cDNA from 25 ng RNA, and the iTaq™ Universal SYBR® Green Supermix (Bio-Rad Laboratories, Hercules CA). Reactions were run in triplicate on a LightCycler**®** 96 System (Roche Life Sciences, Indianapolis IN). Data were analyzed using the 2^-ΔΔ*CT*^ method incorporating primer efficiency data [292], and all values were normalized to the *S. calcitrans* ribosomal protein S3 reference gene *RpS3*. Pearson’s correlation of log_2_ fold change for RNA-Seq and RT-qPCR results was calculated using GraphPad Prism version 7.00 for MacOSX (GraphPad Software, La Jolla CA).

### OrthoDB analysis

The OrthoDB hierarchical orthology delineation procedure was employed to predict orthologous groups (OGs) of genes across 87 arthropods for OrthoDB v8 [63]. Briefly, protein sequence alignments were assessed to identify all best reciprocal hits (BRHs) between genes from each pair of species, which are then clustered into OGs following a graph-based approach that starts with BRH triangulation. The annotated proteins from the genome of *Stomoxys calcitrans* were first filtered to select one protein-coding transcript per gene and then mapped to OrthoDB v8 at the Diptera (37 species), Endopterygota (72 species), Insecta (80 species), and Arthropoda (87 species) levels. OrthoDB orthology mapping uses the same BRH-based clustering procedure used to build the OGs but only allowing proteins from the mapped species to join existing OGs. Gene ontology assessment for specific groups were conducted by the use of g:Profiler following conversion of *Stomoxys* gene IDs to *D. melanogaster* gene IDs through BLASTx comparison [291].

### Bacterial community analysis

To identify culturable bacterial communities harbored by adult stable flies, fly specimens were collected at each of four Texas dairies in April and June 2015 (Lingleville and Comanche, Texas), and total bacterial isolates from the collections is reported. Twenty flies per site per date were collected by aerial sweep nets in the area surrounding each dairy’s milking parlor. Within 4 h, whole flies were surface sterilized in 1% sodium hypochlorite for 15 min, followed by two washes in 70% ethanol and three rinses in sterile water. Individual flies were macerated in Butterfield’s phosphate buffer, and the homogenate was diluted and plated on tryptic soy agar. Individual, morphologically distinct colonies were selected, suspended in Butterfield’s phosphate buffer, and the DNA isolated by rapid boiling. These DNAs were used as template in 16S PCR amplification with a universal primer pair (16SEub_61F: 5′ – GCTTAACACATGCAAG – 3′; 16SEub_1227R: 5′ – CCATTGTAGCACGTGT – 3′). Individual amplicons were sequenced in both directions and the sequences assembled (*n* = 281). Data were analyzed in mothur, v 1.38.1 [281], including UCHIME to detect and remove 19 chimeras, resulting in 262 isolates. Sequences were aligned using the silva.seed_v132.align file, distance matrices calculated, and isolates classified at a 97% similarity cut-off, average neighbor, silva.seed_v132.align/.tax. Phyla and genera for each isolate is reported (see Additional file 5).

### Analysis of immune system gene families

Two computational approaches were combined with targeted manual annotation to produce an annotation of immune-related genes and gene families in the *S. calcitrans* genome. First, an initial list of *S. calcitrans* gene models was generated that likely have an immune function based on homology to annotated and functionally characterized *D. melanogaster* genes. Homologous genes were identified based on OrthoDB groups and include both orthologs and paralogs (e.g., every *S. calcitrans* gene in an OrthoDB group that includes a *D. melanogaster* immune-related gene is annotated as immune-related). To complement these homology-based annotations, all predicted *S. calcitrans* proteins were screened for similarity to HMM profiles of well-characterized Dipteran immune-related protein families (see Additional file 6) [93, 100], using hmmscan (from the HMMER software package). Finally, because antimicrobial peptides can be difficult for computational pipelines to properly annotate (due to their small size), we also include some manual annotation of *S. calcitrans* AMPs (see Additional file 6).

### Chemosensory gene family identification and phylogenetic analysis

tBLASTn searches of the genome assembly were performed with gustatory receptors (GR) from *M. domestica*, *D. melanogaster*, and, where relevant the medfly, *C. capitata* [42], and with all newly identified *Stomoxys* GRs. Models were built primarily in the WebApollo server at VectorBase, using a combination of existing automated models, RNA-Seq information from multiple lifestages, tissues, and sexes, and comparisons with the *Musca* and *Drosophila* Gr gene structures. A few models with regions missing in assembly gaps were repaired using raw genomic and/or RNA-Seq reads. Pseudogenes were translated as best possible to provide an encoded protein that could be aligned with the intact proteins for phylogenetic analysis. A 200 amino acid minimum was enforced for including pseudogenes in the analysis (roughly half the length of a typical GR), and there are several shorter fragments of genes that were not included in the analysis. All *Stomoxys* GRs were aligned in CLUSTALX v2.1 [293] using default settings with the GRs of *D. melanogaster* [118] and *M. domestica* [94]. Problematic gene models and pseudogenes were refined in light of these alignments. The final alignment was trimmed using TrimAl v1.4 [266] with the “strict” option. Phylogenetic construction was performed by maximum likelihood analysis using the PHYML v3.0 webserver with default settings, implementing an automatic model selection parameter [294]. The resultant tree was formatted and colored using FigTree v1.4.2 (http://tree.bio.ed.ac.uk/software/figtree/), while the final version with labels was made in Adobe Illustrator. Analysis of the Ionotropic Receptor (IR) family in *S. calcitrans* was generally similar to that for the GRs, except that pseudogenes were only included if they encoded at least 50% of the length of a related intact IR given that IRs vary considerably in length. The GR maximum likelihood tree was rooted by declaring the distantly-related and divergent carbon dioxide and sugar receptor subfamilies as the outgroup, while the IR tree was rooted by declaring the Ir8a/25a lineage as the outgroup.

Odorant receptor (OR), odorant binding protein (OBP), and chemosensory protein (CSP) sequences from *D. melanogaster* and *M. domestica* were used in tBLASTn searches to identify orthologs in the *Stomoxys calcitrans* 1.0.1 genome assembly. Models were built primarily in the WebApollo server at VectorBase, using a combination of existing automated models, RNA-Seq information from multiple lifestages, tissues, and sexes, and comparisons with the *Musca* and *Drosophila* gene models. Amino acid sequences from each family were aligned with the MUSCLE algorithm [201], and the alignments trimmed with the trimAl tool using the –strictplus option [266]. The trimmed alignment was used to construct a maximum likelihood phylogeny with the web server version of IQ-TREE software ([114]; best-fit substitution model, branch support assessed with 1000 replicates of UFBoot bootstrap approximation). The OR tree was rooted with the highly conserved ORCO. OBP domains of dimer OBPs were separated for phylogenetic analysis and labeled “a” and “b,” and the Plus-C OBPs were not included in assembling alignments for tree construction.

### Opsin identification and analysis

For preparation of the global opsin gene tree, *S. calcitrans* sequences were collected by tBLASTn searches against the genome sequence draft version 1.0.1 (GCF_001015335.1). A multiple sequence alignment was generated with MUSCLE [201] and variable sites were filtered using Gblocks with least stringent settings [65]. Bayesian tree analysis was performed out with MrBayes v3.2.6 [295] in the CIPRES Science Gateway V 3.3 environment [296], applying the GTR model of protein sequence evolution and correcting for across site substitution variation with a four rate category gamma distribution. A bootstrapped maximum likelihood tree was estimated in MEGA version 6.0 [203] applying the Jones-Taylor-Thornton (JTT) model of amino acid sequence evolution and assuming Gamma Distributed substitution rates across sites with three categories. For Bayesian analysis of the calyptrate expansion of Rh1 opsins, protein sequences were aligned with webPRANK [297]. Ambiguous alignment regions were filtered using TrimAl (v. 1.3) [266] as implemented on the Phylemon 2.0 server [298] applying User defined settings (Minimum percentage of positions to conserve: 10, Gap threshold: 0.9, Similarity threshold: 0.0, Window size: 1.0).

### Yolk protein gene identification and phylogenetic analysis

Annotated yolk protein gene sequences from *Drosophila melanogaster*, *Glossina morsitans*, and *Musca domestica* were used to identify yolk protein ortholog sequences from the *Musca domestica* and *Stomoxys calcitrans* predicted transcriptomes available at VectorBase ([283]; www.vectorbase.org). Gene sequences with significant homology were aligned using the ClustalO software package [221]. The alignment was used to generate a maximum likelihood phylogeny using the tree generation software included in the CLC Main Workbench (Qiagen, Redwood City CA) software package using the following settings (construction method: neighbor joining, Protein substitution model: WAG, Bootstrap analysis: 1000 replicates). Sequences with homology closer to lipase enzyme sequences (the ancestors to yolk protein genes) that form outgroups relative to annotated yolk proteins were removed from the alignment and the phylogeny was recalculated.

### Identification of male reproductive tract-biased genes and reciprocal orthology analysis

RNA-Seq libraries from male and female reproductive tissues of fed, mated *Stomoxys calcitrans* adults were used in this analysis. TPM values from the read mapping of these libraries against the putative *Stomoxys* transcriptome from the male and female reproductive tissues were compared to identify genes with a male/female expression ratio of at least 5 and a minimum TPM value of 50 in the male reproductive tract. The sequences meeting these criteria were extracted from the *Stomoxys* transcriptome and then further filtered based on the presence of a secretory peptide. The narrowed list was categorized by gene ontology analysis using the R package topGO v 2.40 [299], providing statistical analysis of the categorization. Orthologous sequences for these genes were identified using a reciprocal hit analysis using the BLAST+ [300] software package against the predicted transcriptomes from *Glossina morsitans*, *Drosophila melanogaster*, *Aedes aegypti*, and *Homo sapiens*. Best hits from each of these transcriptomes from these species against male biased *Stomoxys* genes were extracted and used to BLAST back against the predicted *Stomoxys* transcriptome. BLAST output was parsed to detect *Stomoxys* genes with reciprocal hits (see Additional file 10).

### Salivary gland RNA-Seq analysis

Sixty salivary gland pairs were dissected from 7d fed, adult female and male stable flies. Tissues were placed in TRIzol™, macerated, and stored at − 80 °C until isolation using the Zymo Direct-zol**™** method (Zymo Research) with on-column DNAse treatment (TURBO DNase, ThermoFisher Scientific). Resulting cDNA libraries were sequenced on an Illumina HiSeq 2000 instrument, and a total of 24.8 M and 47.6 M, 75 bp, single end reads were obtained. The salivary gland RNA-Seq dataset is based on a single replicate. To be consistent with previous studies on the sialome of insect vectors, salivary gland RNA-Seq analyses were conducted using a published pipeline [301–303]. Briefly, the reads from four RNA-Seq libraries (male and female salivary glands, as well as teneral male and female whole bodies) were mapped to the *S. calcitrans* predicted gene set. To further be consistent with SG analyses completed in other blood-feeding arthropods, an χ^2^ test was employed to identify those that were significantly over-expressed in SG relative to teneral whole bodies, as in [238] (see Additional file 11). Normalized fold-ratios of the sample reads were computed by adjusting the numerator by a factor based on the ratio of the total number of reads in each sample and adding one to the denominator to avoid division by zero. Expression results for the salivary glands showed high level of correlation between males and females (Pearson = 0.93). The complete dataset was organized in a hyperlinked spreadsheet as previously reported [238] and is provided in Additional file 2.

### Transcription factor analyses

To assess potential transcription factors regulating tissue and sex-specific expression, TFs were identified according to previously developed methods in other insect systems [40, 70]. DNA binding motifs were then predicted for as many of these putative TFs. In brief, the percent of identical amino acids was calculated between each *S. calcitrans* TF and each eukaryotic TF with a known motif, with values exceeding a TF family-specific threshold resulting in “inferred” motifs for the *S. calcitrans* TFs. TF binding site motif enrichment was then completed using promoter regions for groups of genes with similar expression patterns. Promoters were defined as either 500 or 2000 bp upstream of the predicted transcription start site for each gene. The search was restricted to gene set/motif pairs with significant enrichment based on RNA-Seq analysis using Baggerly’s test followed by Bonferroni correction at 0.01, as described earlier. Gene set/motif pairs were further filtered to cases where (1) the given motif was present in at least 60% of the promoters of the gene set, (2) the given motif was present in less than 20% of all gene promoters, and (3) the difference between the presence of the motif in the gene set and promoters of all genes exceeded 40%. Expression of each TF was verified in specific tissues using our RNA-Seq datasets (see Additional file 15). Enriched TF binding motifs were identified in the 500 and 2000 bp regions upstream of the putative transcription start site using the HOMER tool [304] supplemented with the *Stomoxys* inferred binding motifs obtained from the CisBP database (build 0.90).

## Supplementary Information

**Additional file 1: **Main supplementary text file, including supplementary **Tables S1-S12** and supplementary **Figures S1-S21;** **Table S1.** RNA-Sequencing and Whole Genome Sequencing Accession Numbers and Statistics. **Table S2.** Summary of Dfam repeat elements with > 1000 copies including number of unique elements for each family of elements and total number of genomic copies for each family. **Table S3.** Detailed list of repeat elements with total genomic copy number > 1000. **Table S4.** Autophagy genes identified from the *Stomoxys* genome**. Table S5.** Validation of *Stomoxys* transcript expression by RT-qPCR. **Table S6.** Bacterial contaminating scaffolds located in the *Stomoxys* genome assembly**. Table S7.** Predicted lateral gene transfer events identified from the *Stomoxys* genome. **Table S8**. Components of the immune deficiency, Toll, and JAK/STAT pathways identified from the *Stomoxys* genome**. Table S9.** Manually annotated *Stomoxys* immune system gene family members. **Table S10.** RNA-Seq normalized expression values for transcripts annotated as antimicrobial peptides. **Table S11.** *Stomoxys* opsin gene compilation. **Table S12.** Aquaporin gene names and corresponding symbols, model numbers, scaffolds, and arthropod homologues. **Figure S1.** Phylogenetic placement and genomic comparisons for *Stomoxys calcitrans* and other fly species. **Figure S2.** Pearson's correlation of RNA-Seq and RT-qPCR results. **Figure S3.** Average read depth across predicted lateral gene transfer candidates. **Figure S4.** *Stomoxys calcitrans* genomic scaffold housing 11 defensin gene models. **Figure S5.** Maximum likelihood phylogenetic tree of PGRP protein sequences from *S. calcitrans* (red), *M. domestica* (black), and *D. melanogaster* (blue). **Figure S6.** Alignment of *Stomoxys* PGRP-S sequences with characterized *D. melanogaster* PGRP-SC1 (C0HK98) and –SC2 (Q9VX2) and N-acetylmuramoyl-L-alanine amidase (P00806). **Figure S7.** Alignment of *Stomoxys* PGRP-L sequences with characterized *D. melanogaster* PGRP-L proteins and N-acetylmuramoyl-L-alanine amidase. **Figure S8.** *Stomoxys calcitrans* Odorant Binding Protein Gene Family. **Figure S9.** Duplicated OS-E-like and an OS-X ortholog in *Stomoxys* and *Musca*. **Figure S10.** *Stomoxys calcitrans* Odorant Receptor Gene Family. **Figure S11.** *Stomoxys calcitrans* Gustatory Receptor Gene Family. **Figure S12.** *Stomoxys calcitrans* Ionotropic Receptor Gene Family. **Figure S13.** Maximum likelihood tree of dipteran opsin gene relationships. **Figure S14.** Phylogenetic analysis of the Stomoxys Rh1 gene cluster and Genomic organization and evolution of the *S. calcitrans* Rh1 opsin subfamily. **Figure S15.** Analysis of tuning site 17 variation in Stomoxys and Musca Rh1 paralogs. **Figure S16, S18, S19.** Phylogenetic relationship of catalytic carboxyesterases (S16), glutathione-S-transferases (S18), and Cys-Loop Ligand Gated Ion Channels (S19) from *Stomoxys* relative to *Musca* and *Drosophila*. **Figure S17.** Phylogenetic analysis of carboxylesterases with a role in neuronal development. **Figure S20.** Cytochrome P450 (CYP450) genes clustered on *Stomoxys* scaffolds and evidence for expansions in muscids relative to *Drosophila*. **Figure S21.** Phylogenetic analysis of cytochrome P450 genes from *Stomoxys calcitrans*.

**Additional file 2. **Functional annotation of transcripts assembled from the *Stomoxys* genome and output from g:Profiler analysis.

**Additional file 3. **Output from analysis of core CEGMA proteins in the *Stomoxys* genome.

**Additional file 4.** Output from RNA-Seq Dataset Analyses.

**Additional file 5.** Output from cluster analysis of 16S bacterial isolate sequencing.

**Additional file 6. ***Stomoxys* immune-related proteins identified by similarity to HMM profiles of Drosophila immune protein families and by manual annotation.

**Additional file 7. ***Stomoxys* antimicrobial peptide amino acid sequences.

**Additional file 8. ***Stomoxys* chemosensory gene family member amino acid sequences.

**Additional file 9. ***Stomoxys* chemosensory gene family annotation and normalized RNA-Seq expression data.

**Additional file 10. ***Stomoxys* male reproductive tract-biased genes.

**Additional file 11. ***Stomoxys* salivary gland RNA-Seq analysis output.

**Additional file 12. ***Stomoxys* metabolic detoxification gene family member amino acid sequences.

**Additional file 13. ***Stomoxys* cytochrome P450 gene family identification.

**Additional file 14. ***Stomoxys* cytochrome P450 amino acid sequences.

**Additional file 15. ***Stomoxys* transcription factor expression analysis.

**Additional file 16.** Prokaryote and eukaryote databases used for the lateral gene transfer prediction pipeline.

## Data Availability

All whole genome sequence and RNA-Seq transcriptome data are publicly available at the NCBI BioProject database: PRJNA188117 [280], and the reference sequence (RefSeq) genome assembly is available at the NCBI BioProject database: PRJNA288986 [305]. The genome assembly can also be accessed at NCBI accession number GCA_001015335.1. RNA-Seq datasets used in gene prediction have been deposited to the NCBI SRA site under the accession codes SRX995857–5860, SRX229930, SX229931, and SRX275910. Annotation and gene model data are available at VectorBase [283], www.vectorbase.org, *Stomoxys calcitrans* USDA*,* ScalU1.6.
