# Publisher Correction: The genome of the stable fly, *Stomoxys calcitrans*, reveals potential mechanisms underlying reproduction, host interactions, and novel targets for pest control

**DOI:** 10.1186/s12915-021-01098-x

**Published:** 2021-07-29

**Authors:** Pia U. Olafson, Serap Aksoy, Geoffrey M. Attardo, Greta Buckmeier, Xiaoting Chen, Craig J. Coates, Megan Davis, Justin Dykema, Scott J. Emrich, Markus Friedrich, Christopher J. Holmes, Panagiotis Ioannidis, Evan N. Jansen, Emily C. Jennings, Daniel Lawson, Ellen O. Martinson, Gareth L. Maslen, Richard P. Meisel, Terence D. Murphy, Dana Nayduch, David R. Nelson, Kennan J. Oyen, Tyler J. Raszick, José M. C. Ribeiro, Hugh M. Robertson, Andrew J. Rosendale, Timothy B. Sackton, Perot Saelao, Sonja L. Swiger, Sing-Hoi Sze, Aaron M. Tarone, David B. Taylor, Wesley C. Warren, Robert M. Waterhouse, Matthew T. Weirauch, John H. Werren, Richard K. Wilson, Evgeny M. Zdobnov, Joshua B. Benoit

**Affiliations:** 1 Livestock Arthropod Pests Research Unit, USDA-ARS, Kerrville, TX USA; 2 Department of Epidemiology of Microbial Diseases, Yale School of Public Health, New Haven, CT USA; 3 Department of Entomology and Nematology, University of California – Davis, Davis, CA USA; 4 The Center for Autoimmune Genomics and Etiology, Cincinnati Children’s Hospital Medical Center, Cincinnati, OH USA; 5 Department of Entomology, Texas A & M University, College Station, TX USA; 6 Department of Biological Sciences, Wayne State University, Detroit, MI USA; 7 Department of Electrical Engineering & Computer Science, University of Tennessee, Knoxville, TN USA; 8 Department of Biological Sciences, University of Cincinnati, Cincinnati, OH USA; 9 Department of Genetic Medicine and Development, University of Geneva Medical School and Swiss Institute of Bioinformatics, 1211 Geneva, Switzerland; 10 The European Molecular Biology Laboratory, The European Bioinformatics Institute, The Wellcome Genome Campus, Hinxton, CB10 1SD UK; 11 Department of Entomology, University of Georgia, Athens, GA USA; 12 Department of Biology and Biochemistry, University of Houston, Houston, TX USA; 13 National Center for Biotechnology Information, National Library of Medicine, National Institutes of Health, Bethesda, MD USA; 14 Arthropod-borne Animal Diseases Research Unit, USDA-ARS, Manhattan, KS USA; 15 Department of Microbiology, Immunology and Biochemistry, University of Tennessee Health Science Center, Memphis, TN USA; 16 Section of Vector Biology, Laboratory of Malaria and Vector Research, National Institute of Allergy and Infectious Diseases, Rockville, MD USA; 17 Department of Entomology, University of Illinois at Urbana-Champaign, Urbana, IL USA; 18 Department of Biology, Mount St. Joseph University, Cincinnati, OH USA; 19 Informatics Group, Faculty of Arts and Sciences, Harvard University, Cambridge, MA USA; 20 Department of Entomology, Texas A&M AgriLife Research and Extension Center, Stephenville, TX USA; 21 Department of Computer Science & Engineering, Department of Biochemistry & Biophysics, Texas A & M University, College Station, TX USA; 22 Agroecosystem Management Research Unit, USDA-ARS, Lincoln, NE USA; 23 University of Missouri, Bond Life Sciences Center, Columbia, MO USA; 24 Department of Ecology and Evolution, University of Lausanne, and Swiss Institute of Bioinformatics, 1015 Lausanne, Switzerland; 25 Center for Autoimmune Genomics and Etiology, Divisions of Biomedical Informatics and Developmental Biology, Cincinnati Children’s Hospital Medical Center, Cincinnati, OH USA; 26 Department of Pediatrics, University of Cincinnati College of Medicine, Cincinnati, OH USA; 27 Department of Biology, University of Rochester, Rochester, NY USA; 28 Institute for Genomic Medicine, Nationwide Children’s Hospital, Columbus, OH USA; 29 College of Medicine, Ohio State University, Columbus, OH USA

**Correction to: BMC Biol 19, 41 (2021)**

**https://doi.org/10.1186/s12915-021-00975-9**

Following publication of the original article [1], it was reported that the article copyright was incorrect. The correct copyright statement is:

© This is a U.S. Government work and not under copyright protection in the US; foreign copyright protection may apply 2021. 

The original article [1] has been corrected.
