# Supplementary material for: The genome of the stable fly, Stomoxys calcitrans, reveals potential mechanisms underlying reproduction, host interactions, and novel targets for pest control
Source: BMC Biol. 2021 Mar 10;19:41. doi: 10.1186/s12915-021-00975-9 (PMC7944917; doi:10.1186/s12915-021-00975-9)
Supplement: Supplementary file 1 — Additional file 1: Main supplementary text file, including supplementary Tables S1-S12 and supplementary Figures S1-S21; Table S1. RNA-Sequencing and Whole Genome Sequencing Accession Numbers and Statistics. Table S2. Summary of Dfam repeat elements with > 1000 copies including number of unique elements for each family of elements and total number of genomic copies for each family. Table S3. Detailed list of repeat elements with total genomic copy number > 1000. Table S4. Autophagy genes identified from the Stomoxys genome. Table S5. Validation of Stomoxys transcript expression by RT-qPCR. Table S6. Bacterial contaminating scaffolds located in the Stomoxys genome assembly. Table S7. Predicted lateral gene transfer events identified from the Stomoxys genome. Table S8. Components of the immune deficiency, Toll, and JAK/STAT pathways identified from the Stomoxys genome. Table S9. Manually annotated Stomoxys immune system gene family members. Table S10. RNA-Seq normalized expression values for transcripts annotated as antimicrobial peptides. Table S11. Stomoxys opsin gene compilation. Table S12. Aquaporin gene names and corresponding symbols, model numbers, scaffolds, and arthropod homologues. Figure S1. Phylogenetic placement and genomic comparisons for Stomoxys calcitrans and other fly species. Figure S2. Pearson's correlation of RNA-Seq and RT-qPCR results. Figure S3. Average read depth across predicted lateral gene transfer candidates. Figure S4. Stomoxys calcitrans genomic scaffold housing 11 defensin gene models. Figure S5. Maximum likelihood phylogenetic tree of PGRP protein sequences from S. calcitrans (red), M. domestica (black), and D. melanogaster (blue). Figure S6. Alignment of Stomoxys PGRP-S sequences with characterized D. melanogaster PGRP-SC1 (C0HK98) and –SC2 (Q9VX2) and N-acetylmuramoyl-L-alanine amidase (P00806). Figure S7. Alignment of Stomoxys PGRP-L sequences with characterized D. melanogaster PGRP-L proteins and N-acetylmuramoyl-L-alanine amidase. [file 12915_2021_975_MOESM1_ESM.docx]

**Additional File 1:**

***Stomoxys calcitrans* Genome Sequencing Project**

**SUPPLEMENTARY INFORMATION**

**BUSCO Assessment**

**Contributors:** Joshua B. Benoit (University of Cincinnati), Ellen O. Martinson (University of Rochester)

*Benchmarking Universal Single-Copy Orthologs (BUSCO) and OrthoFinder analyses*

We used BUSCO to assess quality of the genome assembly and completeness of the predicted gene set. Based on the near-universal single-copy orthologs from dipterans (OrthoDB v8, [1]), 95.1% were found in the assembly and 92.2 in the final predicted gene set.

A species phylogeny based on 664 protein sequences (269,392 amino acids) was reconstructed to determine the evolutionary relationships among nine dipteran species (Fig. S1). The official protein set of *Sarcophaga* *bullata*, *Lucilia* *cuprina*, *Musca* *domestica*, *Glossina* *morsitans*, *Drosophila* *melanogaster*, *Mayetiola* *destructor*, *Aedes* *aegypti*, and *Anopheles* *gambiae* were downloaded from NCBI and searched against the *S*. *calcitrans* gene set using BLASTp. A significant e-value cut-off ≤1e-5 was applied and only genes that had a single hit across all eight species were included in further analysis. A total of 664 individual proteins were aligned with MAFFT [2] using default settings, and alignments were trimmed using gBlocks to remove gaps [3]. The aligned single-copy protein-coding genes were then concatenated and the phylogeny was reconstructed using RAxML version 8.2.8 [4] with the PROTGAMMAWAG model and 100 bootstrap replicates. The phylogeny was visualized with FigTree version 1.4.2 (http://tree.bio.ed.ac.uk/software/figtree/). Orthologous groups of genes were also determined among the nine species using OrthoFinder (v 2.2.7) [5] using default settings.

**Stable Fly Chromosome Arm Assignments**

**Contributor**: Richard P. Meisel (University of Houston)

***Assigning autosomes to Muller elements***

Fly genomes are organized into six chromosome arms, known as Muller elements [6, 7]. Elements A–E correspond to the five gene-rich chromosomes that are autosomal in the most-recent common ancestor, and element F is a gene-poor heterochromatic X chromosome in the common ancestor [8]. The *S.calcitrans* karyotype is missing the heterochromatic element F [9-11], suggesting that it fused to one of the other elements. We have identified the chromosome fused to element F in a companion paper, which also reports the identity of the stable fly sex chromosomes [12]. Here we report the reconciliation of the traditional stable fly chromosome numbering scheme with the Muller element nomenclature. Determining relationships between *S. calcitrans* chromosome numbers and the Muller elements is difficult because of a paucity of mutant markers that have been mapped to *S. calcitrans* autosomes. A mutation causing a black puparium (*bp*) was mapped to *S. calcitrans* chromosome 3 [13]. A similar mutation was mapped to house fly (*M. domestica*) chromosome 1 and sheep blowfly (*Lucilia cuprina*) chromosome 2, both corresponding to Muller element B [14, 15]. It is therefore reasonable to hypothesize that *S. calcitrans* chromosome 3 corresponds to Muller element B. The *S. calcitrans* rolled down wing (*rd*) mutation [16] is phenotypically similar to the house fly *ali-curved* mutation [17, 18], which also maps to house fly chromosome 1 (Muller element B). However, *rd* was mapped to *S. calcitrans* chromosome 4 [16], and not chromosome 3 as expected based on the location of *bp*. The caramine eye (*ca*) and malathion resistance (*malR*) mutations both map to *S. calcitrans* chromosome 2 [16, 19], and they have multiple possible orthologs in other fly species on Muller elements C and E [15, 20]. We therefore conclude that Muller element B either corresponds to *S. calcitrans* chromosome 3 or 4, and *S. calcitrans* chromosome 2 therefore either corresponds to Muller elements C or E.

**Methods**

***Chromosome mapping.*** We used 1:1 orthologs between *D. melanogaster* and *S. calcitrans* to map genes and scaffolds to chromosome arms (Muller elements). This approach works because chromosome arm gene content (synteny) is conserved across higher dipterans [6, 15], and we have previously demonstrated its efficacy with the house fly genome [21].

**Presence and enrichment of repeat elements in *Stomoxys calcitrans* genome**

**Contributors:** T. J. Raszick, S.-H. Sze, C. J. Coates, A. M. Tarone (Texas A&M University)

**Materials and Methods**

A hidden Markov model (HMM)-based approach was used to identify repeat elements in the *S. calcitrans* genome including Class I and II transposons, non-coding RNAs, and satellite sequences (Table S2). The database of HMMs for 4150 unique repeat elements was obtained from Dfam [22] and evaluated against the *S. calcitrans* genome assembly using nhmmer [23]. Repeat elements identified as having at least one copy in the genome were then further investigated with regards to repeat sequence enrichment for each putative Muller element (Table S3) using a Bonferroni-corrected hypergeometric test (α = 0.05/1165 = 4.291845e-05). Repeat elements occurring on contigs that were not assigned to any Muller element were also included in this analysis.

For all significantly enriched repeat elements with copy number >1000 found in association with a Muller element, DNA sequences for up to 25 copies were randomly subsampled from each Muller element where those elements occurred. Also, a custom BLAST database was created with the Dfam consensus sequences for those repeat elements. The random subset of sequences was then locally compared to the custom database in a reciprocal BLAST designed to ensure that the results of the HMM-based approach were consistent with the consensus sequences available from Dfam.

**Results**

The HMM-based search for repeat elements yielded a total of 1165 unique Dfam repeat elements with at least one copy in the *S. calcitrans* genome assembly. Of these, 50 occurred frequently, with >1000 copies genome-wide. The majority of those 50 were Class II transposons, but Class I transposons were also common (Table S3). Cut and paste transposons displayed the highest diversity of unique elements, and rolling circle transposons, while less diverse, occurred with the highest copy number. DNAREP1 was the most common across all types of repeat elements, accounting for 90,273 of the 112,877 occurrences of rolling circle transposons. A subset of 15 unique repeat elements with total genomic copy number >1000 were found to be significantly enriched in association with at least one Muller element (Table S3). Our reciprocal BLAST search reassigned these repeat elements to appropriate consensus sequences with 76.22% success (1010 best hits to the exact same element or to a similar element out of 1325 subsampled sequences).

The Muller D and F elements had distinct patterns of enrichment when contrasted with each other or with Muller A, B, C, and E elements. There were nine unique repeat elements that were significantly enriched in association with one or more of the Muller A, B, C, and E elements, and none of those nine were significantly enriched in association with the D or F. Conversely, there were three unique repeat elements that were significantly enriched in association with the Muller D element and no other Muller element. This pattern suggests that there is some distinct feature of the Muller D element that alters the way it interacts with repeat elements. No repeat elements were significantly enriched in association with the Muller F element. There were three repeat elements that were found to be significantly enriched in association with contigs that were not assigned to any Muller element in the assembly.

**Immune System**

**Contributors**: Tim Sackton (Harvard University), Pia Olafson (USDA-ARS), Dana Nayduch (USDA-ARS)

In addition to our genome-wide computational annotation of immune genes, we focused our manual annotation efforts on antimicrobial peptides (AMPs), which can be challenging for *in silico* annotation algorithms to detect due to their short size (Table S9). We focus on four classes of AMPs: defensins (DEF), attacins (ATT), diptericins (DIPT), and cecropins (CEC), all well-characterized and broadly distributed AMPs among insects. Several of the AMP gene families were found clustered on individual scaffolds, possibly arising from tandem duplications. The 11 *Stomoxys* defensin genes are located on a single scaffold (Fig. S3). Four of the five computationally predicted defensins in the *S. calcitrans* genome are clustered on a single scaffold (KQ079966), and manual examination of that region of the genome reveals an additional six defensin gene models that were missed by the computational screen, including one previously reported (Stomoxys midgut defensin (Smd) 1, [24]). There is a 3.3kb gap in the KQ079966 assembly, and primers designed to amplify across the assembly gap confirmed that a smaller scaffold (LDNW01121039) housing a single defensin gene model resides in this gap (Table S9). Thus we believe that the *S. calcitrans* genome likely encodes at least 11 total defensins. The 12 predicted Attacins are largely clustered on four scaffolds [KQ080058 (5), KQ080155 (1), KQ080308 (4), and KQ082105 (2)]; one gene model (LOC106086445) was split to produce two better representative models. Of the three predicted diptericins, likely only one (LOC106086283) is in fact an antimicrobial peptide: while LOC106086281 does encode an ATTC domain, the size and gene structure (16 introns) is not consistent with other insect diptericins and no conserved domains are detectable in LOC106086726. Computation annotation recovers five cecropin-like sequences on a single scaffold (KQ080281). Three additional cecropin gene models were identified on this scaffold, along with two additional cecropin-like gene models with similarity to Stomoxyn [25] on scaffold KQ080227 (Table S7), for a total of 10 cecropins in the *S. calcitrans* genome. Finally, we find a cluster of 9 gene models on scaffold KQ079975 that were *in silico* annotated as lncRNAs, but there is ample RNA-Seq support to indicate they encode short peptides with low sequence similarity to cecropin; these encode predicted signal peptides and alpha-helixes at the C terminus. In the absence of proteomic evidence it is difficult to know for sure whether these sequences are indeed translated into AMP-like peptides, but it seems likely that this cluster represents another set of cecropin-like AMPs in the *S. calcitrans* genome.

To better understand the biology of PGRPs in *S. calcitrans*, we examined patterns of gene expression from whole genome RNA-Seq assays of larvae and adults. We identified 17 PGRPs in the *S. calcitrans* genome (11 in the short subfamily, 5 in the long subfamily, and 1 ambiguous; Fig. S5). Of the members of the short subfamily, six – all homologs of the PGRP-SC gene family in *D. melanogaster* – appear to be only expressed in larvae (XP_013110193, XP_013110191, XP_013106717, XP_013106719, XP_013109922, and XP_013097596; Fig. S6), and based on sequence properties and conservation of residues required for amidase activity [26] are predicted to be both secreted and catalytic (Fig. S6). Interestingly, PGRP-SC genes in both *D. melanogaster* [27] and *M. domestica* [28] are also expressed in larvae where they likely have a role in modulating activation of the IMD pathway. Larval expression is not exclusive in *D. melanogaster*, though, suggesting the possibility that larval-specific expression may be a *S. calcitrans* innovation.

The remaining five members of the short family have more variable expression patterns, as well as variable evidence for amidase activity. The *Stomoxys* PGRP-SB ortholog (XP_013114626) likely retains catalytic activity, while the *Stomoxys* PGRP-SA (XP_013098814, XP_013098815, and XP_013098810) and –SD (XP_013108408) orthologs likely lack catalytic activity but may serve a receptor function [26]. The five members of the long subfamily, in contrast, all appear to have relatively broad expression patterns and variable evidence for amidase activity (Fig. S7). The PGRP-LB ortholog (XP_013109943) encodes a predicted signal peptide and conserved residues supporting peptidoglycan binding and amidase activity, which is in keeping with the characterized *Drosophila* PGRP-LB that is regulated by the IMD pathway and cleaves peptidoglycan of gram negative bacteria [29]. The *Stomoxys* PGRP-LE (XP_013111331, XP_013111330) orthologs were identified at two loci tandemly arranged in opposite directions, and these are predicted to bind peptidoglycan but are likely non-catalytic, which has been demonstrated for *Drosophila* PGRP-LE [30]. The PGRP-LA ortholog (XP_013109991) does not encode residues associated with peptidoglycan binding, but may have a regulatory role [31], and the ambiguous gene (XP_013113924) encodes a PGRP-LD like polypeptide that is only very weakly expressed in any stage based on RNA-Seq data; the predicted sequence does not support peptidoglycan binding. The expanded PGRPs in *S. calitrans*, then, are from the short subfamily, especially the SC-like members, and tend to have larval-specific expression patterns.

Overall, our analysis of the *S. calcitrans* immune system as predicted by its genome reveals a dynamic system that recapitulates many of the trends first noted in *M. domestica*, suggesting that expanded repertoires of recognition and effector proteins may be a general feature of muscid fly genomes.

***Stomoxys* Chemosensory Gene Families (Full Reports)**

**Contributors:** Hugh Robertson (University of Illinois at Urbana-Champaign), Pia Olafson (USDA-ARS)

***Stomoxys calcitrans* gustatory receptors**

The Gustatory Receptor family is the more ancient of the two families that make up the insect chemoreceptor superfamily [32-35], and comprises several highly divergent lineages, most involved in taste but some in olfaction [36]. The Odorant Receptor family arose from a GR lineage near to or within a basal insect lineage [37, 38]. In *Drosophila melanogaster* the GR family consists of 60 genes encoding 68 proteins through an unusual form of alternative splicing where multiple long first exons are spliced into a shared single or set of exons encoding the conserved C-terminus of the protein. In *Musca domestica*, the family consists of 80 genes encoding 108 proteins ([39]– 77 genes encoding 101 proteins were reported therein, but as described below, three more genes encoding an additional 7 proteins are recognized here). The family is generally divided into three major and divergent subfamilies. The sugar or sweet receptors, the carbon dioxide receptors, and the bitter taste receptors, but with complications, for example, a lineage within the bitter taste receptor clade has evolved into an important receptor for fructose [40], while others are involved in courtship [41].

The ScalGr gene set consists of 74 models, encoding 113 potential proteins through alternative splicing of eight loci (Additional file 8). Ten (9%) of these genes or isoforms are apparent pseudogenes, regions of seven models were repaired using raw genomic and/or RNAseq reads, five models were joined across scaffolds, and eight remain incomplete. Eight genes are modeled as being alternatively spliced and occasionally RNAseq evidence supporting these alternative splices was available. The resultant isoform proteins often differ considerably in most of their sequence, and hence presumably bind different ligands. They are indicated with a lower case letter after the gene name (not to be confused with the lower case letter after the Gr names in Drosophila, e.g. Gr2a, which instead indicates the cytological location of the gene, and hence to avoid confusion alternatively-spliced isoforms in Drosophila are indicated with an upper case letter, e.g. Gr39aC). As a result, the number of apparently intact GR proteins is 103. Less obvious pseudogenes (for example with small in-frame deletions or insertions, crucial amino acid changes, or promoter defects) would not be recognized, so this total might be high.

The GR repertoire was primarily compared with that of *M. domestica* [39], which is in the same family of flies, and *D. melanogaster* [34], as the functions of some of these proteins are at least partially known from the latter. Additional phylogenetic analysis with a more distantly related fly with a complete genome sequence and chemoreceptor analysis, the medfly *C. capitata* [42], was undertaken and is mentioned when relevant, but is not shown. Details of the major subfamilies and gene lineages are below, along with a heat map of normalized expression values for annotated *Gr* transcripts (Fig. S11; Additional File 9).

*Stomoxys* has the same set of carbon dioxide receptors as *Musca*, with a duplication of the Gr1 lineage (DmelGr21a) in inverted orientation like *Musca*. These two genes are somewhat awkwardly named Gr1.1 and 1.2, as was done in *Musca*, to maintain a proposed convention that the carbon dioxide receptors be named Gr1-3 [43]. The absence of the Gr2 lineage (also absent from Drosophila) helps confirm that this loss occurred before the Muscidae and Drosophilidae split, but after they separated from the Tephritidae because *Ceratitis* has it.

*Stomoxys* has the same set of eight sugar receptors as *Musca* (Gr4-11). In *Musca* these genes were generally poorly assembled on mostly separate scaffolds, and so both their phylogenetic relationships to the *Drosophila* sugar receptors and their relative locations in the genome were unclear. In *Stomoxys* the situation is somewhat better in that most of these genes are full-length or the assembly could be repaired to be full-length, and Gr7-10 are in an array crossing two scaffolds, however Gr4-6 and 11 are all in separate scaffolds. It therefore remains unclear when these genes became separated as they are in *Drosophila* with the first and last genes (Gr61a and 5a) having moved from the contiguous tandem array in *Ceratitis* that is hypothesized to be the ancestral arrangement in flies [43]. Their phylogenetic relationships are also clarified, with the muscids having duplicated the DmelGr64b lineage (as Gr7/8), while *Drosophila* duplicated DmelGr64c/d. Gr7 is an apparent pseudogene in *Stomoyxs*, with a stop codon near the start codon, confirmed by multiple RNAseq and genomic reads, however as with all such young pseudogenes, this nonsense mutation might be unique to the sequenced strain.

*Stomoxys* has the same duplication of the highly conserved DmelGr43a lineage of fructose receptors as *Musca* (Gr12/13), a lineage independently duplicated to four genes in *Ceratitis* (CcapGr11-14). This lineage clusters with several well-known bitter taste receptors, e.g. DmelGr66a and 33a, and hence presumably evolved from a receptor that originally detected a bitter compound. It certainly did not evolve from within the sugar receptor subfamily.

Most of the remaining *Drosophila* GRs are implicated in perception of bitter tastants or have not yet been functionally characterized [44-57]. The *Stomoxys* representatives were named roughly in the order of the *Musca* genes, however because of gene duplications and losses, this naming system eventually becomes disconnected with orthology. In addition, some of these are rapidly evolving genes, so establishing orthology is not always clearcut, and when feasible microsynteny analysis was employed to assist with these decisions. They are described below in the order of their naming, in paragraphs corresponding to major lineages in the tree, with details of known functions, many of which are relatively new discoveries in *Drosophila*. In particular, the work of Delventhal and Carlson [46] has revealed ligand specificities for some of these bitter taste receptors, showing the breadth of their ability to mediate sensing of diverse bitter compounds.

Gr14 is the conserved ortholog of DmelGr32a, which is a well-known bitter receptor and one of five expressed in all four types of bitter taste neurons on the labellum. It is also involved in courtship through expression in a small set of gustatory receptor neurons on the male foreleg [58], and is implicated in inhibition of male-male courtship [54, 59], as well as mediating rejection of non-conspecific females as targets of male courtship [41], and mediating aggression [60]. DmelGr68a is a relative of Gr32a also involved in courtship [61-63], but it was lost from both *Musca* and *Stomoxys*. Gr15 is a complicated locus with an interesting history in these flies, and is also the next relative of Gr32a/68a and is likely to be involved in courtship [64]. *Ceratitis* has a single gene, Gr20. In *Stomoxys* this locus has two alternatively-spliced isoforms (Gr15a/b). In *Musca* each of these isoforms has been independently duplicated (Gr15a/b and c/d), and remarkably these two isoforms were again independently duplicated in *Drosophila* where they are known as Gr39aA/B and C/D, and the locus has undergone additional duplications in other Drosophila species [65]. DmelGr39aA is another of the five receptors expressed in all four bitter taste neurons in the labellum [64]. Unfortunately when annotating the *Musca* genome, two additional alternatively-spliced loci related to these were missed. They are therefore numbered after the current final *Musca* Gr, so are MdomGr77a-d and 78a/b. In *Stomoxys* they are Gr71a-e and 72 (*Stomoxys* has an additional alternatively-spliced first exon in Gr71, while *Musca* has duplicated the first exon of Gr78). The phylogenetic relationships of these two new genes, which are neighbors in each genome, are unusual. They appear to be confidently related to the DmelGr39aC/D, MdomGr15c/d, and ScalGr15b lineage, implying that they are a duplicate from the terminal alternatively-spliced part of this locus. This relationship is also supported in smaller trees using only the proteins described in this paragraph. *Ceratitis* does not have these genes. It appears then that *Stomoxys* has a slightly smaller repertoire of these GRs involved in the social behaviors of courtship and aggression than does *Musca*, but both have a set of GRs absent from *Drosophila*, while having lost the DmelGr68a ortholog.

ScalGr16-18 are together as neighbors in one scaffold, as is the case for their *Musca* relatives. *Drosophila* lost the ortholog of Gr17 (which is a frameshifted pseudogene in *Stomoxys*), but Gr16 is the ortholog of DmelGr2a while Gr18 is related to DmelGr23aA/B. Both of these genes in *Drosophila* are expressed in larval pharyngeal organs [45] and the adult labral sense organ and Gr2a is required for avoidance of high salt concentrations in food [66]. Delventhal and Carlson [46] find that Gr2a responds to several plant bitter compounds including the alkaloids caffeine, lobeline, and theophylline, as well as the quite dissimilar phenylpropanoid umbelliferone. Like DmelGr23aA/B, the Gr18 locus is also alternatively-spliced in *Stomoxys*, however this is an ancient duplication of the first exon, independent of the DmelGr23aA/B duplication, and *Musca* has lost the second isoform from its version of the Gr18 locus, so only has a single isoform related to ScalGr18a. Clustering with Gr17/18 in the tree is MdomGr19, which is the highly diverged ortholog of DmelGr39b (supported by microsynteny analysis as well). But *Stomoxys*, despite maintaining microsynteny of the flanking genes, has lost this gene. MdomGr20/21 are thought to be duplicates distantly related to DmelGr98a, although they do not cluster in the tree but do when CcapGr37 is included in the analysis, but this gene lineage was also lost from *Stomoxys*. *Stomoxys* has a single ortholog of DmelGr8a (ScalGr19), a receptor for a plant-derived insecticide L-canavanine [48, 67], but this lineage expanded to 7 genes in *Musca* (MdomGr22-28). Related to this lineage is the alternatively-spliced gene MdomGr29a-c, which has a relative in *Ceratitis* (CcapGr33), but was independently lost from *Drosophila* and *Stomoxys*. DmelGr98b-d are one of the few examples of a small tandem array of GR genes in *Drosophila*, and are related to an independent expansion of six genes, some in an array, in *Musca* (MdomGr30-35). *Stomoxys* has only two genes in this lineage (ScalGr20 and 21) quite separate in two large scaffolds, and their phylogenetic relationships suggest that they are separately related to the expansions of MdomGr30-32 and 33-35, respectively, and that some of these duplications occurred early in the *Muscid* lineage and were lost from *Stomoxys*. DmelGr98b is also involved in perception of L-canavanine [67], so this set of muscid expansions is likely involved in perception of similar bitter compounds. There is a single ortholog for DmelGr9a in *Musca* (MdomGr41), but it is an alternatively-spliced gene in *Stomoxys* (ScalGr27a/b) (this relationship is not reflected in the tree, but is found in larger trees including *Ceratitis*, and microsynteny analysis supports their orthology). Finally, at the base of the cluster of the above GR lineages in the tree are MdomGr76 and ScalGr70, a lineage with a single conserved ortholog in *Ceratitis* (CcapGr70), but which was lost from *Drosophila*. This phylogenetic cluster of bitter taste receptors has been extensively studied in *Drosophila*, where they are variously expressed in the labellum, abdominal neurons, and forelegs of adult flies, as well as in larvae and several are known to mediate perception of important plant bitter compounds. *Stomoxys* has just 10 proteins in this phylogenetic cluster, the same number as *Drosophila*, compared with 24 in *Musca*, resulting from gene losses in *Stomoxys* and duplications in *Musca*, suggesting that *Musca* has a greater chemical ecological need for this clade of bitter receptors.

ScalGr22 is the ortholog of MdomGr36 and DmelGr66a, one of the best-known bitter taste receptors and another of the five expressed in all four classes of bitter taste neurons on the labium. Like *Musca* (MdomGr37), *Stomoxys* has a paralog (ScalGr23) that is absent from *Drosophila*, so presumably this MdomGr37/ScalGr23 lineage, which is also present in *Ceratitis* (CcapGr39), is another ancient bitter receptor that *Drosophila* lost. ScalGr24 is the ortholog of MdomGr38 and DmelGr33a, another well-known bitter receptor and another of the five expressed in all four types of bitter taste neurons on the labium. Related to it is a complicated alternatively-spliced gene, ScalGr25a-h, MdomGr39a-g, and DmelGr28bA-E. In *Drosophila* the isoforms from this gene are involved in bitter taste as well as being expressed in other neurons [46, 68], and some have been implicated in sensing light and temperature, e.g. [69-71]. The muscid flies have orthologous isoforms for each of the five *Drosophila* isoforms, but some have been additionally duplicated in the muscids. All are highly conserved in sequence, and may well perform similar roles in muscids as in *Drosophila*. Immediately downstream of this complex gene in each genome is the similarly well-conserved receptor, ScalGr26, MdomGr40, and DmelGr28a, which has similar broad expression in both gustatory and other neurons [68] and responds to a wide variety of bitter compounds [46]. Note again that the fructose receptor DmelGr43a and its muscid orthologs cluster with these bitter receptors, having presumably changed its ligand from a bitter tastant to fructose at some point.

ScalGr28 is the ortholog of MdomGr42 and DmelGr10a, another well-known bitter receptor that has a remarkably similar response profile to a quite of bitter compounds as Gr2a, despite being phylogenetically far removed from it [46]. In *Stomoxys* this gene is at the start of a large and complicated array of genes spanning two large scaffolds, with additional related genes on four other large scaffolds implying that they moved from the original array of genes, for a total of 48 proteins (ScalGr29-57) including alternative splicing of Gr47a-t. These form a major expansion of candidate bitter taste receptors in *Stomoxys*, comparable to a similarly complicated set in *Musca* (MdomGr43-64, encoding 35 proteins), and together these are a major expansion compared with *Drosophila* and *Ceratitis*. In *Ceratitis* and *Musca* these genes are all adjacent to each other in a single scaffold, which indicates how an original expansion in a fly ancestor has become hugely expanded in the muscids, but not *Drosophila*, while in the latter the genes have been split up on different chromosome arms. The presence of subsets of these genes in four other large scaffolds in *Stomoxys* (Gr48/49, 50, 51-54, and 55-57) implies that some genomic shuffling has occurred on this fly lineage as well. The tree reveals that these genes form three major expanded clades in the muscids. CladeA is related to DmelGr59a/b and consists of independent expansions in the two muscids, with two genes encoding five proteins in *Musca* versus 23 in *Stomoxys*. Clade B has four genes in *Stomoxys* and 16 in *Musca*, all of which are *Musca*-specific duplicates. The relationship of this clade to the *Drosophila* GRs in the tree is unclear. Clade C is related to DmelGr36a-c and Gr59c/d and consists of a simple gene (ScalGr46 and MdomGr51) adjacent to a large alternatively-spliced gene in each species (ScalGr47a-t and MdomGr52a-k). Most of the duplications of these first exons occurred within either *Musca* or *Stomoxys*, presumably from a much smaller ancestral alternatively-spliced locus. In *Stomoxys* this large gene spans two large scaffolds and one contig. In *Drosophila*, Gr59c responds to the plant alkaloids berberine and lobelline, while Gr36a responds to a wide variety of bitter compounds including the alkaloid sparteine, the terpenoid saponin, and the phenanthrene aristocholic acid [46]. Finally, one member of this expansion in *Musca* (MdomGr43) is so divergent it does not cluster with the others, and similarly several *Drosophila* GRs that might be related to these expansions do not cluster confidently with them, e.g. DmelGr10b, 47a, and 85a. In larger analyses including the *Ceratitis* GRs, DmelGr10b clusters with MdomGr43, while DmelGr47a and 85a cluster with Clade B. DmelGr47a is a narrowly-specific receptor for the plant bitter compound strychnine [50]. Although neither of the muscids appears to have a convincing close relative for the recently-duplicated DmelGr22a-f genes in the tree, *Ceratitis* has six related and also recently-duplicated genes, and in larger trees including *Ceratitis*, ScalGr60a/b and MdomGr67 cluster with this lineage. In *Drosophila*, Gr22b responds to several bitter compounds from plants like the glycoside cucurbitacin, the terpenoid azadirachtin, and the alkaloids lobeline and berberine [46]. In summary, this large grouping of genes, originating from a small cluster in a fly ancestor and now distributed across the *Drosophila* genome, has been expanded in a major fashion in the muscids, and especially in *Stomoxys*, with a total of 49 proteins versus 36 in *Musca*, 17 in *Drosophila*, and 12 in *Ceratitis*.

ScalGr58 and 59 are simple orthologs of MdomGr65 and 66 and DmelGr47b and 57a, respectively, forming a divergent lineage in the tree. In Drosophila Gr57a is expressed along with 2a, 23a, and 93d in the larval pharyngeal organ [45] and the adult labral sense organ [66], but nothing is known about their ligand specificities.

ScalGr60-62 are together in one scaffold and related to MdomGr67-69 and DmelGr58a-c, however two of them are alternatively-spliced encoding nine proteins and constituting yet another Stomoxys-specific expansion of candidate bitter taste receptors. As noted above, ScalGr60a/b and MdomGr67 might be related to DmelGr22a-f. ScalGr61 and the alternatively-spliced Gr62a-f are related to MdomGr68 and DmelGr58a/b in another expanded Clade D, while *Stomoxys* appears to have lost the ortholog of MdomGr69/DmelGr58c. Gr58c was included in the study by Delventhal and Carlson [46] and responds to an overlapping set of bitter compounds as Gr22b, but also to additional plant bitter compounds such as the flavonoid myricetin, the benzopyrone coumarin, and the alkaloids strychnine and quinine. Presumably then this is yet another expansion of bitter receptors in *Stomoxys*.

ScalGr63-65 are together in a scaffold and related to MdomGr70/71 and DmelGr59e/f in another distinctive gene lineage, but little is known about Gr59e/f beyond expression in the larval sense organs [47].

ScalGr66-69 are rapidly evolving orthologs of several more *Musca* and *Drosophila* Grs, all clustering weakly phylogenetically with the large expansions described above. ScalGr66 is the ortholog of MdomGr72 and DmelGr77a, ScalGr67 is the ortholog of MdomGr73 and DmelGr89a (the fifth Gr expressed in all four bitter neuron types), ScalGr68 is the ortholog of MdomGr74 and DmelGr93a, which is required for response to caffeine [49], while the alternatively-spliced ScalGr69a/b is the ortholog of MdomGr75a/b and DmelGr94a and related to Gr97a, which along with Gr33a and 66a is involved in quinine perception [44]. This cluster includes a previously unrecognized gene in *Musca*, which is named MdomGr79, with ortholog ScalGr73, related to DmelGr92a and 93b-d, the last of which genes is expressed along with 2a, 23a, and 57a in the larval pharyngeal organ [45] and the adult labral sense organ [66].

In summary, while the carbon dioxide, sugar, and fructose receptors are relatively well conserved in these two muscids, as is the case for many other insects, the bitter taste receptors reveal considerable gene family evolution both with respect to the available relatives of these muscid flies, *Drosophila* and *Ceratitis*, and between these two muscids. *Stomoxys* has lost or not expanded several lineages implicated in courtship, but expanded several others. The ligands for some of these *Drosophila* bitter receptors are being elucidated [46] and provide an early glimpse into what these expansions might mean for the sensory capabilities of these muscids, however it will require determination of the ligand specificities of these muscid receptors to fully understand the ecological significance of the differential expansions and contractions of their bitter taste abilities.

***Stomoxys* Ionotropic Receptor (IR) Gene Family**

The Ionotropic Receptor family is a variant lineage of the ancient ionotropic glutamate receptor family [32, 72-74]. These proteins have three transmembrane domains. Like the GRs they are involved in both olfaction and gustation, as well as sensing light, temperature, and humidity [74]. In *D. melanogaster* the family consists of 60 intact genes and four long pseudogenes. *M. domestica* has a considerable expansion to 100 intact genes and nine pseudogenes [39].

The IR family consists of 131 intact genes and 14 pseudogenes for a total of 145 models (Additional file 8), although nine remain partial with termini missing in gaps, while three were repaired and one was joined across scaffolds. The IRs were named using the same convention as employed for *Musca*, *Ceratitis*, and several other arthropods, with those showing clearcut orthology with *Drosophila* IRs named for their *Drosophila* orthologs, after which genes were named in a series from Ir101, to avoid any confusion with the *Drosophila* IRs, which only go up to Ir100a, having been named for their cytological locations. Details of the major subfamilies and gene lineages are below.

In *Drosophila* and most other insects examined to date, there are two IRs that are highly conserved both in sequence and length and in being phylogenetically most closely related to the ionotropic glutamate receptors from which this variant ionotropic receptor family of chemoreceptors evolved [72, 73]. These are Ir8a and 25a, both of which function as co-receptors with other IRs [74]. While *Stomoxys* has the expected single conserved ortholog of Ir8a, surprisingly it has four paralogs of Ir25a, named Ir25a1-4. The first exon is missing from the assembly for two of these, apparently in gaps, but otherwise they all appear intact and all but Ir25a2 have extensive RNAseq support. They therefore appear to be functional genes/proteins, but their functions are enigmatic as such duplications of Ir25a are rarely observed in other insects.

Most of the remaining conserved IRs with convincing orthologous relationships are indicated with their *Drosophila* names in Fig. S12. For the most part these are simple 1:1 orthologous relationships, and include proteins such as Ir76b, a third apparent co-receptor involved in sensing salt, amines, and amino acids [75-78], Ir41a implicated in perception of polyamines [77], Ir64a implicated in perception of acidic odors [79, 80], Ir84a which has phenylacetic acid and phenylacetaldehyde as ligands [81], Ir75a that mediates perception of acetic acid [Prieto-Godino, 2017 #115][82], Ir75b/c that respond to butyric and proprionic acid in *D. melanogaster* and hexanoic acid in *D. sechellia* [83], and Ir21a, 40a, 68a, and 93a implicated in temperature and humidity sensing [84-87]. Several instances of duplications in *Stomoxys* are evident, specifically Ir10a1/2, Ir41a1/2, and Ir75d1/2. The latter two lineages are commonly expanded in other insects, and in *Drosophila* are involved in sensing various acids and amines [77, 82, 85]. In *Musca*, in contrast, there are five duplicates of Ir10a (two are pseudogenes) and three duplicates of Ir76a, but the ligands are unknown for these two genes in *Drosophila*.

The Ir7a-g and 11a genes in *Drosophila* are expressed in larval and adult gustatory organs [85], but ligands for these receptors are unknown. This subfamily is considerably expanded in the muscids, and given the complexities of the relationships, in both *Musca* and *Stomoxys* they are not named for their *Drosophila* relatives, but rather begin the numbered series from Ir101, in the case of *Stomoxys* to Ir121 and in *Musca* to Ir126 (Fig. S12). These IR gene expansions strongly suggest an expanded gustatory capacity.

Finally, a large clade of “divergent” IRs in *Drosophila* is involved in gustation and is known as the Ir20a subfamily of 33 proteins (including the four pseudogenic ones) [88, 89]. This clade of mostly intronless genes is considerably expanded in *Musca* to 53 members (MdomIr127-179), and even more so in *Stomoxys* to 96 members (ScalIr122-217). This subfamily consists mostly of minor expansions in *Drosophila* and major expansions in the two muscids, labeled clades A-G in the tree (Fig. S12). Although support for these clades is low, they do appear to represent seven independent expansions of lineages that are represented by 0-6 genes in *Drosophila*. The largest of these is clade G consisting of 16 *Musca* genes (MdomIr163-178) and 50 genes in *Stomoxys* (ScalIr153-202), apparently related to DmelIr47a/b, 67a, and 94a-c. Also noteworthy is clade B, which has just one *Musca* gene (MdomIr149), but 15 *Stomoxys* genes (ScalGr203-217), apparently related to DmelIr60e and 67b/c. Putative ligands in *Drosophila* are limited to carbonation sensing and specific carbohydrates [90, 91], but the expansion in *Stomoxys* warrants study as feeding preference on carbohydrates is low. Expression pattern revealed one specific IR (ScalIR119) that is highly enriched in the male RS compared to all other stages (Fig. S12, Additional File 9). Of interest, the ortholog for this IR in *Drosophila* (Ir7a) has increased expression associated with both male and female reproductive organs [92], suggesting a potential critical role in fly reproduction. This Ir20a subfamily also has most of the pseudogenes, although the Ir7 subfamily also has some, indicating that these two subfamilies, in addition to numerous young duplicates in the muscids, also exhibits the most gene losses, and hence are the most evolutionarily dynamic parts of the IR family. The expansions of this and the Ir7 subfamily in the muscids, and particularly *Stomoxys*, mirror those of bitter taste receptors in the GR family.

***Stomoxys* Odorant Binding Protein (OBP) Gene Family**

Ninety OBP gene models were identified in the *Stomoxys* genome (Additional File 8). ScalObp50/MdomObp51 is an OS-E-like protein, as it clusters with DmelObp83b (OS-E), which is co-expressed along with DmelObp83a (OS-F) and a third OBP (DmelObp76a) in a subset of *Drosophila* sensilla (trichoid). ScalObp48/MdomObp49 and ScalObp49/MdomObp50 are arranged in tandem on scaffold KQ081519, and they encode OS-E/-F-like proteins. Smaller trees constructed using related OS-E/-F proteins from a variety of *Drosophila* species supported an absence of an *OS-F* encoding gene in *Stomoxys*; rather, ScalObp49/MdomObp50 appears to be related to the newly identified OS-X proteins (Fig. S9; [93]). All three *Stomoxys* sequences have conserved intron/exon boundary locations and ScalObp50, while arranged at the terminal end of a separate scaffold (KQ081571), is likely part of the same chromosome.

***Stomoxys* Odorant Receptor (OR) Gene Family**

Annotated odorant receptor sequences from *Drosophila melanogaster* (62 ORs) and *Musca domestica* (87 ORs) were used in tBLASTN searches of the *Stomoxys* genome to identify orthologs. This resulted in 74 ScalOr gene models, 71 of which were built by the NCBI automated annotation pipeline (Fig. S10; Additional File 8 and 9). Three new gene models were constructed, not including ones that resulted from fixing or splitting a model or from joining across scaffolds. Four gene models were short and included premature stop codons, and these are predicted to be pseudogenes identified with PSEU. An additional three gene models were partial, missing either the C terminal or N terminal portions, identified with CTE or NTE, respectively. The *Stomoxys* OR sequences were named based on relationship to *Musca*, as it is the closest relative with a sequenced genome. As in *Musca*, the OR naming system starts with the ortholog of DpOrN. The highly-conserved odorant co-receptor ORCO is present in *Stomoxys* (ScalORCO) and has high sequence similarity to *Drosophila* (88%) and *Musca* (96%). The transcript is detected in all life stages and tissues evaluated, including reproductive systems from mated females and males, supporting previous reports [94].

Sixteen simple 1:1:1 OR orthologs are shared between *Stomoxys, Musca*, and *Drosophila*, and these have relatively high amino acid sequence similarities. ScalOr64/MdomOr78 are orthologous to DmelOr85e (60% similarity), which detects 1R-(-)fenchone, a monoterpene that has a repellent effect towards insects [95]. *DmelOr85e* is co-expressed with *DmelOr33c*, which does not appear to have an ortholog in either *Stomoxys* or *Musca*. ScalOr10/MdomOr11 and ScalOr11/MdomOr12 are orthologs of DmelOr10a (60% similarity) and DmelOr13a (70% similarity) that are known receptors for methyl benzoate [96] and 1-octen-3-ol (octenol) [97], respectively. Methyl benzoate is a component of clove bud essential oil, known to be repellent towards *Stomoxys* [98], while octenol is a cattle-associated compound known to elicit strong EAG responses [99]. *DmelOr10a* is tandemly arranged and co-expressed with *DmelGr10a* [100]. In *Stomoxys*, DmelGR10a is dramatically expanded into 48 proteins, 30 of which surround *ScalOr10* at this locus; it is unclear whether any are co-expressed with *ScalOr10*. ScalOr60/MdOr70 are orthologs of DmelOr82a (50% similarity), which is known to selectively respond to geranyl acetate [101]; geranyl acetate elicits a moderate EAG response in *Stomoxys* (Hieu et al., 2014). ScalOr34/MdomOr42 are orthologs of DmelOr49b (63% similarity), which is responsive to *o*-, *m*-, and *p*-cresols [101], methylphenols that are volatile components of cattle and cattle dung and elicit strong responses in *Stomoxys* [102, 103]. ScalOr21/MdomOr23 and ScalOr15/MdomOr16 are orthologs of DmelOr43a (57%) and DmelOr24a (60%). DmelOr43a and DmelOr24a are receptors for 1-hexanol and propyl acetate, respectively [104]; 1-hexanol is typically identified from blends of green plant volatiles, which can comprise decaying vegetation of *Stomoxys* larval breeding substrates, while propyl acetate is a decomposition product of cattle manure. ScalOr62/MdomOr75 is orthologous to DmelOr85d (45%), which is a receptor for ethyl pentanoate and 2-heptanone [105], the latter of which elicits a weak EAG response in *Stomoxys* [106]. *Stomoxys* has several ORs that are orthologous to larval-specific or larval-expressed *Drosophila* ORs, yet do not share the larval expression pattern. These include: *ScalOr6* (female/male antennae, male proboscis)/DmelOr2a (larval, adult)/MdomOr4; *ScalOr43* (female/male antennae and proboscis)/DmelOr63a (larval)/MdomOr49; *ScalOr46* (female/male antennae)/DmelOr67b (larval, adult)/MdomOr51-52; *ScalOr58* (female/male antennae and proboscis)/DmelOr35a (larval, adult)/MdomOr67/68. *ScalOr66-67*/DmelOr94a-94b (co-expressed, larval-specific)/ MdomOr80. Other 1:1:1 orthologous relationships were: *ScalOr45*/DmelOr22c/MdomOr15; *ScalOr65*/DmelOr88a/MdomOr79.

ScalOr61 shares 50% identity with DmelOr85b and DmelOr85c, which are tandemly arranged in *Drosophila*. In *Musca*, *MdomOr71-74* are duplicated in tandem relative to *DmelOr85b/c*. *Stomoxys* microsynteny analysis supports orthology with *Drosophila*. However, *ScalOr61* and the neighboring TMEM135 domain containing gene are the only models on this scaffold possibly due to the presence of numerous gaps preventing meaningful gene model annotation. *DmelOr85b* is co-expressed with *DmelOr98b* in adults, and it is a receptor for butyl acetate and E3-hexenol; there does not appear to be an ortholog of *DmelOr98b* in either *Stomoxys* or *Musca*. *DmelOr85c* is expressed in larvae and is a receptor for 3-octanol and 1-heptanol [107]. *ScalOr61* expression was limited to adult tissues (adult heads, female/male antennae, male proboscis).

Smaller subsets of *Stomoxys* ORs duplicated relative to *Drosophila* are present in the genome. ScalOr35-38 clusters with DmelOr59a, which is a larval-specific receptor that responds to *o*-cresol. Within this group, ScalOr37 and 38 are pseudogenes and no larval RNA-Seq is detected for *ScalOr35* and *36* although *ScalOr36* was detected in female/male antennae and male proboscis. *ScalOr16* and *17* are expressed in female/male antennae but not in larvae, and these cluster with DmelOr30a, which is a larval-specific receptor that responds to *o*-cresol and *p*-cresol. ScalOr29-30 is in a sister clade to DmelOr49a A and B, which are the products of alternatively spliced *DmelOr49a* that is expressed in larvae and adults and binds *p*-cresol. There is no evidence for alternative splicing to produce these *Stomoxys* receptors. Although these *Stomoxys* receptors do not share a larval expression pattern with *Drosophila*, the response to methyphenols prevalent in cow dung could be co-opted for adult behaviors to locate hosts or ovipositional sites. Additional duplications relative to Drosophila ORs include ScalOr31-33/MdomOr37-41 that are in sister clades to DmelOr49a and DmelOr85f. *ScalOr31-33* are tandemly arranged, and *ScalOr33* is expressed in female/male antennae and proboscis. *DmelOr49a* and *DmelOr85f* are actually co-expressed, but unlinked in the genome (on different chromosomes), and together these mediate adult *Drosophila* detection and ultimate avoidance of semiochemicals produced by a parasitoid wasp, allowing the fly to detect ‘danger’ [108]. *ScalOr69* and *70* are tandemly arranged on a single scaffold and are duplicate orthologs of *DmelOr71a*, which is expressed in adults and binds ethylguaiacol and eugenol, components of host odors. ScalOr2 – 5, arranged on a single scaffold, are duplicates related to DmelOr1a, which is larval-specific, yet there is no evidence for larval expression of these *Stomoxys* receptors.

Muscid-specific lineages were also present, the most expansive being *ScalOr39-42* (single scaffold)/Mdom44-48. Others included *ScalOr7*/MdomOr5-6; *ScalOr8*/MdomOr7P-8; *ScalOr9*/MdomOr9-10; *ScalOr68*/MdomOr81; *ScalOr72*/ MdomOr83; *ScalOr73*/MdomOr84(A/B)

**Chemosensory Proteins (CSP)**

Chemosensory proteins (CSPs), also referred to as OS-D like and sensory appendage proteins, are a class of small, highly soluble molecules that have been associated with insect sensory organs and other non-chemosensory tissues [109]. CSPs have no sequence similarity to members of the OBP family, and they encode four cysteine residues believed to play a role in disulfide bridge formation. A diverse functional role for this family is proposed due to its expression in various tissues, such as pheromone glands [110] and the Drosophila ejaculatory duct (PebIII), making them attractive as targets for control. Seven CSPs were previously described, and manual curation identified 3 additional CSP sequences (Additional File 8). Eight of these ten CSPs are tandemly arranged on a single scaffold (KQ080226; Additional File 9).

**Gene families associated with metabolic detoxification**

**Contributors**: David Nelson (University of Tennessee) and Pia Olafson (USDA-ARS)

**Methods.**

Carboxylesterase, glutathione-S-transferase, and Cys-loop gated ion channel protein sequences from *Drosophila melanogaster* were used in tBLASTN searches to identify orthologs in the *S. calcitrans* 1.0.1 genome and M*. domestica* 2.0.2 genome assemblies. Amino acid sequences from each family were aligned with the MUSCLE algorithm [111], and the alignments trimmed with the trimAl tool using the –strictplus option [112]. The trimmed alignment was used to construct a maximum likelihood phylogeny with the web server version of IQ-TREE software ([113]; best-fit substitution model, branch support assessed with 1000 replicates of UFBoot bootstrap approximation). Resulting phylogenetic trees were unrooted.

Three families of CYPs are clustered along scaffolds in *S. calcitrans*: CYP9F, CYP6A, and CYP4D. To evaluate relationships of these genes between *S. calcitrans*, *M. domestica*, and *D. melanogaster*, the *S. calcitrans* amino acid sequences were used in a tBLASTn search of the M*. domestica* 2.0.2 genome assembly and the *D. melanogaster* genome to identify scaffolds or chromosomes housing the genes of interest. The amino acid sequences were obtained for all related *M. domestica* and *D. melanogaster* sequences, and the organization of these genes and those in the flanking regions were noted. Amino acid sequences from each family were aligned with the MUSCLE algorithm [111], and the alignments trimmed with the trimAl tool using the –strictplus option [112]. The trimmed alignment was used to construct a maximum likelihood phylogeny with the web server version of IQ-TREE software ([113]; best-fit substitution model, branch support assessed with 1000 replicates of UFBoot bootstrap approximation). Resulting phylogenetic trees (Fig. S20A-C), and CYP317A4 was used an outgroup to root the trees.

**Results.**

Carboxylesterases (COE; EC 3.1.1) have a role in the detoxification of organophosphate, carbamate, and pyrethroid classes of insecticides [114]. Insecticide resistance conferred by COEs can occur by different mechanisms, including amplification of COE gene copy number, elevated expression of COE transcripts, and gene mutations that enhance COE enzymatic activity [115, 116]. The COE gene family in *Stomoxys* is comprised of 44 genes encoding 47 catalytic and non-catalytic proteins (Fig. S16; Additional file 12). Thirty-six genes encode 38 secreted and intracellular catalytic proteins that separate into 7 clades, the largest of which includes 17 alpha-esterases. Majority of the alpha-esterase like genes are clustered along a single scaffold (KQ079923) with only 3 genes located separately, one of which encodes a partial sequence. Separate clades with representative secreted beta-esterases, juvenile hormone esterases, integument esterases, and glutactins are also present in *Stomoxys*. The COEs are presumed to be catalytic based on the presence of catalytic triad residues, with the exception of one glutactin protein (XP_013111473) and one juvenile hormone esterase-like protein (XP_013118535). Eight genes encode 9 non-catalytic proteins that are primarily involved in neurological development, including 4 neuroligins, gliotactin, and neurotactin (Fig. S17). A single acetylcholinesterase gene is also present in the *Stomoxys* genome, as previously described by Temeyer and Chen [117].

Glutathione S transferases (GST) are a family of enzymes that mediate the binding of reduced glutathione to xenobiotic or exogenous compounds that are electrophilic; this renders the compounds less reactive (detoxification) and promotes their excretion. GSTs typically encode residues for glutathione binding (G site) and substrate binding (H site), and these sites are located in the N- and C- terminus of the protein, respectively. Mutations within these binding sites and upregulation of GST expression have been correlated with resistance to insecticides, likely a result of increased capacity for xenobiotic detoxification [115, 118]. Insects encode microsomal and cytosolic GSTs, the latter of which are separated into six different classes (Delta, Epsilon, Omega, Sigma, Theta, and Zeta). The cytosolic gene family is comprised of 28 – 29 genes in mosquito species, 37 in *Drosophila*, and 26 in *Musca* [39, 119-121], while 36 genes encode 40 cytosolic GSTs and 5 genes encode 7 microsomal GSTs in the current *Stomoxys* genome assembly (Fig. S18; Additional file 12). Cytosolic genes encoding members of the Delta (16 genes) and Epsilon (9 genes) classes comprise 69% of the *Stomoxys* GST family, which is comparable to the distribution in *Drosophila* and *Musca*. Delta and Epsilon GSTs are unique to insects and have been implicated in insecticide resistance [122]. The *Stomoxys* Delta genes are clustered along a single scaffold (KQ080085) and are downstream of the S. calcitrans CYP9F expansion; 15 of the genes in the GST delta cluster, all with no introns, are arranged uninterrupted, which is consistent with D1 – D10 genes in *Drosophila* [116]. The five *Musca* delta genes also have no introns and are arranged uninterrupted on a single scaffold. The last *Stomoxys* Delta gene on this scaffold is alternatively spliced and shares sequence similarity and gene structure with *Drosophila* D11. In *Drosophila*, this gene is part of the uninterrupted cluster with D1 – D10, while the *Stomoxys* ortholog is separated from its cluster by a single, uncharacterized transcript.

*Cys-loop gated ion channels***.** Members of the Cys-loop gated ion channel (CysLGIC) superfamily are found throughout the insect nervous system mediating synaptic transmissions. As such, they are targets for several classes of insecticides [123, 124]. These channels are typically comprised of five subunits, each subunit of which encodes four transmembrane segments, an extracellular N-terminus that mediates ligand binding (loops A – F), and two cysteines that form a disulfide bond (‘Cys-loop’). In the *Stomoxys* genome, 26 genes were identified as putative CysLGIC subunits with orthologs to anionic and cationic channel subunits described from *Drosophila* (Fig. S19; Additional file 12). The nicotinic acetylcholine receptors (nAChRs), activated by acetylcholine, are targets for neonicotinoid (imidacloprid) and spinosyn insecticides, and are comprised of either 5 α subunits or three α and two β subunits. Alpha subunits are characterized by the presence of neighboring cysteine residues within one of the N-terminal loops (Loop C; YxCC motif), and these cysteines are required for acetylcholine binding [125]. As the β subunits do not encode these residues, it is thought they do not participate in ligand binding. In the *Stomoxys* genome, 12 nAChR genes were identified, nine of which encode the YxCC motif and were thus characterized as α subunits (α1, α2, α3, α4, α5, α6, and two α7, one of which was a partial sequence); those without were categorized as β1 and β2. While orthologs of *Drosophila* β2 from sequenced genomes of non-drosophilid insect species are categorized as a subunits [126-128], the β2 orthologs from *Stomoxys*, *Musca* (Scott et al., 2014), and Drosophila species [129] are of the β -type. Two divergent subunits (low sequence similarity to others) were also identified in *Stomoxys*, one each of an α- and β type. The majority of the loci were located on separate scaffolds, with the exception of α1/ α2 and the two divergent subunits that are arranged in tandem on separate scaffolds. The *Stomoxys* genome also encodes GABA-gated ion channel orthologs of Rdl, Lcch3, and GRD, and one glutamate-gated ion channel, which are known targets of cyclodiene, phenylpyrazole (fipronil), and avermectin insecticides. Further, two histamine-gated and two pH-sensitive chloride channels are present in the genome, as well as orthologs of as yet uncharacterized *Drosophila* CG8916, CG12344, NtR, and the ‘Insect Group 1’ class of CysLGICs [130].

**Lateral Gene Transfer Prediction**

Contributor: Jack Werren (University of Rochester), Perot Saelao (USDA-ARS)

**Results.** Results from the pipeline are summarized in Table S7. Three candidate LGTs were detected, all of which were derived from *Wolbachia*. The *Stomoxys* strain used for the genome sequencing was not infected with *Wolbachia*; however, this is not uncommon, and detection of these segments can be due to a *Wolbachia* infection in the progenitor. One LGT occurs on scaffold NW_013171927.1 (positions 130698-130899; bitscore 242) and corresponds to a portion of the *Wolbachia* protein coding gene DNA translocase ftsK-like. A second LGT occurs on scaffold NW_013171876.1 (position 680353-680446) and is from a B group Wolbachia (bitscore 134), with similarity to the porin gene. The third is found on NW_013172024.1 (positions 765568-765650; bitscore 98.7), but it’s similarity to a specific *Wolbachia* gene is uncertain, possibly due to divergence subsequent to lateral transfer. Expression of only one of these LGTs was detected based on our current RNA-Seq datasets: the LGT region in NW_013171876.1 (680353-680446; Wolbachia porin surface protein) shows expression within the predicted 3’ UTR for a transcription factor containing a basic leucine zipper domain. This apparent 3’ UTR region was not in the *in silico* annotated gene model, but it appears to be associated based on RNA-Seq data examined in the WebApollo browser at VectorBase. Whether expression of the LGT is biologically significant is unknown. It should also be kept in mind that the gene annotation may be in error, and this LGT could correspond to an expressed protein coding region.

***Stomoxys* Aquaporin Proteins
Contributors:** Christopher J. Holmes and Joshua B. Benoit (University of Cincinnati)

**Summary paragraph**

Nine putative aquaporin (aqp) proteins were identified within the *Stomoxys calcitrans* genome with observed expansion in the Prip/entomoglyceroporin gene families. Unsurprisingly, *S. calcitrans* shared the greatest similarity, as they both reside in the *Muscinae* family, with *Musca domestica* but also shared some similarity with *Lucilia cuprina* aqp homologues, with seven and two respectively. Gene expansion in Drip/Prip designations was previously observed and described in *M. domestica* and *Glossina morsitans* and was suggested as a means of increased or specialized transport of water in higher-order flies [42]. Interestingly, the expansion of Prip/entomoglyceroporin genes in *S. calcitrans* shares the greatest number of homologues with *M. domestica* (4/5), and *L. cuprina* (1/5). The total number of identified aquaporin genes in *S. calcitrans* (9) is comparable to other closely related dipterans, such as *G.* *morsitans* (10), *Aedes spp.* (6), and *Drosophila* *melanogaster* (8) [131]. A recent study with *L. cuprina* (7 aqps) postulated that aqp proteins act as major contributors to osmotic pressure regulation, saliva hydration, efficient digestion, *in vivo* offspring hydration, and cold/heat tolerance [132]. Another potential function of aqps is the provision of water for milk and subsequent lactation, as was observed in *G. morsitans* [133]. It is befitting that *S. calcitrans* should display similar specialized water transport, regulation, and offspring provisions as other members of the same order. These findings prompt further investigation on the role of *S. calcitrans* aqps in regards to survivability and fecundity.

**Highlights sentence**

Nine genes including three aquaporins, three entomoglyceroporins, two Pyrocoelia rufa integral proteins (PRIP), and a big brain protein were identified as putative aquaporin-like proteins in *S. calcitrans*.

**Supplementary Materials**

The annotated *Stomoxys* aqp genes are provided as a list in Table S12.

**Materials and Methods**

Putative aquaporin genes in the *S. calcitrans* genome were initially postulated via Gene Ontology term searches in Flybase (*Drosophila melanogaster*) by generating a query with proteins related to aquaporin-like functionality. The recovered nucleotide sequences were translated to peptide sequences and searched within the peptide models of *S. calcitrans*. For each possible aquaporin-like protein recovered from *S. calcitrans* models, the highest unique BLAST hit (blastp) was retrieved and confirmed via NCBI’s non-redundant arthropod database. Confirmed models were BLAST searched (blastp) against peptide sequences of *D. melanogaster, G. morsitans, L. cuprina,* and *M. domestica* for closest sequence homology. The *S. calcitrans* gene model was aligned to the closest homolog, manually annotated in WebApollo using homologues and RNA-Seq data, and searched against NCBI’s non-redundant arthropod database for a final confirmation.

**ADDITIONAL FILE 1 SUPPLEMENTARY TABLES**

**Table S1. RNA-Sequencing and Whole Genome Sequencing Accession Numbers and Statistics**

| **NCBI Bioproject** |  | PRJNA188117 |  |  | |  | |  | |  | |
| --- | --- | --- | --- | --- | --- | --- | --- | --- | --- | --- | --- |
| **NCBI SRA Study** |  | SRP018526 |  |  | |  | |  | |  | |
| **NCBI Transcriptome Shotgun Assembly** |  | GDIM00000000.1 |  |  | |  | |  | |  | |
| **Whole Genome Assembly; NCBI** Stomoxys_calcitrans-1.0.1 |  | GCF_001015335.1  Contigs: 125, 702 Scaffolds: 12,042 Scaffold N50: 504,651 bp  Final sequence Length: 971.2M bp  Final length (ungapped): 820.7M bp |  |  | |  | |  | |  | |
|  |  | **Comment** | **BioSample** | **Mbases** | | **Mbytes** | | **Run** | | **SRA_Sample** | |
| **RNA-Seq** |  |  |  |  | |  | |  | |  | |
| Adult female, teneral | F | Single whole, teneral adult female; 36M read pairs | SAMN01915459 | 12120 | | 8422 | | SRR694925 | | SRS392270 | |
| Adult Female, 7d fed, mated | Ffed | Single whole, 7d fed, mated, adult female; 46M reads | SAMN15814666 | 3430 | | 1610 | | SRR12450696 | | SRX8945140 | |
| Adult Female Head,  7d mated | FH | 4 heads; 7d mated female; 151M read pairs | SAMN03486501 | 28848 | | 19996 | | SRR1979467 | | SRS910173 | |
| Adult Female Reproductive System,  7d mated | FRS | Dissected tissue from 20 adult females, 7d mated; 118M read pairs | SAMN03486502 | 22473 | | 15189 | | SRR1979418 | | SRS910174 | |
| Adult male, teneral | M | Single whole, teneral adult male; 39M read pairs | SAMN01915460 | 12988 | | 9006 | | SRR694289 | | SRS392271 | |
| Adult Male Head,  7d mated | MH | 4 heads; 7d mated male; 133M read pairs | SAMN03486503 | 25343 | | 17163 | | SRR1979417 | | SRS910175 | |
| Adult Male Reproductive System, 7d mated | MRS | Dissected tissue from 30 adult males, 7d mated; 106M read pairs | SAMN03486500 | 20319 | | 14024 | | SRR1976146 | | SRS910172 | |
| Larva, 3^rd^ instar | L | Single, third instar larva; 34M read pairs | SAMN01915433 | 7158 | | 5053 | | SRR847512 | | SRS392224 | |
| Adult Female salivary glands,  7d mated | SG | Dissected salivary glands from 60 adult females, 7d; 47M reads | SAMN15804003 | 4660 | | 2560 | | SRR12439901 | | SRX8934554 | |
| Adult Male salivary glands, 7d mated | SG | Dissected salivary glands from 60 adult males, 7d; 24M reads | SAMN15804002 | 2390 | | 1310 | | SRR12439902 | | SRX8934553 | |
|  |  | **Comment** | **BioSample** | | **Mbases** | | **Mbytes** | | **Run** | | **SRA_Sample** |
| **Whole genome sequencing** |  |  |  | |  | |  | |  | |  |
|  |  | 13.4M read pairs | SAMN03486504 | | 5091 | | 3475 | | SRR1975009 | | SRS910642 |
|  |  | 15.2M read pairs | SAMN03486504 | | 5772 | | 3945 | | SRR1975010 | | SRS910642 |
|  |  | 13.2M read pairs | SAMN03486504 | | 5041 | | 3428 | | SRR1975042 | | SRS910642 |
|  |  | 80.8M read pairs | SAMN03486504 | | 30803 | | 21527 | | SRR1976147 | | SRS910642 |
|  |  | 90.1M read pairs | SAMN03486498 | | 34369 | | 22278 | | SRR1976148 | | SRS910170 |
|  |  | 92.6M read pairs | SAMN03486498 | | 35309 | | 23218 | | SRR1976149 | | SRS910170 |
|  |  | 100.6M read pairs | SAMN03486499 | | 38349 | | 25224 | | SRR1976154 | | SRS910171 |
|  |  | 18.4M read pairs | SAMN03486504 | | 6999 | | 4446 | | SRR1996564 | | SRS910642 |
|  |  | 18.3M read pairs | SAMN03486504 | | 6955 | | 4408 | | SRR1996566 | | SRS910642 |
|  |  | 17.4M read pairs | SAMN03486504 | | 6616 | | 4249 | | SRR1996567 | | SRS910642 |
|  |  | 17.5M read pairs | SAMN03486504 | | 6683 | | 4299 | | SRR1996568 | | SRS910642 |
|  |  | 19.8M read pairs | SAMN03486504 | | 7550 | | 4911 | | SRR1996620 | | SRS910642 |
|  |  | 16.7M read pairs | SAMN03486504 | | 6361 | | 4333 | | SRR1996621 | | SRS910642 |
|  |  | 19.1M read pairs | SAMN03486504 | | 7282 | | 4679 | | SRR1996622 | | SRS910642 |
|  |  | 24.9M read pairs | SAMN03486504 | | 9491 | | 6214 | | SRR1996623 | | SRS910642 |
|  |  | 24.5M read pairs | SAMN03486504 | | 9328 | | 6035 | | SRR1996624 | | SRS910642 |
|  |  | 15.5M read pairs | SAMN03486504 | | 5918 | | 3831 | | SRR1996625 | | SRS910642 |
|  |  | 19.4M read pairs | SAMN03486504 | | 7374 | | 4733 | | SRR1996626 | | SRS910642 |
|  |  | 20.5M read pairs | SAMN03486504 | | 7826 | | 5047 | | SRR1996627 | | SRS910642 |
|  |  | 15.7M read pairs | SAMN03486504 | | 5996 | | 3887 | | SRR1996628 | | SRS910642 |
|  |  | 43.4M read pairs | SAMN03486504 | | 16536 | | 10966 | | SRR1996629 | | SRS910642 |
|  |  | 20.6M read pairs | SAMN03486504 | | 7850 | | 5056 | | SRR1996630 | | SRS910642 |
|  |  | 17.7M read pairs | SAMN03486499 | | 6740 | | 4347 | | SRR1996631 | | SRS910171 |
|  |  | 28.6M read pairs | SAMN03486499 | | 10895 | | 7005 | | SRR1996632 | | SRS910171 |
|  |  | 42.8M read pairs | SAMN03486499 | | 16314 | | 10479 | | SRR1996633 | | SRS910171 |

**Table S2:** Summary of Dfam repeat elements with >1000 copies including number of unique elements for each family of elements and total number of genomic copies for each family.

| Type of repeat element | Family | Unique elements | Total copies |
| --- | --- | --- | --- |
| Class I (retrotransposons) | LINE | 6 | 19282 |
|  | LTR | 6 | 8223 |
| Class II (DNA transposons) | Cut and paste | 28 | 104621 |
|  | Rolling circle | 8 | 112877 |
| Transfer RNA | Valine | 1 | 5130 |
| Satellite | Undefined *Mus* satellite | 1 | 6236 |

**Table S3:** Detailed list of repeat elements with total genomic copy number >1000. Fifteen were found to be significantly enriched in association with at least one Muller element.

|  |  |  |  |  |  | Significantly enriched in Muller Element  (p < 4.291845e-05) | | | | | |
| --- | --- | --- | --- | --- | --- | --- | --- | --- | --- | --- | --- |
| Repeat Element | **Dfam Taxon**  **Described From** | **Total Genomic Copies** | **Type** | **Class** | **Superfamily** | **A** | **B** | **C** | **D** | **E** | **Unassigned** |
| DNAREP1_DM | *Drosophila melanogaster* | 90273 | DNA Transposon | Rolling Circle | Helitron | 2.45E-09 | 2.39E-12 |  |  | 2.40E-45 |  |
| Ginger1-N1_DR | *Danio rerio* | 42999 | DNA Transposon | Cut and Paste | Ginger | 1.03E-100 | 1.28E-16 | 9.64E-09 |  | 1.45E-50 |  |
| Baggins1 | *Drosophila melanogaster* | 9191 | Retrotransposon | LINE | LOA |  |  |  |  |  | 1.41E-14 |
| HAT1_DR | *Danio rerio* | 7997 | DNA Transposon | Cut and Paste | hAT-Ac | 1.20E-19 |  | 9.83E-05 |  | 3.71E-09 |  |
| Kolobok-2_DR | *Danio rerio* | 6927 | DNA Transposon | Cut and Paste | Kolobok-T2 | 1.03E-10 |  |  |  |  |  |
| IMPB_01 | *Mus musculus* | 6236 | Satellite | Satellite | Undefined | 4.58E-18 |  | 4.31E-05 |  | 1.19E-09 |  |
| HelitronY1_CE | *Caenorhabditis elegans* | 6096 | DNA Transposon | Rolling Circle | Helitron | 1.49E-13 | 7.66E-61 | 6.61E-60 |  | 1.33E-39 |  |
| HelitronY1A_CE | *Caenorhabditis elegans* | 5147 | DNA Transposon | Rolling Circle | Helitron | 1.44E-12 | 2.37E-45 | 4.19E-51 |  | 9.51E-37 |  |
| tRNA-Val-GTY | *Mus musculus*  *Homo sapiens*  *Danio rerio* | 5130 | ncRNA Gene | tRNA | Undefined |  |  |  |  |  |  |
| Vingi-1_CE | *Caenorhabditis elegans* | 3988 | Retrotransposon | LINE | Jockey |  |  |  | 5.09E-06 |  |  |
| HelitronY4_CE | *Caenorhabditis elegans* | 3987 | DNA Transposon | Rolling Circle | Helitron | 8.85E-11 | 1.62E-31 | 3.16E-36 |  | 2.91E-20 |  |
| DNA8-25_DR | *Danio rerio* | 3419 | DNA Transposon | Cut and Paste | Undefined |  |  |  |  |  |  |
| DNA-2-24_DR | *Danio rerio* | 2931 | DNA Transposon | Cut and Paste | Undefined |  |  |  |  |  |  |
| DNA-2-12_DR | *Danio rerio* | 2655 | DNA Transposon | Cut and Paste | CMC-EnSpm |  |  |  |  |  |  |
| Mariner2_DM | *Drosophila melanogaster* | 2536 | DNA Transposon | Cut and Paste | TcMar-Tc1 |  |  |  |  |  |  |
| Helitron-4_DR | *Danio rerio* | 2485 | DNA Transposon | Rolling Circle | Helitron |  |  |  |  |  |  |
| ACROBAT1 | *Danio rerio* | 2447 | DNA Transposon | Cut and Paste | TcMar-Stowaway? |  |  |  |  |  |  |
| IS3EU-4_DR | *Danio rerio* | 2373 | DNA Transposon | Cut and Paste | IS3EU |  |  |  |  |  |  |
| EnSpm-N13_DR | *Danio rerio* | 2329 | DNA Transposon | Cut and Paste | CMC-EnSpm |  |  |  |  |  |  |
| DNA9-3_DR | *Danio rerio* | 2190 | DNA Transposon | Cut and Paste | Undefined |  |  |  |  |  |  |
| Helitron3Na_Mam | *Mus musculus*  *Homo sapiens* | 2178 | DNA Transposon | Rolling Circle | Helitron |  |  |  |  |  | 6.03E-06 |
| Dada-tL_DR | *Danio rerio* | 2123 | DNA Transposon | Cut and Paste | Dada |  |  |  |  |  |  |
| LINEJ1_DM | *Drosophila melanogaster* | 1933 | Retrotransposon | LINE | Unknown |  |  |  |  |  |  |
| Dada-U6_DR | *Danio rerio* | 1804 | DNA Transposon | Cut and Paste | Dada |  |  |  |  |  |  |
| hAT-2n1_DR | *Danio rerio* | 1790 | DNA Transposon | Cut and Paste | hAT-Charlie |  |  |  |  |  |  |
| DNA-2-24B_DR | *Danio rerio* | 1783 | DNA Transposon | Cut and Paste | Undefined |  |  |  |  |  |  |
| Gypsy-31-LTR_DR | *Danio rerio* | 1777 | Retrotransposon | LTR | Gypsy |  |  |  |  |  |  |
| hAT-N68_DR | *Danio rerio* | 1745 | DNA Transposon | Cut and Paste | hAT-Ac |  |  |  |  |  |  |
| DNA8-21_DR | *Danio rerio* | 1703 | DNA Transposon | Cut and Paste | Merlin |  |  |  |  |  |  |
| DNA-8-24_DR | *Danio rerio* | 1628 | DNA Transposon | Cut and Paste | hAT? |  |  |  |  |  |  |
| RLTR20A4 | *Mus musculus* | 1557 | Retrotransposon | LTR | ERVK |  |  |  |  |  |  |
| DNA-1-2_DR | *Danio rerio* | 1543 | DNA Transposon | Cut and Paste | PIF-Harbinger? |  |  |  | 9.32E-05 |  |  |
| MamRTE1 | *Mus musculus*  *Homo sapiens* | 1533 | Retrotransposon | LINE | RTE-BovB |  |  |  |  |  |  |
| DNA8-26_DR | *Danio rerio* | 1533 | DNA Transposon | Cut and Paste | Undefined |  |  |  |  |  |  |
| RLTR20C1_MM | *Mus musculus* | 1500 | Retrotransposon | LTR | ERVK |  |  |  |  |  |  |
| Helitron-N2_DR | *Danio rerio* | 1500 | DNA Transposon | Rolling Circle | Helitron |  |  |  |  |  |  |
| Dada-tA_DR | *Danio rerio* | 1500 | DNA Transposon | Cut and Paste | Dada |  |  |  | 9.57E-06 |  |  |
| EnSpm-6N1_DR | *Danio rerio* | 1467 | DNA Transposon | Cut and Paste | CMC-EnSpm |  |  |  |  |  |  |
| R1_DM | *Drosophila melanogaster* | 1351 | Retrotransposon | LINE | R1 |  |  |  |  |  |  |
| RLTR20C2_MM | *Mus musculus* | 1344 | Retrotransposon | LTR | ERVK |  |  |  |  |  |  |
| DNA-8-5_DR | *Danio rerio* | 1317 | DNA Transposon | Cut and Paste | hAT |  |  |  |  |  |  |
| DMCR1A | *Drosophila melanogaster* | 1286 | Retrotransposon | LINE | CR1 |  |  |  |  |  | 9.85E-07 |
| DNA2-1_DR | *Danio rerio* | 1262 | DNA Transposon | Cut and Paste | CMC-EnSpm? |  |  |  |  |  |  |
| HSMAR2 | *Homo sapiens* | 1234 | DNA Transposon | Cut and Paste | TcMar-Mariner |  |  |  |  |  |  |
| Helitron1_CE | *Caenorhabditis elegans* | 1211 | DNA Transposon | Rolling Circle | Helitron |  |  | 5.09E-18 |  |  |  |
| EnSpm-N14_DR | *Danio rerio* | 1144 | DNA Transposon | Cut and Paste | CMC-EnSpm |  |  |  |  |  |  |
| HATN16_DR | *Danio rerio* | 1131 | DNA Transposon | Cut and Paste | hAT-Charlie |  |  |  |  |  |  |
| DNA-8-29_DR | *Danio rerio* | 1111 | DNA Transposon | Cut and Paste | Undefined |  |  |  |  |  |  |
| DIRS-4_DR | *Danio rerio* | 1035 | Retrotransposon | LTR | Ngaro |  |  |  |  |  |  |
| RLTR17B_Mm | *Mus musculus* | 1010 | Retrotransposon | LTR | ERVK |  |  |  |  |  |  |

**Table S4.** Autophagy genes identified from the *Stomoxys* genome

| **Gene** | **Description** | **Symbol** | ***Drosophila melanogaster*** | ***S.calcitrans* NCBI Assembly** | ***S.calcitrans* Trinity Assembly** | ***Glossina morsitans*** | ***Musca domestica*** | ***Lucilia cuprina*** |
| --- | --- | --- | --- | --- | --- | --- | --- | --- |
| Autophagy related gene 5 | Autophagy related gene 5 | Atg5 | FBpp0071023 | XM_013248121.1 | m.35060 | GMOY003449 | MDOA012336 | JRES01000960 |
| Autophagy related gene 8a | Gamma-aminobutyric_acid_receptor_associated_protein | Atg8a | FBpp0071427 | XM_013247737.1 | m.83112 | GMOY001732 | MDOA007273 | JRES01001093 |
| Autophagy related gene 2 | Vacuolar_protein_sortingprotein | Atg2 | FBpp0072840 | XM_013263117.1 | m.80493 | GMOY001443 | MDOA007571 | JRES01000902 |
| Autophagy related gene 101 | Autophagy related gene 101 | Atg101 | FBpp0074406 | XM_013242980.1 | m.25132 | GMOY008865 | MDOA008508 | JRES01001701 |
| Autophagy related gene 3 | Autophagy related gene 3 | Atg3 | FBpp0074822 | XM_013259825.1 | m.69533 | GMOY005990 | MDOA013560-PA | JRES01000502 |
| Autophagy related gene 1 | Autophagy related gene 1 | Atg1 | FBpp0075677 | XM_013256181.1 | m.10939 | GMOY006001 | MDOA012368-PB | JRES01000836 |
| Autophagy related gene 18a | Autophagy related gene 18a | Atg18a | FBpp0076414 | XM_013255840.1 | m.28075 | GMOY011950 | MDOA009867 | JRES01000681 |
| Autophagy related gene 18a | repeat_domain_phosphoinositide-interacting_protein_2 | Atg18a | FBpp0076415 | XM_013255840.1 | m.28075 | GMOY011950 | MDOA009867 | JRES01000681 |
| Autophagy related gene 18a | Autophagy related gene 18a | Atg18a | FBpp0076416 | XM_013255840.1 | m.28075 | GMOY011950 | MDOA009867 | JRES01000681 |
| Autophagy related gene 4 | Cysteine_proteaseprotein | Atg4 | FBpp0077618 | XM_013242582.1 | m.48533 | GMOY009754 | MDOA009284 | JRES01000438 |
| Autophagy related gene 17 | Autophagy related gene 17 | Atg17 | FBpp0078365 | XM_013250826.1 | m.47792 | GMOY006793 | ND | JRES01000235 |
| Autophagy related gene 18b | repeat_domain_phosphoinositide-interacting_protein_2 | Atg18b | FBpp0081016 | XM_013248067.1 | m.30380 | GMOY006489 | MDOA010683 | JRES01000477 |
| Autophagy related gene 13 | Autophagy related gene 13 | Atg13 | FBpp0081333 | XM_013246328.1 | m.17505 | GMOY005132 | MDOA005648 | JRES01001062 |
| Autophagy related gene 4 | Cysteine_proteaseprotein | Atg4 | FBpp0082569 | XM_013263016.1 | m.87204 | GMOY002013 | MDOA000103 | JRES01001623 |
| Autophagy related gene 8b | Gamma-aminobutyric_acid_receptor_associated_protein | Atg8b | FBpp0082957 | XM_013247737.1 | m.83112 | GMOY001732 | MDOA007273 | JRES01001093 |
| Autophagy related gene 6 | beclin-1-like_proteinprotein_codingScaffold | Atg6 | FBpp0083975 | XM_013255129.1 | m.29592 | GMOY009498 | MDOA015522 | JRES01000321 |
| Autophagy related gene 14 | Autophagy related gene 14 | Atg14 | FBpp0084739 | XM_013262975.1 | m.23678 | GMOY013071 | MDOA007570 | JRES01001623 |
| Autophagy related gene 16 | Autophagy related gene 16 | Atg16 | FBpp0084894 | XM_013257886.1 | m.57238 | GMOY003512 | MDOA011478 | JRES01000660 |
| Autophagy related gene 16 | Autophagy related gene 16 | Atg16 | FBpp0084895 | XM_013257886.1 | m.57238 | GMOY003512 | MDOA011478 | JRES01000660 |
| Autophagy related gene 16 | Autophagy related gene 16 | Atg16 | FBpp0084896 | XM_013257886.1 | m.57238 | GMOY003512 | MDOA011478 | JRES01000660 |
| Autophagy related gene 18 | Autophagy related gene 18 | Atg18 | FBpp0085890 | XM_013241783.1 | m.90446 | GMOY010920 | MDOA007482 | JRES01000960 |
| Autophagy related gene 7 | ubiquitin-like_modifier-activating_enzyme | Atg7 | FBpp0085891 | XM_013241783.1 | m.90446 | GMOY010920 | MDOA007482 | JRES01000960 |
| Autophagy related gene 9 | Autophagy related gene 9 | Atg9 | FBpp0086285 | XM_013263578.1 | m.5577 | GMOY009568 | MDOA015545 | JRES01000290 |
| Autophagy related gene 10 | ubiquitin-like-conjugating_enzyme | Atg10 | FBpp0112243 | XM_013242628.1 | m.60583 | GMOY013249 | MDOA015437 | JRES01000655 |
| Autophagy related gene 12 | Ubiquitin-like_protein_ATG12protein | Atg12 | FBpp0289288 | XM_013251101.1 | m.53617 | GMOY013029 | MDOA013564 | JRES01001708 |
| Autophagy related gene 1 | serine/threonine-protein_kinase | Atg1 | FBpp0289788 | XM_013256181.1 | m.10939 | GMOY006001 | MDOA012368 | JRES01000836 |
| Autophagy related gene 18a | Autophagy related gene 18a | Atg18a | FBpp0302862 | XM_013255840.1 | m.28072 | GMOY011950 | ND | JRES01000681 |
| Autophagy related gene 18 | repeat_domain_phosphoinositide-interacting_protein_2 | Atg18 | FBpp0302863 | XM_013255840.1 | m.28075 | GMOY011950 | MDOA009867 | JRES01000681 |
| Autophagy related gene 18 | Autophagy related gene 18 | Atg18 | FBpp0302864 | XM_013255840.1 | m.28072 | GMOY011950 | MDOA009867 | JRES01000681 |
| Autophagy related gene 9 | Autophagy related gene 9 | Atg9 | FBpp0302990 | XM_013263578.1 | m.5577 | GMOY009568 | MDOA015545 | JRES01000290 |
| Autophagy related gene 18b | repeat_domain_phosphoinositide-interacting_protein_2 | Atg18b | FBpp0304068 | XM_013248067.1 | m.30380 | GMOY006489 | MDOA010683 | JRES01000477 |
| Autophagy related gene 4a | Cysteine_proteaseprotein | Atg4a | FBpp0304824 | XM_013242582.1 | m.48533 | GMOY009754 | MDOA009284 | JRES01000438 |
| Autophagy related gene 2 | Vacuolar_protein_sortingprotein | Atg2 | FBpp0305632 | XM_013263117.1 | m.80493 | GMOY001443 | ND | JRES01000902 |
| Autophagy related gene 17 | RB1-inducible_coiled-coil_protein_1 | Atg17 | FBpp0306962 | XM_013250826.1 | m.47792 | GMOY006793 | MDOA014556 | JRES01000235 |
| Autophagy related gene 16 | Autophagy related gene 16 | Atg16 | FBpp0308289 | XM_013257886.1 | m.57240 | GMOY003512 | MDOA011478 | JRES01000660 |
| Autophagy related gene 10 | ubiquitin-like-conjugating_enzyme | Atg10 | FBpp0309509 | XM_013242628.1 | m.60583 | GMOY013249 | MDOA015437 | JRES01000655 |
| Autophagy related gene 8a | Gamma-aminobutyric_acid_receptor_associated_protein | Atg8a | FBpp0312069 | XM_013247737.1 | m.83112 | GMOY001732 | MDOA007273 | JRES01001093 |
| Autophagy related gene 8a | Gamma-aminobutyric_acid_receptor_associated_protein | Atg8a | FBpp0312070 | XM_013247737.1 | m.83112 | GMOY001732 | MDOA007273 | JRES01001093 |
| Autophagy related gene 18a | Autophagy related gene 18a | Atg18a | FBpp0312165 | XM_013255840.1 | m.28075 | GMOY011950 | ND | JRES01000681 |
| Autophagy related gene 8b | Gamma-aminobutyric_acid_receptor_associated_protein | Atg8b | FBpp0312339 | XM_013247737.1 | m.83112 | GMOY001732 | MDOA007273 | JRES01001093 |

**Table S5. Validation of *Stomoxys* transcript expression by qRT-PCR.** RNASeq experiments were based on a single sample, and conservative statistical analyses were used to report significant enrichment in an RNA-Seq dataset. Relative differences in expression were validated for a subset of these transcripts by qRT-PCR. Genes selected for validation were primarily identified as significantly enriched in an RNA-Seq dataset. Seven transcripts, shaded below, were not identified as significantly enriched. Relevant tissues analyzed and log_2_-fold change in RNA-Seq and qRT-PCR are summarized.

| **RefSeq ID (XM_0132_)** | **Primer F** | **Primer R** | **Tissues** | **RNASeq (log2 Fold Change)** | **RT-qPCR (log2 Fold Change)** |
| --- | --- | --- | --- | --- | --- |
| XM_013244756 | CTATGTCACCACCGAGGA | CGTATAGATAATAGCCAAGACTCT | FRS/MRS | 8.361933 | 10.78 |
| XM_013254582 | GCTTTGGCTGTAAAGGATTCAT | GCCGCAGATGTCCTAGTG | FRS/MRS | 15.1935 | 14.49 |
| XM_013263564 | GGAAGTTTCTGGTGCCTAT | TGCCCTATACCTCCATTGA | FRS/MRS | 6.030768 | 6.84 |
| XM_013261530 | AATTGGCCTTTAAGAGGACA | ATCGGTTGCTCGCTAATC | FRS/MRS | 8.160286 | 9.82 |
| XM_013243885 | AAGTACCACCACCACCTCCA | TTGGGACGGCTGGCACAT | FRS/MRS | -4.22814 | -4.75 |
| XM_013245554 | CCAGTAGCAAAGTCAGAGGA | TCGTTGTATTCTTGGTATGCTT | MRS/M | 11.0943 | 14.98 |
| XM_013251890 | GAGAATGATGTGGGTGAGAAGA | GTATCGTGACCCTCAAACATAATG | MRS/M | 2.55676 | 4.92 |
| XM_013254855 | GTTGGCGTATTGGCACAT | AGATAGATGTATGACCCTTGGG | MRS/M | -11.51679 | -12.9 |
| XM_013254859 | GTGATATTGGTGTGCGGATA | TATTGCGTTGACCCTTTGC | MRS/M | 11.413 | 13.72 |
| XM_013255380 | GCTGAAATCTGGCAATTACTA | AGGGCGAATATCTATTGTCA | MRS/M | 8.883967 | 12.75 |
| XM_013261331 | GAAGACAGCCAGACAACAA | GGAGATTCGTCACAATTCATTAAG | MRS/M | 8.434 | 12.99 |
| XM_013248964 | GGATTACAATCTACCCGAACAT | CAGACATTGCGTTGGAAGA | Fold/3rd instar larvae | 14.62629 | 12.61 |
| XM_013255359 | CAATGAAGACAATTTACGCCTTA | GCTGGATCTTGCGAATCA | Fold/3rd instar larvae | 6.658416 | 13.14 |
| XM_013260950 | AAGCTAATCTCTGCCATCTTTTACT | CAGGGATTTGTAGAAGGGTGT | Fold/3rd instar larvae | 9.024649 | 5.86 |
| XM_013257949 | ATGGATTGTAAAATAAGAGCAGTG | ATCAATATCATTTCGCAGGATTC | MRS/M | 9.421 | 7.11 |
| XM_013263755 | TTCTGTTGTTGGTGATGTCT | GAGAGTTGGAAGAGTTGATTGA | MRS/M | 7.47 | 5.98 |
| attacin_431326 | CGGACAATGCACCTTTGAG | GGAGCCTTCAGCAATTCG | MRS/M | 5.04999 | 2.67 |
| sarcotoxin_86450 | TGCAGTCACCACAGCTCATC | CAATGGAAACGCCCTTACTCAAG | Fold/3rd instar larvae | -2.51852 | 1.43 |
| stomoxyn | TCTGGTCGTTTTGGTGCTATGC | TCCGAAATGGTGTGCTTGACTT | F/3rd instar larvae | -7.993343 | -9.19 |
| ScOr14 | CAACGCTTATAAGTCTGTCTACCA | CGCTTCCATCACATCAGATTCG | MRS/M | 2.363646 | 6.58 |
| ScOr8 | ACAATCTTGGGAGGCTGAGGACAAC | GACAATCACCGACCACAGCACATAGA | MRS/M | 2.176579 | 2.16 |
| ScObp74 | ACAGCAAGAACGATCCTT | CCACATTGGGTGTATCATC | FH/MH | 4.520173 | 6.64 |
| ScObp76 | GGCTTTCTTGCAGGTCCTT | CCAGGGCTAAACACATTGAGA | FH/MH | -3.013485 | -5 |
| ScObp77 | ACTTATATTACGGTGTTGGTTGC | CTCGGGACAATCGGTTATCT | FH/MH | 2.189274 | 3.79 |
| ScObp60 | GTTTAGCTTCAGCGGCTTTGG | TGGCACAATCCTCACGAAATCT | FH/MH | 6.726963 | 4.62 |
| ScObp12 | GCTTACAGTTGCCAATGGTTTCA | CCTCTGGACTAATGCCCGATTC | FRS/Fold | 5.493931 | 8.33 |
| ScObp22 | GCAGTCAATGGAGAGGATCGTCT | ACCCTTCATTAGAGCCACAGCAG | FRS/Fold | 6.721475 | 6.07 |

**Table S6. Potential bacterial contaminating scaffolds located in the *Stomoxys* genome assembly**

| *Stomoxys* Scaffold | Scaffold Length | Average Read Depth  of Scaffold* | Closest Prokaryotic Match | GenBank Accession |
| --- | --- | --- | --- | --- |
| NW_013178664.1 | 3546 | 4.045 | *Treponema paraluiscuniculi* | NC_015714.1 |
| NW_013179950.1 | 2090 | 2.130 | *Variovorax paradoxus* | NC_014931.1 |
| NW_013180517.1 | 1785 | 5.565 | *Hyphomicrobium sp* | NC_015717.1 |
| NW_013180739.1 | 1687 | 4.900 | *Mycobacterium tuberculosis* | NC_002755.2 |
| NW_013180770.1 | 1673 | 5.310 | *Clostridium thermocellum* | NC_009012.1 |
| NW_013180965.1 | 1599 | 5.380 | *Methanobrevibacter ruminantium* | NC_013790.1 |
| NW_013182002.1 | 1320 | 1.540 | *Flexistipes sinusarabici* | NC_015672.1 |
| NW_013182503.1 | 1214 | 6.155 | *Idiomarina loihiensis* | NC_006512.1 |
| NW_013182963.1 | 1139 | 2.205 | *Methanocaldococcus jannaschii* | NC_000909.1 |
| NW_013183025.1 | 1131 | 3.995 | *Hyphomicrobium sp* | NC_015717.1 |
| NW_013183214.1 | 1102 | 5.935 | *Isosphaera pallida* | NC_014962.1 |
| NW_013183653.1 | 1028 | 5.300 | *Sebaldella termitidis* | NC_013517.1 |

*Calculated genome-wide average read depth per scaffold is 92.89

**Table S7. Predicted lateral gene transfer events identified from the *Stomoxys* genome**

| Scaffold | Scaffold Length | Average Read Depth of Scaffold* | Alignment  Start  End | Average Read Depth, LGT region* | Genbank  Accession | Closest Prokaryotic Match | Region of Interest | Notes |
| --- | --- | --- | --- | --- | --- | --- | --- | --- |
| NW_013171927.1 | 1468124 | 91.98 | 130001 131000 | 56.29 | NC_010981.1 | Wolbachia endosymbiont  Corrected prokaryotic blastmatch: Wolbachia confirmed  Corresponds to *Wolbachia* DNA translocase, *ftsK*-like | 130698-130899 | Wolbachia piece downstreanm from Orco,a ncRna and coding RNA in Stomoxys; no detectable expression in RNA-Seq datasets |
| NW_013171876.1 | 2185032 | 72.39 | 680001  681000 | 62.44 | NC_010981.1 | Wolbachia endosymbiont  Corrected prokaryotic blastmatch: B group Wolbachia (no strain best match)  Corresponds to *Wolbachia* porin gene (surface protein) | 680353-680446 | Within the 3’UTR of a transcription factor;  *Expression detected in RNA-Seq datasets |
| NW_013172024.1 | 994730 | 94.2 | 765001  766000 | 48.89 | NC_012416.1 | Wolbachia sp  Corresponds to ‘hypothetical protein’ of *Wolbachia* | 765568-765650 | downstream of Stomoxys mRNA, but not part of it; no detectable expression in RNA-Seq datasets |

*Calculated genome-wide average read depth per scaffold is 92.89

**Table S8**. Components of the immune deficiency, Toll, and JAK/STAT pathways identified from the *Stomoxys* genome

| **Immune Deficiency Pathway** | | | |
| --- | --- | --- | --- |
| PGRP-L | *See Table S7* |  |  |
| Imd (immune deficiency) | XP_013107116 | Death domain superfamily; receptor-interacting serine/threonine-protein kinase 1 |  |
| FADD | XP_013114910 | Death domain; Death effector domain |  |
| DREDD (caspase-8) | XP_013105770 |  |  |
| Relish (nuclear factor NF-kappa p110 subunit) | XP_013099422 | Rel homology domain (RHD), IPT domain, ankyrin repeats |  |
| Relish (nuclear factor NF-kappa p110 subunit) | XP_013100924 | Partial, RHD, IPT, ankyrin |  |
| akirin | XP_013103618 |  |  |
| IAP (inhibitor of apoptosis) | XP_013112429 | 2 BIR domains, I ring_Ubox domain |  |
| TAB2 (tak associated binding protein) | XP_013103394 | Zinc finger, CUE-TAB2/TAB3 ubiquitin binding domain |  |
| TAK1  Transforming growth factor activated kinas (MAP3K) | XP_013107045 | STKc-TAK1 domain (catalytic domain of serine-threonine kinase) |  |
| IKK (ird5, IKK-beta) | XP_013109447 | SPS1 (serine/threonine protein kinase) domain |  |
| IKK (kenny, IKK-gamma) | XP_013118213 | UBAN motif |  |
| Poor imd response upon knock-in (PIRK); e.g. PIMS | XP_013117266 | e-32; no conserved domains, but Dmel PIRK doesn’t have any either |  |
| PGRP-SC2/3 | *See Table S7* |  |  |
| Caspar (fas-associated factor 1, FAF) | XP_013102431 | Faf-1 UBX domain; UBA domain |  |
| caudal | XP_013114483 | Homeobox domain |  |
| mustard | XP_013098141 | TLDc domain; LysM domain |  |
| **Toll Pathway** | | | |
| PGRP-SA, PGRP-SD | *See Table S7* |  |  |
| Gram Negative Binding Proteins (GNBP)- GNBP3-like | XP_013116957 | Carbohydrate binding, Laminin G domains |  |
| GNBP3-like | XP_013108539 | Carbohydrate binding, Laminin G domains |  |
| GNBP1-like | XP_01311306 | Carbohydrate binding, Laminin G domains |  |
| GNBP2-like | XP_013113076 | Carbohydrate binding, Laminin G domains |  |
| spaetzle | XP_013115944 | Signal peptide |  |
| spaetzle processing enzyme (SPE) | XP_013102919 | Tryp-Spc, CLIP domain |  |
| Toll Receptor | XP_013107363 | N-terminal Leu-rich repeat, toll interleukin-1 resistance (TIR), TPKR_C2 domains |  |
| Myeloid differentiation primary response protein 88 (MyD88) | XP_013115653 | Death, TIR-2 domains |  |
| pelle (interleukin-1 receptor associated kinase [IRAK] 1) | XP_013102217 | C-terminal STKc_IRAK, N-terminal Death domains |  |
| tube (IRAK4) | XP_013116710 | Death domain |  |
| cactus (Rel-1 inhibitor) | XP_013100640 | Ankyrin repeats |  |
| dorsal | XP_013118266 | N-terminal Rel homology domain (RHD), C-terminal IPT_NKkB domain |  |
| dorsal immunity factor (dif) | XP_013118268 | N-terminal RHD, C-terminal Rel homology dimerization domains |  |
| deaf1 | XP_013116039 | SAND, MYND finger domain |  |
| drifter (dfr)/ventral veinless (vvl) | XP_013117919 | POU domain |  |
| persephone (psh) | XP_013119056 | Tryp_SPc (trypsin-like serine protease) domain |  |
| TNF receptor associated factor 6 (TRAF6) | XP_013107963 | RING, MATH, Sina domains |  |
| **JAK-STAT Pathway** | | | |
| unpaired (upd); ligand of domeless | XP_013114134 | UPD domain |  |
| upd | XP_013099717 | UPD domain |  |
| upd4-like | XP_013099693 | UPD domain |  |
| domeless receptor | XP_013097750 | Transmembrane domains; cytokine receptor motif; Fibronectin type 3 (FN3) domain |  |
| domeless receptor | XP_013108583 | Transmembrane domains; cytokine receptor motif; Fibronectin type 3 (FN3) domain |  |
| hopscotch | XP_013112402 | TyrKc, SH2 JAK domains |  |
| suppressor of cytokine signaling (SOCS);  SOCS36E-like | XP_013114164 | C-terminal SOCS box, SH2 domain, phosphotyrosine binding pocket |  |
| SOCS16D-like | XP_013103755 | C-terminal SOCS box, SH2 domain, phosphotyrosine binding pocket |  |
| SOCS44A-like | XP_013104964 | C-terminal SOCS box, SH2 domain, phosphotyrosine binding pocket |  |
| Signal transducer and activator of transcription (STAT) | XP_013119408 | SH2-STAT domain, STAT DNA binding and STAT protein interaction domains |  |
| Protein inhibitor of activated STAT (PIAS) | XP_013104891 | MIZ-SP/RING zinc finger, PINIT domains |  |

**Table S9. Manually annotated *Stomoxys* immune system gene family members**

| HMM Class | Aliases | Gene ID (LOC1060_) | OGS (XP0_) | GB Acc Scaff (KQ0_) | Coordinates | Strand | Introns | Amino Acids | Description |
| --- | --- | --- | --- | --- | --- | --- | --- | --- | --- |
| DEF |  | 96091 | NP_001298182 | 79966 | 263944-264234 | + | 0 | 97 | PREDICTED: Stomoxys calcitrans defensin-2 (LOC106096091), mRNA |
| DEF |  | 96096 | 13119119 | 79966 | 270901-270632 | - | 0 | 90 | PREDICTED: Stomoxys calcitrans phormicin-like (LOC106096096), mRNA |
| DEF |  | new gene model |  | 79966 | 277347-277111 | - | 0 | 79 |  |
| DEF |  | new gene model |  | 79966 | 281699-281947 | + | 0 | 83 |  |
| DEF |  | new gene model |  | 79966 | 282546-282809 | + | 0 | 88 |  |
| DEF |  | new gene model |  | 79966 | 295678-295950 | + | 0 | 91 |  |
| DEF |  | new gene model |  | 79966 | 299780-300052 | + | 0 | 91 |  |
| DEF |  | new gene model |  | 79966 | 304381-304653 | + | 0 | 91 |  |
| DEF |  | 96095 | 13119118 | 79966 | 307280-307543 | + | 0 | 88 | PREDICTED: Stomoxys calcitrans phormicin-like (LOC106096095), mRNA |
| DEF |  | 96093 | 13119114 | 79966 | 319216-319485 | + | 0 | 90 | PREDICTED: Stomoxys calcitrans phormicin-like (LOC106096093), mRNA |
| DEF |  | 95450 | 13118143 | LDNW01121039 | 1542-1948 | - | 0 | 90 | PREDICTED: Stomoxys calcitrans phormicin-like (LOC106095450), mRNA |
| ATT | attacin-A-like | 82486 | 13100487 | 80058 | 133592-134668 | + | 1 | 191 | PREDICTED: Stomoxys calcitrans attacin-A-like (LOC106082486), mRNA |
| ATT | attacin-A-like | 82493 | 13100495 | 80058 | 204517-205367 | - | 1 | 192 | PREDICTED: Stomoxys calcitrans attacin-A-like (LOC106082493), mRNA |
| ATT | attacin-A-like | 82495 | 13100498 | 80058 | 269002-269808 | + | 1 | 191 | PREDICTED: Stomoxys calcitrans attacin-A-like (LOC106082495), mRNA |
| ATT | attacin-A-like | 82494 | 13100496 | 80058 | 211944-212713 | + | 1 | 192 | PREDICTED: Stomoxys calcitrans attacin-A-like (LOC106082494), mRNA |
| ATT | attacin-A-like | 82496 | 13100499 | 80058 | 289374-290165 | - | 1 | 187 | PREDICTED: Stomoxys calcitrans attacin-A-like (LOC106082496), mRNA |
| ATT | attacin-A-like | 84469 | 13103625 | 80155 | 62657-63524 | + | 1 | 208 | PREDICTED: Stomoxys calcitrans attacin-A-like (LOC106084469), mRNA |
| ATT |  | 86445_Nterm |  | 80308 | 431252-432117 | + |  |  |  |
| ATT |  | 86445_Cterm |  | 80308 | 447548-448566 | + |  |  |  |
| ATT | sarcotoxin II-1-like | 86450 | 13106587 | 80308 | 457659-458647 | + | 1 | 241 | PREDICTED: Stomoxys calcitrans sarcotoxin II-1-like (LOC106086450), mRNA |
| ATT | sarcotoxin II-1-like | 86453 | 13106589 | 80308 | 491786-492703 | - | 1 | 239 | PREDICTED: Stomoxys calcitrans sarcotoxin II-1-like (LOC106086453), mRNA |
| ATT | attacin-A-like | 94198 | 13116854 | 82105 | 649-1416 | + | 1 | 209 | PREDICTED: Stomoxys calcitrans attacin-A-like (LOC106094198), mRNA |
| ATT | attacin-A-like | 94200 | 13116855 | 82105 | 7088-7961 | - | 1 | 208 | PREDICTED: Stomoxys calcitrans attacin-A-like (LOC106094200), mRNA |
| CEC | sarcotoxin-1D-like | 86080 | 13106064 | 80281 | 392346-393039 | - | 1 | 63 | PREDICTED: Stomoxys calcitrans sarcotoxin-1D-like (LOC106086080), mRNA |
| CEC | sarcotoxin-1C-like | 86087 | 13106070 | 80281 | 445443-445921 | + | 1 | 64 | PREDICTED: Stomoxys calcitrans sarcotoxin-1C-like (LOC106086087), mRNA |
| CEC | sarcotoxin-1B | 86088 | 13106071 | 80281 | 442402-442888 | - | 1 | 64 | PREDICTED: Stomoxys calcitrans sarcotoxin-1B (LOC106086088), mRNA |
| CEC | sarcotoxin-1C | 86085 | 13106067 | 80281 | 449880-450300 | - | 1 | 64 | PREDICTED: Stomoxys calcitrans sarcotoxin-1C (LOC106086085), mRNA |
| CEC | sarcotoxin-1C-like | 86086 | 13106068 | 80281 | 435492-435892 | - | 1 | 64 | PREDICTED: Stomoxys calcitrans sarcotoxin-1C-like (LOC106086086), mRNA |
|  | cecropin-like | new gene model |  | 80281 | 430591-430121 | + | 1 | 63 |  |
|  | cecropin-like | new gene model |  | 80281 | 420549-420090 | - | 1 | 63 |  |
| CEC |  | new gene model |  | 80281 | 402724-402273 | - | 1 | 60 |  |
|  | stomoxyn | new gene model |  | 80227 | 244950-245429 | + | 1 | 67 |  |
|  | stomoxyn | new gene model |  | 80227 | 236057-236472 | - | 1 | 67 |  |
| DIPT | diptericin-D-like | 86283 |  | 80295 | 406488-407012 | + | 1 | 96 | PREDICTED: Stomoxys calcitrans diptericin-D-like (LOC106086283), mRNA |
| DIPT |  | 86726 |  | 79933 | 768808-769513 | + | 1 | 107 | PREDICTED: Stomoxys calcitrans uncharacterized LOC106086726 (LOC106086726), mRNA |
|  | fungus-induced | 80461 | Gly-rich | 79975 | 192325-192740 | + | 1 | 63 | PREDICTED: Stomoxys calcitrans uncharacterized LOC106080461 (LOC106080461), ncRNA |
|  | fungus-induced | 80464 | Gly-rich | 79975 | 200748-201139 | + | 1 | 61 | PREDICTED: Stomoxys calcitrans uncharacterized LOC106080464 (LOC106080464), ncRNA |
|  | fungus-induced | 80451 | Gly-rich | 79975 | 197416-200370 | - | 1 | 61 | PREDICTED: Stomoxys calcitrans uncharacterized LOC106080451 (LOC106080451), ncRNA |
|  |  | 80476 |  | 79975 | 217702-218088 | - | 1 | 60 | PREDICTED: Stomoxys calcitrans uncharacterized LOC106080476 (LOC106080476), ncRNA |
|  |  | 80477 |  | 79975 | 220512-220952 | + | 1 | 60 | PREDICTED: Stomoxys calcitrans uncharacterized LOC106080477 (LOC106080477), ncRNA |
|  |  | 80470 |  | 79975 | 226477-227020 | - | 1 | 60 | PREDICTED: Stomoxys calcitrans uncharacterized LOC106080470 (LOC106080470), ncRNA |
|  |  | 80479 |  | 79975 | 227808-228212 | + | 1 | 56 | PREDICTED: Stomoxys calcitrans uncharacterized LOC106080479 (LOC106080479), ncRNA |
|  |  | 80463 | Gly-rich | 79975 | 232432-232831 | + | 1 | 56 | PREDICTED: Stomoxys calcitrans uncharacterized LOC106080463 (LOC106080463), ncRNA |
|  |  | 106080682 | 13097596 | 79979 | 1503661-1504420 | - | 1 | 183 | PREDICTED: Stomoxys calcitrans peptidoglycan-recognition protein SC2-like (LOC106080682), mRNA |
|  |  | 106081423 | 13098815 | 80013 | 319822-326860 | - | 2 | 199 | PREDICTED: Stomoxys calcitrans peptidoglycan-recognition protein SA-like (LOC106081423), mRNA |
|  |  | 106081421 | 13098810 | 80013 | 333796-337116 | - | 3 | 214 | PREDICTED: Stomoxys calcitrans peptidoglycan-recognition protein SA-like (LOC106081421), transcript variant X1, mRNA |
|  |  | 106081422 | 13098814 | 80013 | 340383-347773 | - | 2 | 201 | PREDICTED: Stomoxys calcitrans peptidoglycan-recognition protein SA-like (LOC106081422), mRNA |
|  |  | 106086541 | 13106721 | 80314 | 283987-296898 | - | 0 | 185 | PREDICTED: Stomoxys calcitrans peptidoglycan-recognition protein SC2 (LOC106086541), transcript variant X1, mRNA |
|  |  | 106086540 | split | 80314 | 296813-297421 | + | 0 | 185 | PREDICTED: Stomoxys calcitrans peptidoglycan-recognition protein SC2-like (LOC106086540), mRNA |
|  |  | 106086540 | split | 80314 | 288385-289012 | - | 0 | 185 |  |
|  |  | 106087784 | 13108408 | 80424 | 489768-490538 | - | 1 | 193 | PREDICTED: Stomoxys calcitrans peptidoglycan-recognition protein SD (LOC106087784), mRNA |
|  |  | 106088788 | 13109922 | 80536 | 50366-51128 | + | 1 | 183 | PREDICTED: Stomoxys calcitrans peptidoglycan-recognition protein SC2-like (LOC106088788), mRNA |
|  |  | 106088801 | 13109943 | 80536 | 69608-117691 | + | 2 | 197 | PREDICTED: Stomoxys calcitrans peptidoglycan-recognition protein LB-like (LOC106088801), transcript variant X1, mRNA |
|  |  | 106088839 | 13109991 | 80543 | 42818-115747 | + | 3 | 203 | PREDICTED: Stomoxys calcitrans peptidoglycan-recognition protein LA (LOC106088839), transcript variant X1, mRNA |
|  |  | 106088839 | 13109992 | 80543 | 42818-115747 | + | 4 | 293 |  |
|  |  | 106088839 | 13109993 | 80543 | 42818-115747 | + | 4 | 359 |  |
|  |  | 106088838 | 13109985 | 80543 | 189179-304877 | + | 3 | 665 | PREDICTED: Stomoxys calcitrans peptidoglycan-recognition protein LC (LOC106088838), transcript variant X1, mRNA |
|  |  | 106088838 | 13109988 | 80543 | 189179-304877 | + | 3 | 644 |  |
|  |  | 106088838 | 13109984 | 80543 | 189179-304877 | + | 3 | 675 |  |
|  |  | 106088838 | 13109987 | 80543 | 189179-304877 | + | 3 | 654 |  |
|  |  | 106088838 | 13109986 | 80543 | 189179-304877 | + | 3 | 663 |  |
|  |  | 106088838 | 13109989 | 80543 | 189179-304877 | + | 3 | 642 |  |
|  |  | 106088998 | 13110191 | 80565 | 254103-258308 | + | 0 | 187 | PREDICTED: Stomoxys calcitrans peptidoglycan-recognition protein SC2-like (LOC106088998), mRNA |
|  |  | 106089000 | 13110193 | 80565 | 262421-263106 | + | 0 | 187 | PREDICTED: Stomoxys calcitrans peptidoglycan-recognition protein SC2-like (LOC106089000), mRNA |
|  |  | 106089884 | 13111330 | 80683 | 251614-256518 | - | 5 | 303 | PREDICTED: Stomoxys calcitrans peptidoglycan recognition protein-like (LOC106089884), mRNA |
|  |  | 106089885 | 13111331 | 80683 | 256675-264060 | + | 5 | 345 | PREDICTED: Stomoxys calcitrans peptidoglycan recognition protein-like (LOC106089885), transcript variant X1, mRNA |
|  |  | 106089885 | 13111332 | 80683 | 256675-246060 | + | 5 | 326 |  |
|  |  | 106091807 | 13113924 | 81070 | 31286-45553 | - | 3 | 164 | PREDICTED: Stomoxys calcitrans peptidoglycan-recognition protein LD-like (LOC106091807), mRNA |
|  |  | 106092340 | 13114626 | 81213 | 158641-159591 | - | 2 | 187 | PREDICTED: Stomoxys calcitrans peptidoglycan-recognition protein SB1 (LOC106092340), mRNA |

**Table S10. RNA-Seq** Normalized expression values for transcripts annotated as antimicrobial peptides.

| **NAME** | Larvae - Stomoxys_larvae (GE) - Normalized expression values | Unfed Female - Female (GE) - Normalized expression values | Fed_Female - run1955_lane2_read1_indexD706=Sc_ff (GE) - Normalized expression values | Unfed Male - Male (GE) - Normalized expression values | MRS - Stomoxys_MRS (GE) - Normalized expression values |
| --- | --- | --- | --- | --- | --- |
| Smd1a | 0 | 9.862164852 | 306.3763235 | 12.73293756 | 16.3637603 |
| Smd2 | 0 | 18.08701884 | 195.3689182 | 19.09485561 | 0.09060004 |
| fbd1 | 0.24482459 | 0 | 53.54898468 | 0 | 192.232368 |
| fbd2 | 0.11425147 | 0 | 22.53069672 | 0 | 3.12918585 |
| defensin_LOC106096096 | 0 | 0.220223099 | 1.466038485 | 0.746319428 | 0.22998471 |
| defensin_KQ079966:281676-281972 | 0.03264328 | 0.134048843 | 289.1953812 | 0.318550975 | 0.09756927 |
| defensin_KQ079966:282510-282820 | 0 | 0.009574917 | 6.635753145 | 0.018202913 | 15.0674829 |
| defensin_KQ079966:295676-296020 | 0.93033343 | 0 | 0.128599867 | 0 | 0.0418154 |
| defensin_KQ079966:299778-300073 | 1.68112884 | 0 | 0.205759787 | 0 | 0.0627231 |
| defensin_KQ079966:304379-304706 | 0.24482459 | 0 | 0 | 0 | 0.0418154 |
| defensin_LOC106095450 | 0.55493573 | 0 | 4.140915722 | 0 | 25.3471023 |
| stomoxyn-2_KQ080227:236137-236429 | 0 | 0.229798016 | 32.76724615 | 0.600696125 | 0.0418154 |
| stomoxyn_KQ080227:244964-245373 | 0 | 253.8214836 | 1768.428213 | 195.3900668 | 0.51572328 |
| cecropin_KQ080281:402350-402665 | 0 | 0 | 0.154319841 | 0 | 0 |
| cecropin_KQ080281:420131-420477 | 14.9016566 | 0 | 0 | 0 | 0.54360022 |
| cecropin_KQ080281:430140-430565 | 0 | 0 | 0 | 0 | 0.89206189 |
| attacin_KQ080308:431326-432128 | 27.0775994 | 0 | 3.935155935 | 0 | 32.1281666 |
| attacin-partial_KQ080308:447549-448566 | 0.29378951 | 0 | 0 | 0 | 0.64116948 |
| attacin_LOC106082486 (m3516) | 1.2078013 | 0.009574917 | 0.334359655 | 0.072811652 | 0.52966175 |
| attacin_LOC106082493 (m3958) | 0.65286557 | 0 | 2.237637688 | 0 | 0.32755398 |
| attacin_LOC106082494 | 0 | 0 | 0.462959522 | 0 | 0.11150774 |
| attacin_LOC106082495 (m34203) | 8.96057991 | 0.076599339 | 28.62633043 | 0.172927672 | 2.15349316 |
| attacin_LOC106082496 | 0 | 0 | 0 | 0 | 3.6170322 |
| attacin_LOC106084469 (m20888) | 1.38733933 | 0 | 0.025719973 | 0 | 0.27876934 |
| attacin_LOC106094198 (m7797) | 63.9808256 | 0 | 36.85672192 | 0 | 533.048795 |
| attacin_LOC106094200 | 63.9808256 | 0 | 0.90019907 | 0 | 0.01393847 |
| Diptericin-D-like_LOC106086283 (DQ060072) | 0.16321639 | 0 | 5.504074314 | 0 | 54.2485138 |
| cecropin-1C-like_LOC106086086 | 0 | 0 | 0.334359655 | 0 | 0 |
| cecropin-1C_LOC106086085 | 0 | 0 | 1.157398804 | 0 | 38.7001538 |
| cecropin-1B_LOC106086088 | 0.22850295 | 0.009574917 | 1.003078964 | 0.027304369 | 1.36596977 |
| cecropin-1C-like_LOC106086087 | 0.01632164 | 0 | 3.034956865 | 0 | 0 |
| cecropin-1D_LOC106086080 | 0 | 0 | 0.180039814 | 0 | 5.9656639 |
| cecropin-1_LOC106086450 (m54180) | 20.7937683 | 0 | 2.803477104 | 0 | 29.814381 |
| cecropin-1_LOC106086453 (m2963) | 20.7937683 | 0.019149835 | 0.128599867 | 0.036405826 | 0 |

**Table S11. Stomoxys opsin gene compilation.**

|  | target_id  Vectorbase ID  Genbank GeneID | length | UM_tpm+1 | UF_tpm+1 | MRS_tpm+1 | MH_tpm+1 | FRS_tpm+1 | FH_tpm+1 | FH/UF | FH/FRS | MH/UM | MH/MRS |
| --- | --- | --- | --- | --- | --- | --- | --- | --- | --- | --- | --- | --- |
| Rh1.1.1.1 | SCAU002283-RA  LOC106092322 | 1806 | 3210.43 | 4040.76 | 4.25542 | 34483.2 | 2.81284 | 17728.8 | 4.38749146 | 6302.81139 | 10.7409911 | 8103.35995 |
| Rh1.1.1.2.1 | SCAU002003-RA LOC106092325 | 1677 | 46.3742 | 29.4257 | 1.0501011 | 446.046 | 1.0357606 | 231.278 | 7.85972806 | 223.292912 | 9.61840851 | 424.764816 |
| Rh1.1.1.2.2 | SCAU002003-RB LOC106092325 | 1499 | 2.94639 | 3.76631 | 1.617245 | 5.78935 | 1.00810172 | 3.06827 | 0.8146621 | 3.04361151 | 1.96489603 | 3.57976064 |
| Rh1.1.2 | SCAU010102-RA LOC106092323 | 1208 | 3.12637 | 1.288943 | 1.0453179 | 1.115424 | 1 | 1.0666305 | 0.8275234 | 1.0666305 | 0.35677927 | 1.06706677 |
| Rh1.2.1 | SCAU009433-RA LOC106092326 | 1602 | 12.0143 | 15.0164 | 1.0832298 | 632.387 | 1.0325571 | 347.152 | 23.1181908 | 336.206104 | 52.6361919 | 583.797639 |
| Rh1.2.2 | SCAU001182-RA LOC106092327 | 1566 | 58.7621 | 62.1951 | 1.108109 | 341.086 | 1.0669196 | 819.178 | 13.1711019 | 767.797311 | 5.80452366 | 307.809069 |
| Rh2 | SCAU000443-RA LOC106091691 | 1578 | 32.6909 | 34.0762 | 2.13895 | 743.419 | 2.3083 | 638.845 | 18.7475423 | 276.759953 | 22.7408545 | 347.562589 |
| Rh3 | SCAU016603-RA LOC106092178 | 1568 | 26.595 | 26.9322 | 3.34129 | 559.202 | 1.177219 | 462.007 | 17.1544471 | 392.456289 | 21.0265839 | 167.361109 |
| Rh5 | SCAU010746-RA LOC106094954 | 1365 | 26.8907 | 35.0216 | 1.0788332 | 296.384 | 1.0360057 | 212.174 | 6.0583754 | 204.800032 | 11.0218031 | 274.726436 |
| Rh6 | SCAU014862-RA LOC106081480 | 1595 | 1.923321 | 1.694455 | 1.0132407 | 5.49033 | 1 | 5.81593 | 3.43233075 | 5.81593 | 2.85460929 | 5.41858415 |
| Rh7 | SCAU012939-RA LOC106092023 | 2333 | 2.3498 | 2.3202 | 1.170531 | 19.0955 | 1.00499463 | 10.71327 | 4.61739074 | 10.6600271 | 8.12643629 | 16.3135363 |

**Table S12. Aquaporin gene names and corresponding symbols, model numbers, scaffolds, and arthropod homologues.** Bolded gene model numbers within the closest homologue designation represent highest homology to the proposed S. calcitrans gene model.

| Gene Name (from NCBI) | Gene Symbol | *Stomoxys calcitrans* gene model | Scaffold | *Drosophila melanogaster* | *Glossina morsitans* | *Lucilia cuprina* | *Musca domestica* |
| --- | --- | --- | --- | --- | --- | --- | --- |
| big brain (iso. A) | bib | SCAU014254 | KQ080825 | NP_476837.1 | GMOY012056 | KNC31443 | **XP_005178238.1** |
| prip (iso. A) | Prip | SCAU000117 | KQ081130 | NP_725052.1 | GMOY012008 | **KNC29013** | XP_005183592.1 |
| prip (iso. A) | Prip | SCAU006917 | KQ081382 | NP_725052.1 | GMOY012008 | KNC31435 | **XP_005186075.1** |
| aquaporin | AQP | SCAU004662 | KQ080861 | NP_001295945.1 | GMOY003126 | **KNC23793** | XP_005186069.1 |
| aquaporin | AQP | SCAU014220 | KQ080825 | NP_001295945.1 | GMOY003126 | KNC31439 | **XP_005186069.1** |
| aquaporin (iso. B) | AQP | SCAU004786 | KQ081172 | NP_001163140.1 | N/A | KNC32292 | **XP_005187711.1** |
| entomoglyceroporin 4 (iso. D) | Eglp4 | SCAU016126 | KQ080167 | NP_726349.1 | GMOY009439 | KNC34900 | **XP_005183004.2** |
| entomoglyceroporin 4 (iso. E) | Eglp4 | SCAU001623 | KQ080167 | NP_001246482.1 | GMOY009435 | KNC24942 | **XP_005183000.1** |
| entomoglyceroporin 2 (iso. A) | Eglp2 | SCAU005926 | KQ081758 | NP_611811.3 | GMOY009435 | KNC24942 | **XP_005184624.1** |

**Additional File 1, Supplementary Figures**

| **Figure S1.** | Phylogenetic placement and genomic comparisons for *Stomoxys calcitrans* and other fly species |
| --- | --- |
| **Figure S2.** | Pearson's correlation of RNA-Seq and RT-qPCR results |
| **Figure S3.** | Average read depth across predicted lateral gene transfer candidates |
| **Figure S4.** | *Stomoxys calcitrans* genomic scaffold housing 11 defensin gene models |
| **Figure S5.** | Maximum likelihood phylogenetic tree of PGRP protein sequences from *S. calcitrans* (red), *M. domestica* (black), and *D. melanogaster* (blue) |
| **Figure S6.** | Alignment of *Stomoxys* PGRP-S sequences with characterized *D. melanogaster* PGRP-SC1 (C0HK98) and –SC2 (Q9VX2) and N-acetylmuramoyl-L-alanine amidase (P00806). |
| **Figure S7.** | Alignment of *Stomoxys* PGRP-L sequences with characterized *D. melanogaster* PGRP-L proteins and N-acetylmuramoyl-L-alanine amidase. |
| **Figure S8.** | *Stomoxys calcitrans* Odorant Binding Protein Gene Family |
| **Figure S9.** | Duplicated OS-E-like and an OS-X ortholog in *Stomoxys*  and *Musca* |
| **Figure S10.** | *Stomoxys calcitrans* Odorant Receptor Gene Family |
| **Figure S11.** | *Stomoxys calcitrans* Gustatory Receptor Gene Family |
| **Figure S12.** | *Stomoxys calcitrans* Ionotropic Receptor Gene Family |
| **Figure S13.** | Maximum likelihood tree of dipteran opsin gene relationships |
| **Figure S14.** | Phylogenetic analysis of the Stomoxys Rh1 gene cluster and Genomic organization and evolution of the S. calcitrans Rh1 opsin subfamily |
| **Figure S15.** | Analysis of tuning site 17 variation in Stomoxys and Musca Rh1 paralogs |
| **Figure S16.** | Phylogenetic relationship of catalytic carboxyesterases from *Stomoxys* relative to *Musca* and *Drosophila*. |
| **Figure S17.** | Phylogenetic analysis of carboxylesterases with a role in neuronal development |
| **Figure S18.** | Phylogenetic analysis of glutathione-S-transferases from *Stomoxys* relative to *Musca* and *Drosophila*. |
| **Figure S19.** | Phylogenetic analysis of Cys-Loop Ligand Gated Ion Channels from *Stomoxys* relative to *Musca* and *Drosophila*. |
| **Figure S20.** | Cytochrome P450 (CYP450) genes clustered on *Stomoxys* scaffolds and evidence for expansions in muscids relative to *Drosophila*. |
| **Figure S21.** | Phylogenetic analysis of cytochrome P450 genes from *Stomoxys calcitrans* |

**Figure S1: Phylogenetic placement and genomic comparisons for *Stomoxys calcitrans* and other fly species.** Left, The phylogenetic analysis places *S. calcitrans* as a sister species to the house fly, *Musca domestica*. The phylogeny is built using RAxML and it is based on amino acid sequences from 664 single-copy genes that are present in all nine species. Bootstrap values are shown for every node. Middle, Benchmarking Universal Single-Copy Orthologs (BUSCO, [134] analyses based on the dipteran dataset (odb8). Blue, genome; Red, predicted genesets. Right, Orthology-based analyses of protein coding genes between eight fly species determined the number of genes in single-copy core clusters, variable-copy number core clusters, paraphyletic clusters (non-core, non-species specific), singleton, and species-specific clusters, based on OrthoFinder [5].


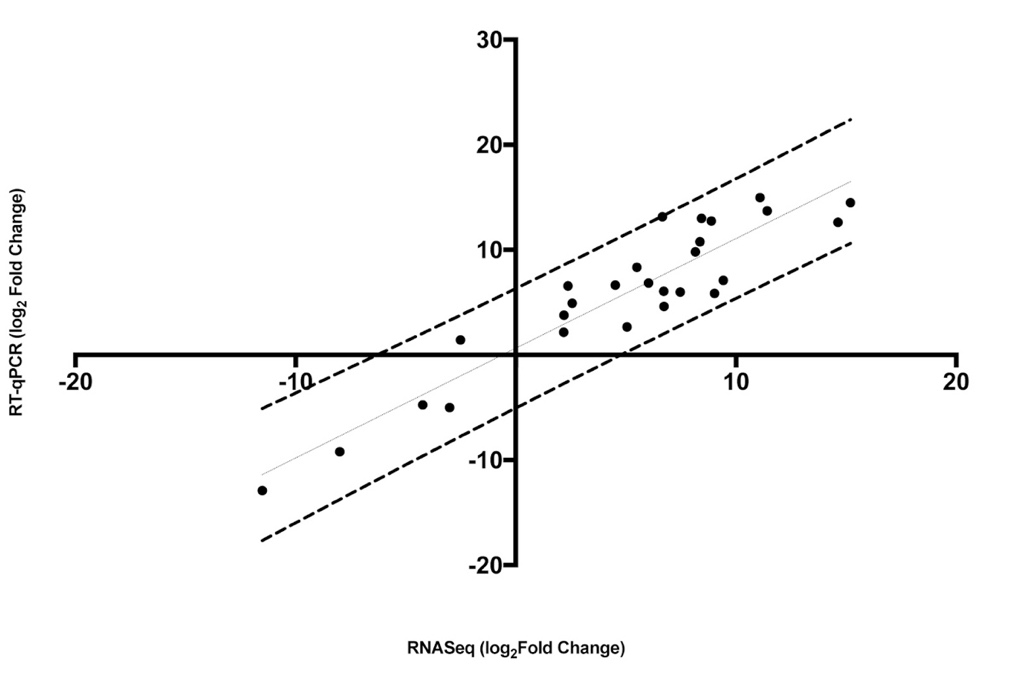


**Figure S2. Pearson's correlation of RNA-Seq and RT-qPCR results.** The correlation of the log_2_ fold change analyzed by RNA-Seq (x-axis) with data obtained using RTq-PCR (y-axis). Dashed lines represent the 95% confidence interval.

**Figure S3. Average read depth across predicted lateral gene transfer candidates.** The average read depth coverage of LGTs and +/- 1 kb regions spanning the junction sites of their corresponding scaffold. Vertical lines represent junctions. Each point is a ratio of the average read depth of a given 50 bp window divided by the average 50 bp read depth of the entire corresponding scaffold. Box plots depict the distribution of coverage of the entire scaffold relative to the whole genome average.


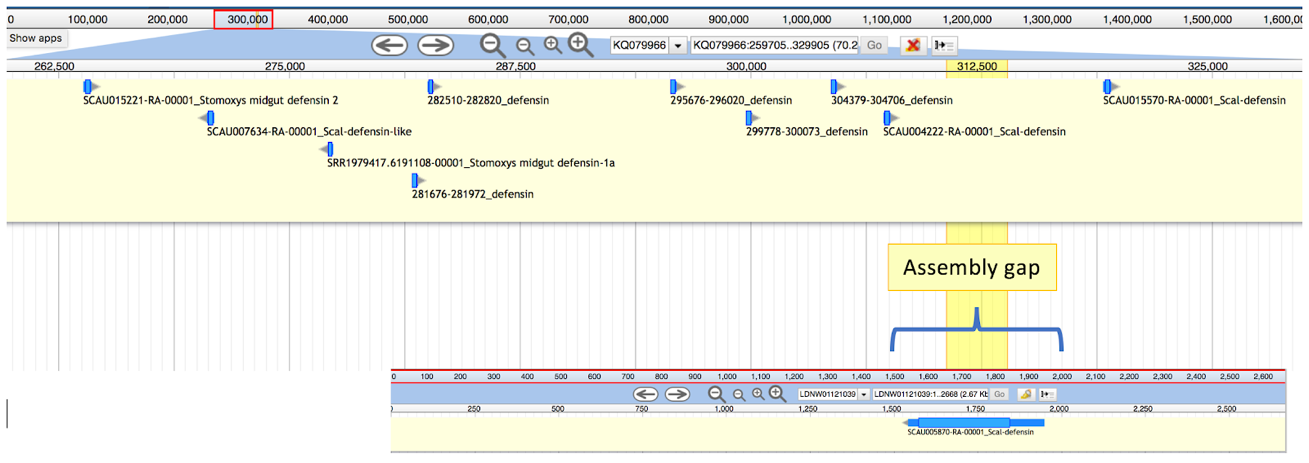


**Figure S4**. *Stomoxys calcitrans* scaffold KQ079966 housing 11 defensin gene models.


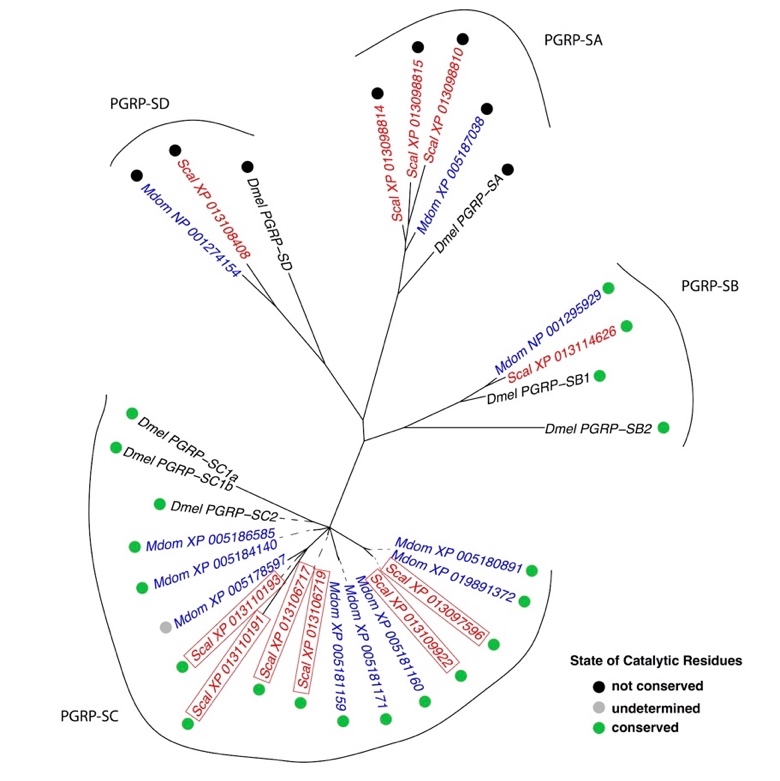

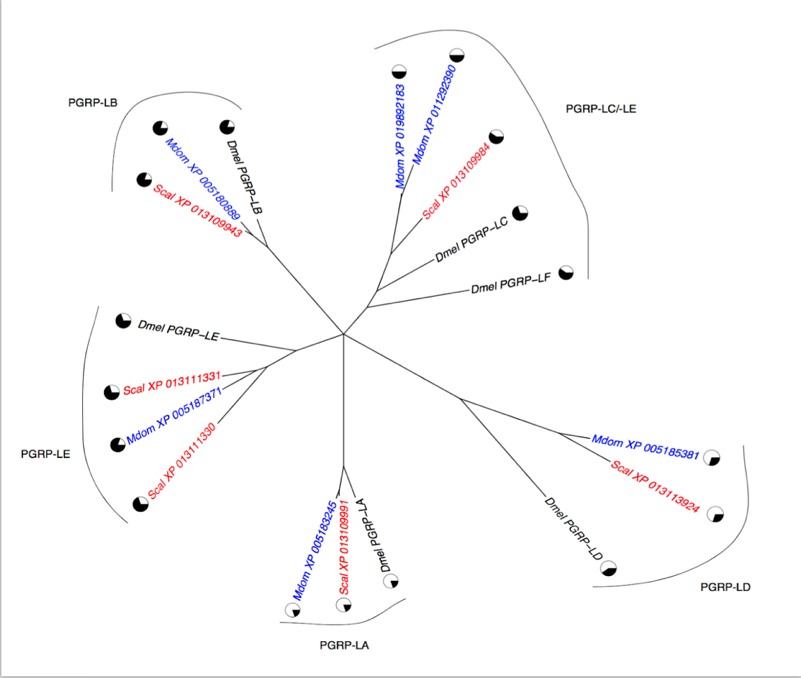


**Figure S5. Maximum likelihood phylogenetic tree of PGRP protein sequences from *S. calcitrans* (red), *M. domestica* (black), and *D. melanogaster* (blue)**. Alignments of PGRP-S and PGRP-L sequences were produced separately using mafft (einsi option), and then trimmed to remove columns with >75% gaps with trimal. Phylogenetic trees were computed with RAXML using the PROTGAMMAWAG model and bootstrapped 100 times. Nodes with < 50% bootstrap support are collapsed. Larval-specific *S. calcitrans* PGRP-S sequences have a box around their label. Circles indicate presence/absence of catalytic residues (PGRP-S), or the proportion of sites important for PGRP binding conserved (PGRP-L).


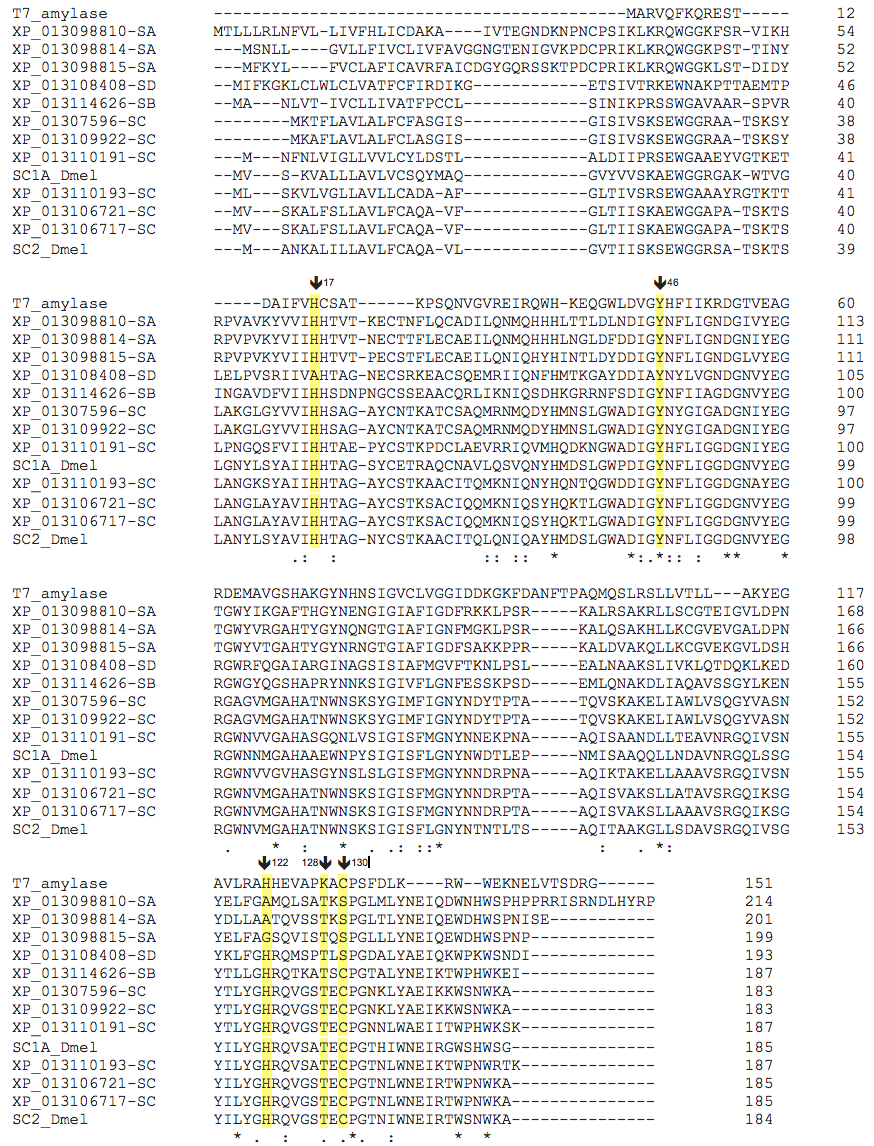


**Figure S6.** Alignment of *Stomoxys* PGRP-S sequences with characterized *D. melanogaster* PGRP-SC1 (C0HK98) and –SC2 (Q9VX2) and N-acetylmuramoyl-L-alanine amidase (P00806). Conserved amino acid residues required for catalytic amidase activity are highlighted in yellow [26]; conservation of cysteine at position 130 supports potential catalytic activity of *Stomoxys* PGRP-SC sequences.


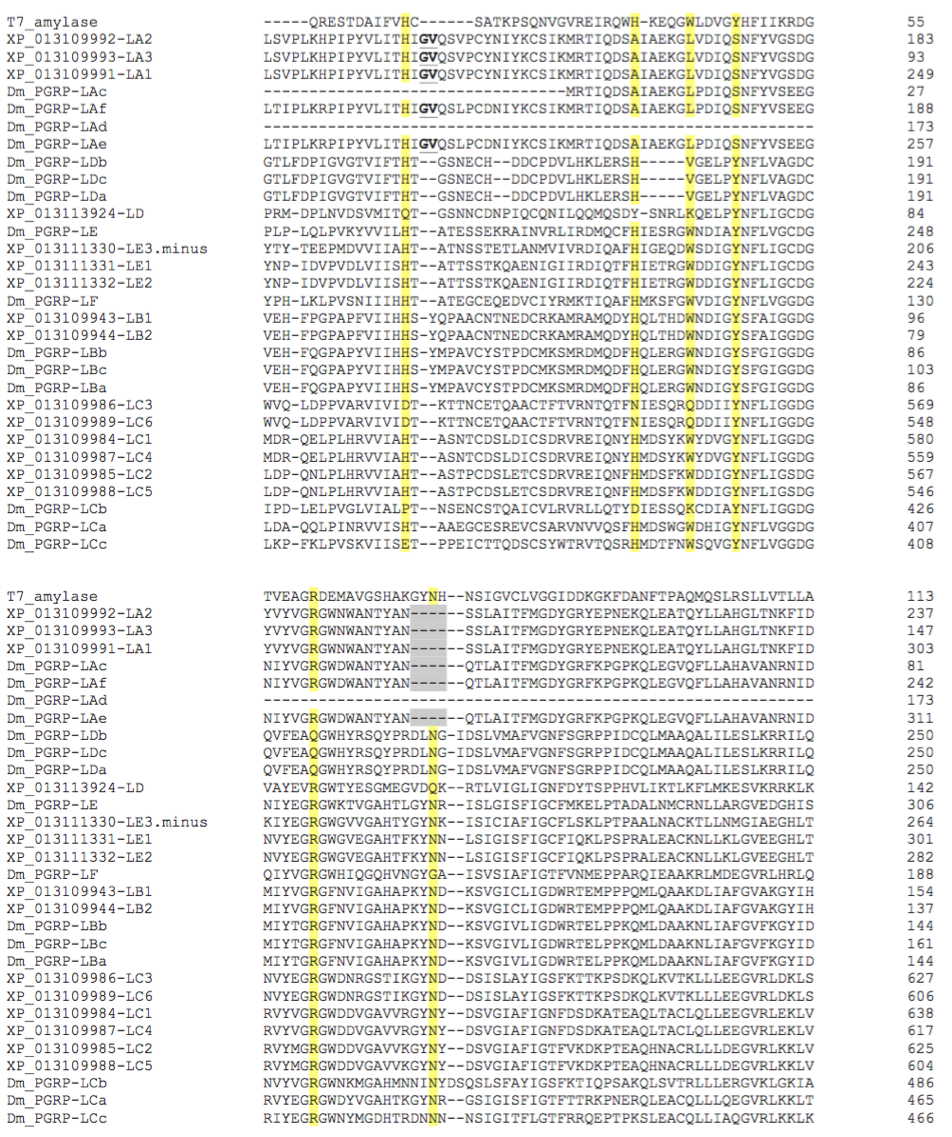

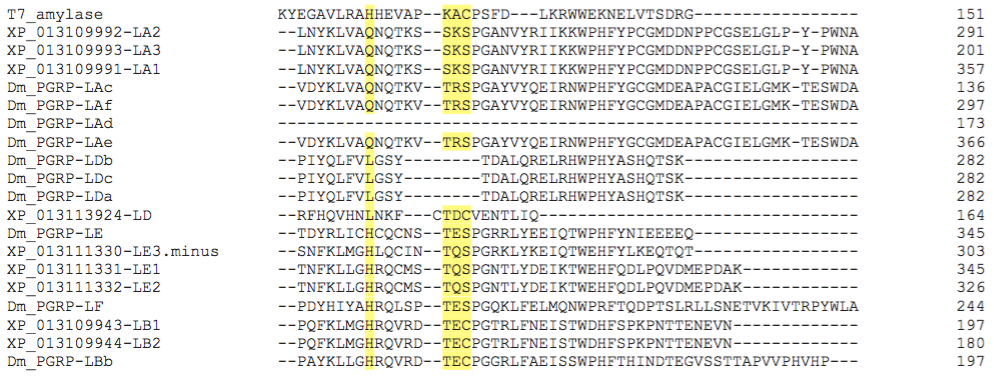

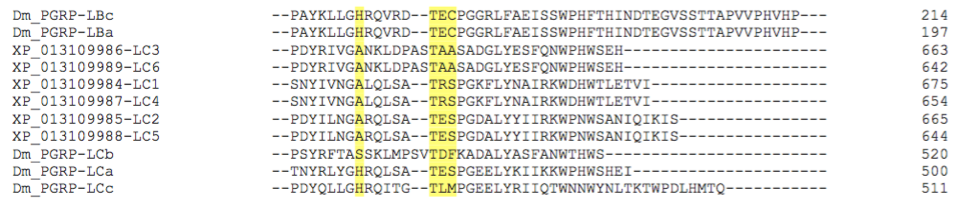


**Figure S7.** Alignment of *Stomoxys* PGRP-L sequences with characterized *D. melanogaster* PGRP-L proteins and N-acetylmuramoyl-L-alanine amidase. Conserved amino acid residues required for peptidoglycan binding and catalytic amidase activity are highlighted in yellow [26, 135]. Of the ten residues that are critical for peptidoglycan binding [135], the *Stomoxys* -LB, -LC and –LE sequences encode 9, 8, and 8 of these. Only 2 of these residues are conserved in the *Stomoxys* –LA and –LD sequences. Further, the *Stomoxys* -LA sequences encode a 2 amino acid insertion (bold, underlined text) and a 4 amino acid deletion (grey shaded) in regions of the molecules that are critical to binding [135]. These suggest that –LA and –LD do not bind peptidoglycan but may have a regulatory role [31].

**Fig. S8-A.**

**Fig. S8-B.**

**Figure S8. Stomoxys Odorant Binding Protein Gene Family. A.** Phylogenetic tree of the *Stomoxys calcitrans* OBPs with those of *Drosophila melanogaster* and *Musca domestica.* The *S. calcitrans* and *M.* *domestica* gene/protein names are highlighted in teal and blue, respectively, while *D. melanogaster* names are in mustard. Maximum likelihood phylogeny was constructed using the web server version of IQ-TREE software (best-fit substitution model, branch support assessed with 1000 replicates of UFBoot bootstrap approximation, bootstrap percentage presented). **B.** Heat map of normalized expression values for *Obp* transcripts annotated from the *Stomoxys* genome.

**Figure S9.** **OS-E-like and an OS-X ortholog in *Stomoxys* and *Musca*.** Phylogenetic analysis with OS-E, OS-F, and OS-X proteins from *Drosophila* sp.

**Fig. S10-A**


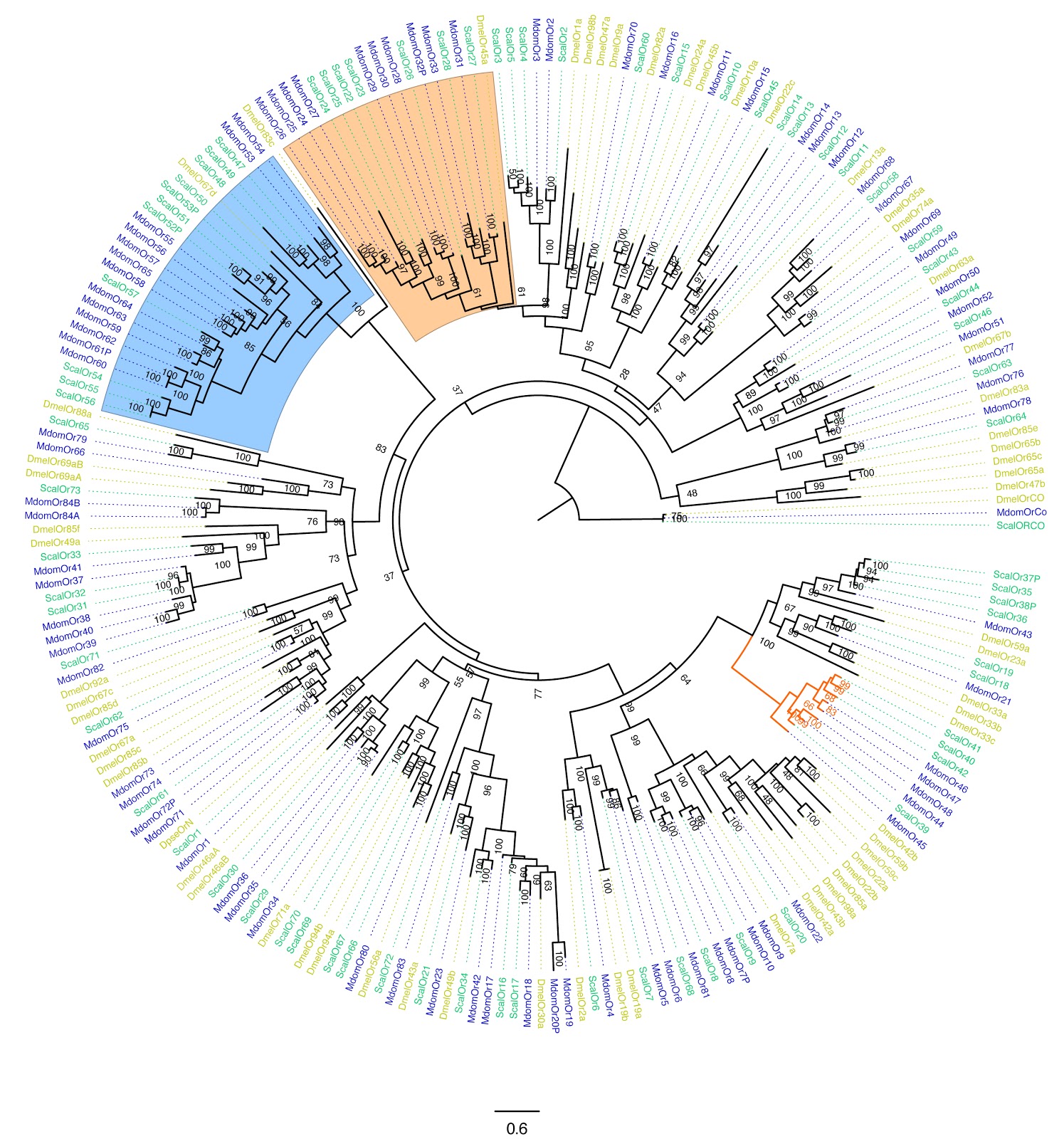


**Fig. S10-B.**

**Figure S10. Stomoxys Odorant Receptor Gene Family. A.** Phylogenetic tree of the *Stomoxys calcitrans* ORs with those of *Drosophila melanogaster* and *Musca domestica.* The *S. calcitrans* and *M.* *domestica* gene/protein names are highlighted in teal and blue, respectively, while *D. melanogaster* names are in mustard. Maximum likelihood phylogeny was constructed using the web server version of IQ-TREE software (best-fit substitution model, branch support assessed with 1000 replicates of UFBoot bootstrap approximation). **B.** Heat map of normalized expression values for *Or* transcripts annotated from the *Stomoxys* genome.

**Fig. S11-A.**

**Fig. S11-B.**

**Figure S11. *Stomoxys* Gustatory Receptor Gene Family A.** Phylogenetic tree of the *Stomoxys calcitrans* GRs with those of *Drosophila melanogaster* and *Musca domestica.* This is a maximum likelihood tree rooted by declaring the distantly-related and divergent carbon dioxide and sugar receptor subfamilies as the outgroup. The *S. calcitrans* and *M.* *domestica* gene/protein names are highlighted in blue and teal, respectively, while *D. melanogaster* names are in mustard. Support levels from the approximate Likelihood-Ratio Test (aLRT) from PhyML v3.0 are shown on branches. Subfamilies and individual or clustered *Drosophila* genes are indicated outside the circle to facilitate finding them in the tree. Four clades of candidate bitter receptors that are expanded in the muscids are highlighted. Pseudogenic sequences are indicated with the suffix P. **B.** Heat map of normalized expression values for *Gr* transcripts annotated from the *Stomoxys* genome.

**Fig. S12-A.**

**Fig. S12-B.**

**Figure S12. Stomoxys Ionotropic Receptor Gene Family A.** Phylogenetic tree of the *Stomoxys calcitrans* IRs with those of *Drosophila melanogaster* and *Musca domestica.* This is a maximum likelihood tree rooted by declaring the Ir8a/25a lineage as the outgroup. The *S. calcitrans* and *M.* *domestica* gene/protein names are highlighted in blue and teal, respectively, while *D. melanogaster* names are in mustard. Support levels from the approximate Likelihood-Ratio Test from PhyML v3.0 are shown on branches. Subfamilies, clades, and individual *Drosophila* genes are indicated outside the circle to facilitate finding them in the tree. Pseudogenic sequences are indicated with the suffix P. **B.** Heat map of normalized expression values for *Ir* transcripts annotated from the *Stomoxys* genome.


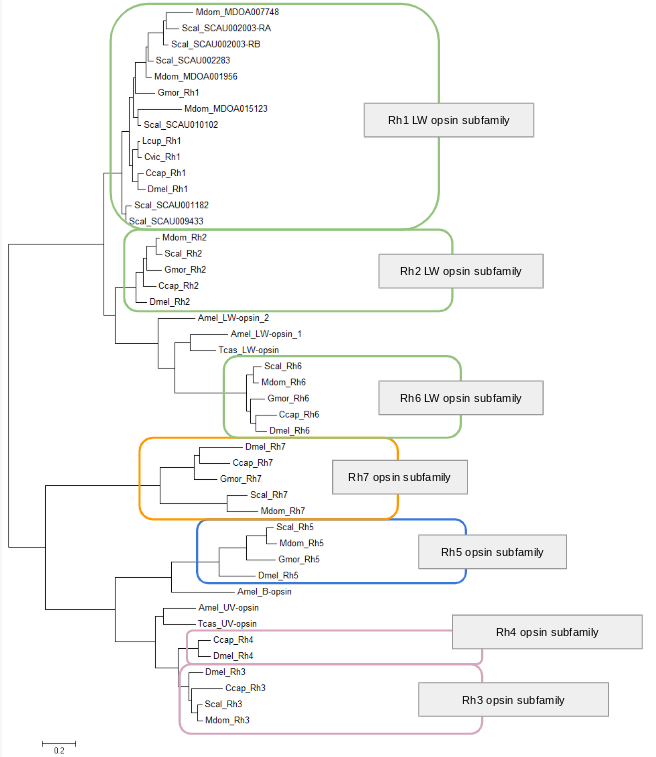


**Figure S13. Maximum likelihood tree of dipteran opsin gene relationships.** Protein sequences were aligned with Muscle [111]. Ambiguous alignment regions were filtered using Gblocks [3] using least stringent settings resulting in a final alignment with 291 sites.Bayesian tree analysis was performed out with MrBayes v3.2.6 [136] in the CIPRES Science Gateway V 3.3 environment [137], applying the GTR model of protein sequence evolution and correcting for across site substitution variation with a four rate category gamma distribution. All dipteran opsin ortholog clades were supported with credibility values higher than 0.9. Species abbreviations: Amel = *Apis mellifera*, Ccap = *Ceratitis capitata*, Cvic = *Calliphora vicina*, Dmel = *Drosophila melanogaster*, Gmor = *Glossina morsitans*, Lcup = *Lucilia cuprina*, Mdom = *Musca domestica*, Scal = *Stomoxys calcitrans*, Tcas = Tribolium castaneum. Alignment available on request.


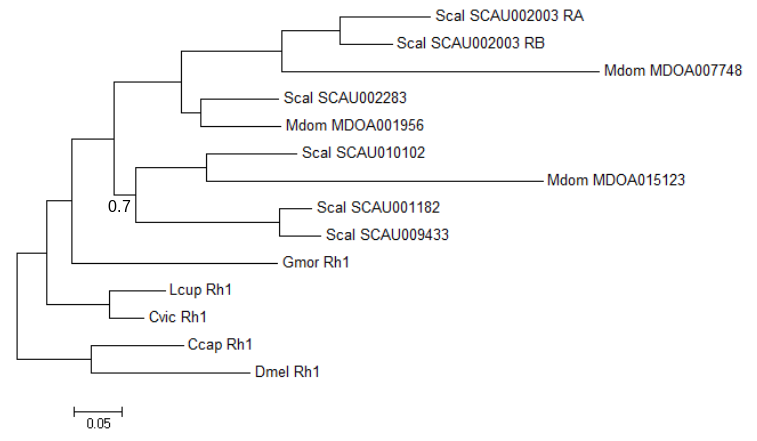


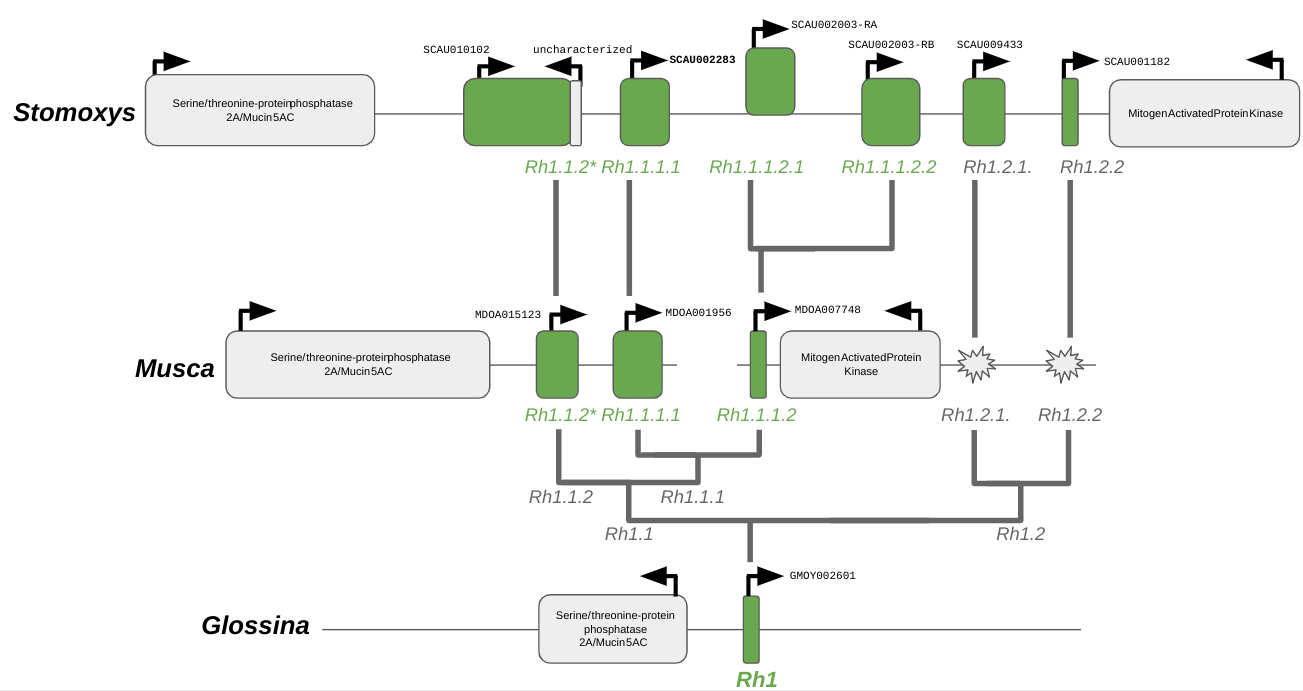


**Figure** **S14: Phylogenetic analysis and Genomic Organization of the Stomoxys Rh1 gene cluster.** Bayesian analysis of the calyptrate expansion of Rh1 opsins. Protein sequences were aligned with Webprank [138]. Ambiguous alignment regions were filtered using TrimAl (v. 1.3) [112] as implemented on the Phylemon 2.0 server [139] applying User defined settings (Minimum percentage of positions to conserve: 10, Gap threshold: 0.9, Similarity threshold: 0.0, Window size: 1.0). Bayesian gene tree estimation and species abbreviations same as for Figure S12. All branch credibility values 1 except for one internal branch with 0.7 as indicated. Alignment available on request.

Scal_Rh1.1.1.1 DPMWNKILAAYLLTIGILAWIGNGTVIYIFGTTKSLRTPANLLVINLAVS

Mdom_Rh1.1.1.1 DPIWSKILAAYLLTIGILAWIGNGTVIYIFGTTKSLRTPANLLVINLALS

Scal_Rh1.1.1.2.1 DPMWNKILAIYLVVIGILAWIGNGTVLYIFATTKSLRTPANLLVINLALS

Scal_Rh1.1.1.2.2 DPIWYKILSTYLFTIGILAWIGNGTVIYIFGTTKSLRTPANLLVINLAIS

Mdom_Rh1.1.1.2 DPIWNKILTVYLIIIGMMAWFGNGTVIYIFATTKSLRTPANLLVINLAIS

Scal_Rh1.1.2 DRMWYNILTLY**M**VLIGIISWCGNGVVIYVFSTTKSLRTPANLLVINLALS

Mdom_Rh1.1.2 DREWYNLLTLY**M**LIIGIVSWCGNGVVIFIFSSSRALRTPANLLIINLALS

Scal_Rh1.2.2 DPMWNKILMWF**M**ILIGIISWCGNGVVIYIFSTTKSLRTPANLLVINLALS

Scal_Rh1.2.1 DPMWNKILMWF**M**ILIGIISWCGNGVVIYIFSTTKSLRTPANLLVINLALS

Lcup_Rh1 DPMWAKLLTAY**M**IVIGLISWCGNGVVIYIFSTTKSLRTPANLLVINLAIS

Cvic_Rh1 EPKWAKFLAAY**M**VLIATISWCGNGVVIYIFSTTKSLRTPANLLVINLAIS

Gmor_Rh1 DPMWNKILTTY**M**IMIGCISWCGNGVVIYIFSTTKSLRTPANLLVINLALS

Dmel_Rh1 DPIWAKILTAY**M**IMIGMISWCGNGVVIYIFATTKSLRTPANLLVINLAIS

Ccap_Rh1 DPMWAKILTAY**M**ILIGTISWCGNGVVIYIFSTTKSLRTPANLLVINLALS

Dmel_Rh6 EPMWFGIIGFV**I**AILGTMSLAGNFIVMYIFTSSKGLRTPSNMFVVNLAFS

Ccap_Rh6 EQIWFHIIGFI**I**TILGVMSLSGNFIVMYIFTSTRSLRTPSNIFVVNLAFS

Gmor_Rh6 EPLWFGIIGFI**I**TVLGIMSLTGNFIVMYIFTSSKSLRTPSNMFVVNLAFS

Scal_Rh6 EPMWFGIIGFI**I**TILGIMSLAGNFVVIYIFTSAKPLRTPSNMFVVNLAFS

Mdom_Rh6 EPMWFGIIGFV**I**TVLGIMSLTGNFVVIYIFTSAKSLRTPSNMFVVNLAFS

Dmel_Rh2 DPMMSKILGLFTLAIMIISCCGNGVVVYIFGGTKSLRTPANLLVLNLAFS

Ccap_Rh2 DSTMSQILGLFTLVLLLISACGNGVVVYIFGGTKSLRTPANLLVLNLAFS

Gmor_Rh2 DTKMNQILGVFTFVIMVISLCGNGMVVFIFGSTKSLRTPANLLVLNLAFS

Mdom_Rh2 PSATSQLFGIFTAAIMVVSCCGNGVVVYIFGGTKSLRTPANLLVLNLAFS

Scal_Rh2 PSATSQLFGIFTAAIMVISCCGNGVVVYIFGGTKSLRTPANLLVLNLAFS

**Figure S15.** Analysis of tuning site 17 variation in Stomoxys and Musca Rh1 paralogs.


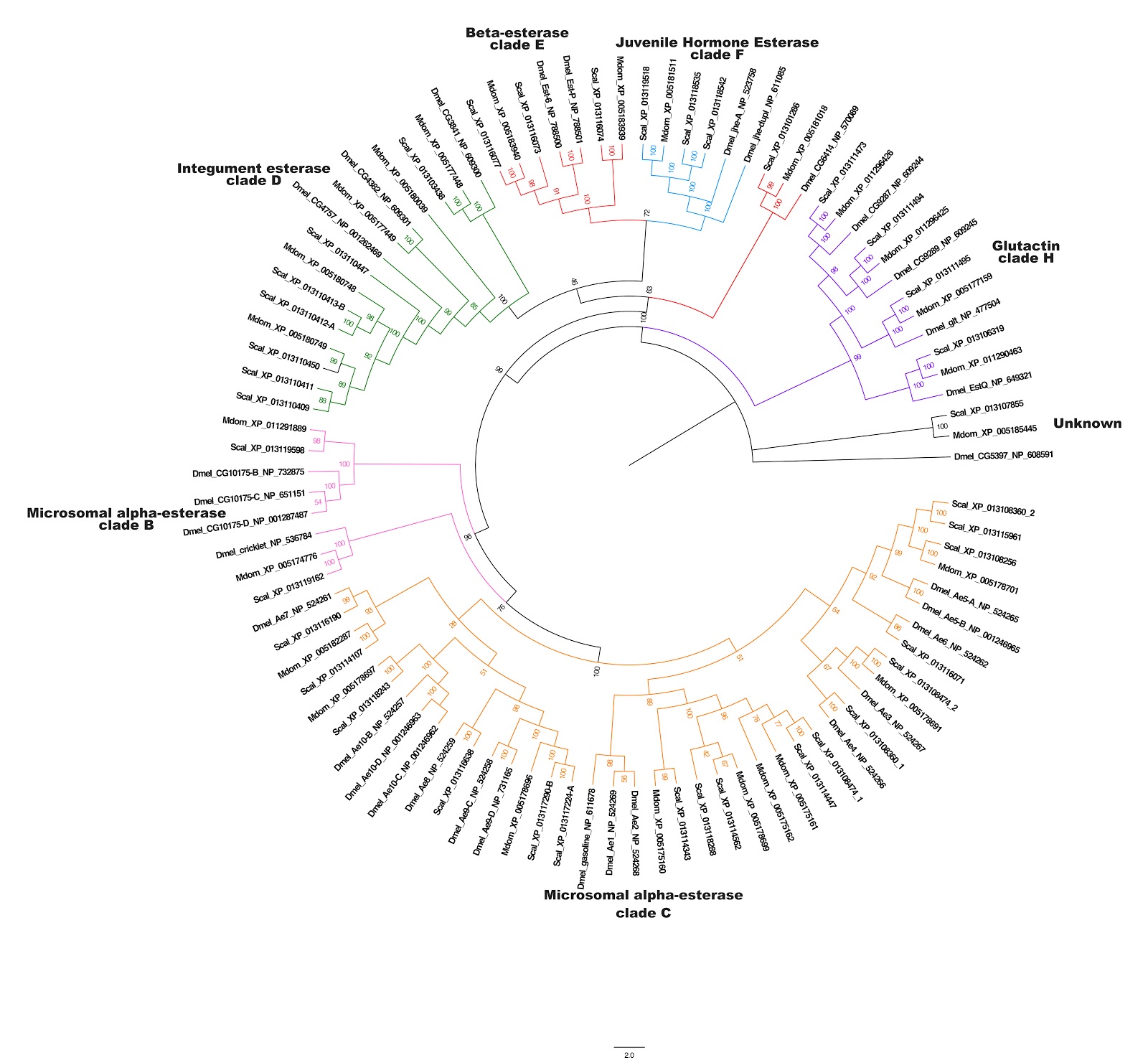


**Figure S16. Phylogenetic relationship of catalytic carboxyesterases in *Stomoxys*, *Musca*, and *Drosophila*.** Clades were designated according to nomenclature by Oakeshott et al. [140].

**Figure S17. Phylogeny of carboxylesterases with a role in neuronal development.**


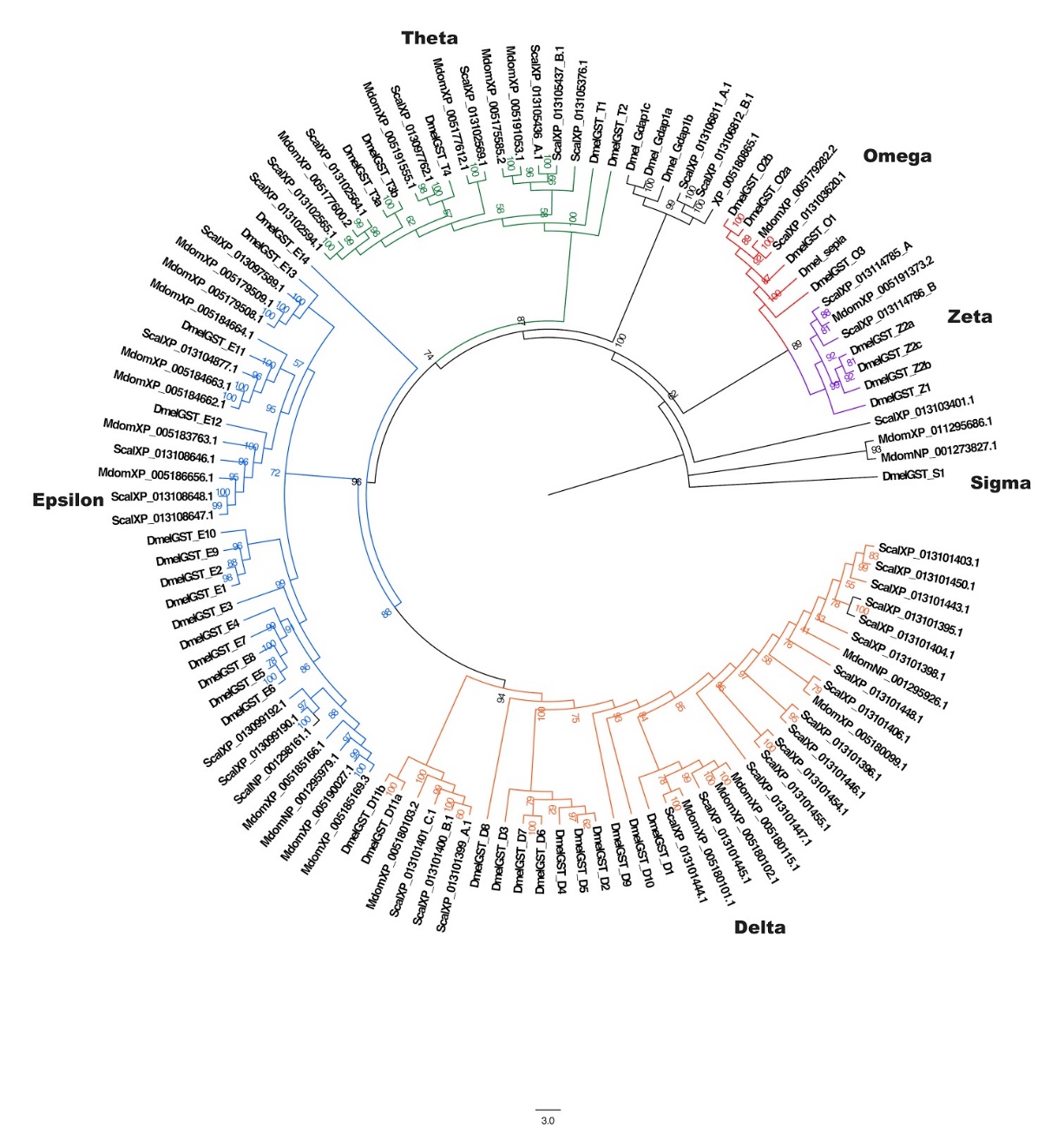


**Figure S18.** **Phylogeny of glutathione-S-transferases.** Dmel: *Drosophila melanogaster*; Mdom: *Musca domestica*.

**Figure S19.** **Phylogeny of Cys-Loop Ligand Gated Ion Channels.** Scal: *Stomoxys calcitrans,*  Dmel: *Drosophila melanogaster*; Mdom: *Musca domestica*.

**Fig. S20-A.**

**Fig. S20-B.**

**Fig. S20-C.**

**Figure S20. Cytochrome P450 (CYP) genes clustered on *Stomoxys* scaffolds and evidence for expansions in muscids relative to *Drosophila* and losses in *Drosophila*.** A. CYP9F family, B. CYP6A family, C. CYP4D family. Accession numbers for *D. melanogaster* (Dmel) and *M. domestica* (Mdom) sequences are provided on the tree labels. A. Maximum likelihood phylogeny was constructed using the web server version of IQ-TREE software ([113]; best-fit substitution model, branch support assessed with 1000 replicates of UFBoot bootstrap approximation; bootstrap percentages reported).

**
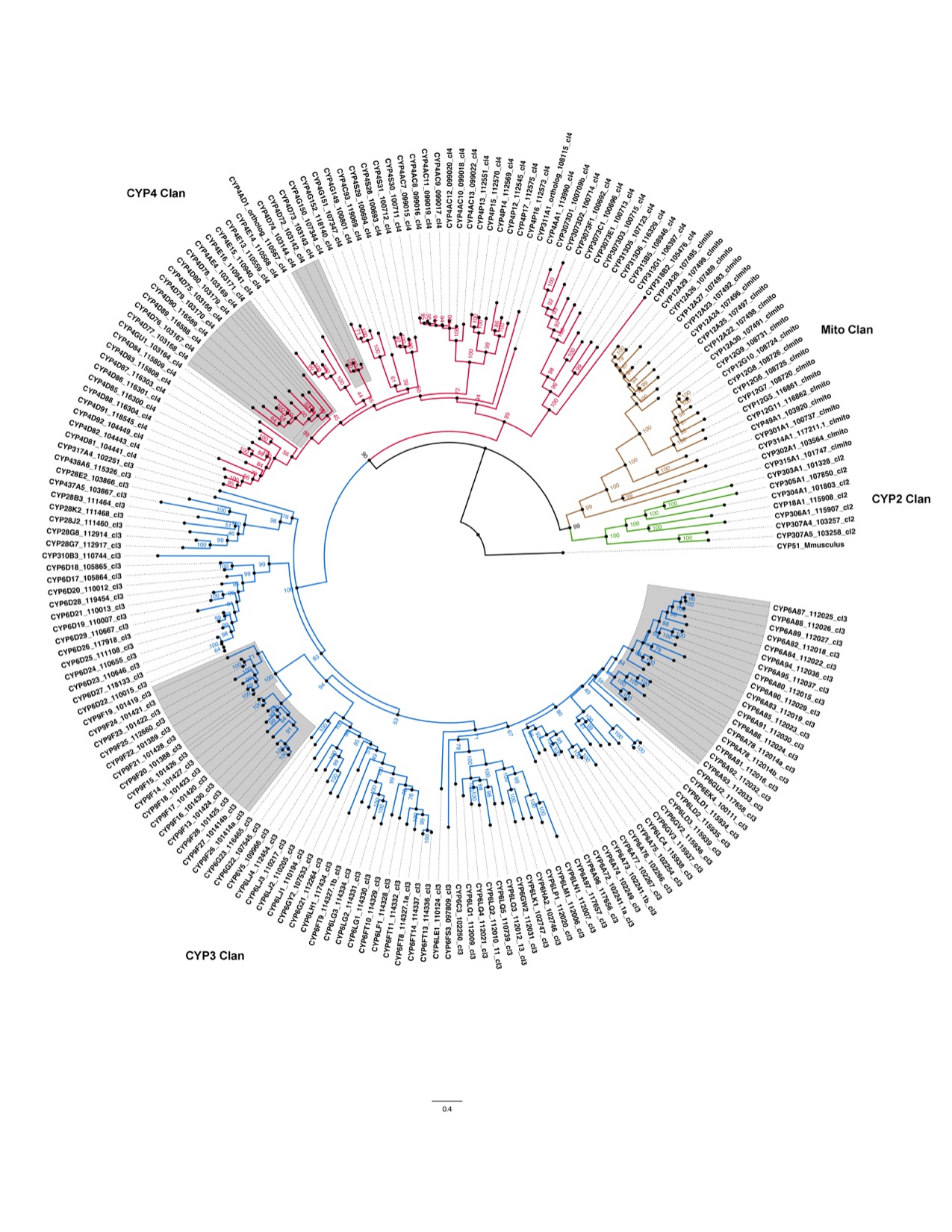
**

**Figure S21. Phylogenetic analysis of cytochrome P450 genes from *Stomoxys calcitrans***. Amino acid sequences from each family were aligned with the MUSCLE algorithm [111], and the alignments trimmed with the trimAl tool using the –strictplus option [112]. The trimmed alignment was used to construct a maximum likelihood phylogeny, rooted with *Mus musculus* CYP51 as the outgroup, with the web server version of IQ-TREE software (best-fit substitution model, branch support assessed with 1000 replicates of UFBoot bootstrap approximation; bootstrap percentages reported [113]). The CYP clades are identified by different colored lineages, and CYP gene clusters that are found in tandem within the genome are shaded in grey. P450 gene names were assigned based on comparative analyses (Additional file 13).

**Supplement References**

1. Kriventseva EV, Tegenfeldt F, Petty TJ, Waterhouse RM, Simao FA, Pozdnyakov IA, Ioannidis P, Zdobnov EM: **OrthoDB v8: update of the hierarchical catalog of orthologs and the underlying free software.** *Nucleic Acids Res* 2015, **43:**D250-256.

2. Katoh K, Misawa K, Kuma K, Miyata T: **MAFFT: a novel method for rapid multiple sequence alignment based on fast Fourier transform.** *Nucleic Acids Res* 2002, **30:**3059-3066.

3. Talavera G, Castresana J: **Improvement of phylogenies after removing divergent and ambiguously aligned blocks from protein sequence alignments.** *Syst Biol* 2007, **56:**564-577.

4. Stamatakis A: **RAxML-VI-HPC: maximum likelihood-based phylogenetic analyses with thousands of taxa and mixed models.** *Bioinformatics* 2006, **22:**2688-2690.

5. Emms DM, Kelly S: **OrthoFinder: solving fundamental biases in whole genome comparisons dramatically improves orthogroup inference accuracy.** *Genome Biol* 2015, **16:**157.

6. Foster GG, Whitten MJ, Konovalov C, Arnold JTA, Maffi G: **Autosomal Genetic Maps of the Australian Sheep Blowfly, Lucilia-Cuprina-Dorsalis R-D (Diptera, Calliphoridae), and Possible Correlations with the Linkage Maps of Musca-Domestica L and Drosophila-Melanogaster (Mg).** *Genetics Research* 1981, **37:**55-69.

7. Muller HJ: **Bearings of the ‘Drosophila’ work on systematics.** In *The New Systematics.* Edited by Huxley J. Oxford: Clarendon Press; 1940: 186-268

8. Vicoso B, Bachtrog D: **Reversal of an ancient sex chromosome to an autosome in Drosophila.** *Nature* 2013, **499:**332-335.

9. Boyes JW, Paterson HE, Corey MJ: **Somatic Chromosomes of Higher Diptera .9. Karyotypes of Some Muscid Species.** *Canadian Journal of Zoology* 1964, **42:**1025-&.

10. Joslyn DJ, Seawright JA, Willis NL: **The karyotype of the stable fly, *Stomoxys calcitrans* (L.) (Diptera: Muscidae).** *Caryologia* 1979, **32:**349-354.

11. LaChance LE: **Chromosome studies in three species of Diptera (Muscidae and Hypodermatidae).** *Annals of the Entomological Society of America* 1964, **57:**69-73.

12. Meisel RP, Olafson PU, Adhikari K, Guerrero FD, Konganti K, Benoit JB: **Sex Chromosome Evolution in Muscid Flies.** *G3 (Bethesda)* 2020, **10:**1341-1352.

13. Willis NL, Hilburn LR, Seawright JA: **Black pupa, a recessive mutant on chromosome 3 of the stable fly, *Stomoxys calcitrans* (L.).** *Journal of Heredity* 1983, **74:**114-115.

14. Walder JM, Seawright JA: **Genetic method for the separation of males and females of the house fly, *Musca domestica* (Diptera: Muscidae).** *J Econ Entomol* 1985, **78:**1030-1034.

15. Weller GL, Foster GG: **Genetic maps of the sheep blowfly *Lucilia cuprina*: linkage-group correlations with other dipteran genera.** *Genome* 1993, **36:**495-506.

16. Willis NL, Seawright JA, Nickel C, Joslyn DJ: **Reciprocal translocations and partial correlation of chromosomes in the stable fly.** *J Hered* 1981, **72:**104-106.

17. Wagoner DE: **Linkage group-karyotype correlation in the house fly determined by cytological analysis of X-ray induced translocations.** *Genetics* 1967, **57:**729-739.

18. Wagoner DE: **Linkage group-karyotype correlation in the house fly, *Musca domestica* L., confirmed by cytological analysis of x-ray induced Y-autosomal translocations.** *Genetics* 1969, **62:**115-121.

19. Seawright JA, Birky BK, Smittle BJ: **Use of a genetic technique for separating the sexes of the stable fly (Diptera: Muscidae).** *Journal of Economic Entomology* 1986, **79:**1413-1417.

20. Taskin V, Kence M: **The genetic basis of malathion resistance in housefly (*Musca domestica* L.) strains from Turkey.** *Genetika* 2004, **40:**1475-1482.

21. Meisel RP, Scott JG, Clark AG: **Transcriptome differences between alternative sex determining genotypes in the house fly, *Musca domestica*.** *Genome Biology and Evolution* 2015, **7:**2051-2061.

22. Hubley R, Finn RD, Clements J, Eddy SR, Jones TA, Bao W, Smit AF, Wheeler TJ: **The Dfam database of repetitive DNA families.** *Nucleic Acids Res* 2016, **44:**D81-89.

23. Wheeler TJ, Eddy SR: **nhmmer: DNA homology search with profile HMMs.** *Bioinformatics* 2013, **29:**2487-2489.

24. Lehane MJ, Wu D, Lehane SM: **Midgut-specific immune molecules are produced by the blood-sucking insect *Stomoxys calcitrans*.** *Proceedings of the National Academy of Sciences of the United States of America* 1997, **94:**11502-11507.

25. Boulanger N, Munks RJL, Hamilton JV, Vovelle F, Brun R, Lehane MJ, Bulet P: **Epithelial innate immunity - A novel antimicrobial peptide with antiparasitic activity in the blood-sucking insect *Stomoxys calcitrans*.** *Journal of Biological Chemistry* 2002, **277:**49921-49926.

26. Mellroth P, Karlsson J, Steiner H: **A scavenger function for a *Drosophila* peptidoglycan recognition protein.** *Journal of Biological Chemistry* 2003, **278:**7059-7064.

27. Bischoff V, Vignal C, Duvic B, Boneca IG, Hoffmann JA, Royet J: **Downregulation of the *Drosophila* immune response by peptidoglycan-recognition proteins SC1 and SC2.** *Plos Pathogens* 2006, **2:**139-147.

28. Gao YF, Tang T, Gu JH, Sun LL, Gao XB, Ma XY, Wang XC, Liu FS, Wang JH: **Downregulation of the *Musca domestica* peptidoglycan recognition protein SC (PGRP-SC) leads to overexpression of antimicrobial peptides and tardy pupation.** *Molecular Immunology* 2015, **67:**465-474.

29. Zaidman-Remy A, Herve M, Poidevin M, Pili-Floury S, Kim MS, Blanot D, Oh BH, Ueda R, Mengin-Lecreulx D, Lemaitre B: **The *Drosophila* amidase PGRP-LB modulates the immune response to bacterial infection.** *Immunity* 2006, **24:**463-473.

30. Bosco-Drayon V, Poidevin M, Boneca IG, Narbonne-Reveau K, Royet J, Charroux B: **Peptidoglycan sensing by the receptor PGRP-LE in the *Drosophila* gut induces immune responses to infectious bacteria and tolerance to microbiota.** *Cell Host & Microbe* 2012, **12:**153-165.

31. Gendrin M, Zaidman-Remy A, Broderick NA, Paredes J, Poidevin M, Roussel A, Lemaitre B: **Functional analysis of PGRP-LA in *Drosophila* immunity.** *Plos One* 2013, **8**.

32. Benton R: **Multigene family evolution: Perspectives from insect chemoreceptors.** *Trends Ecol Evol* 2015, **30:**590-600.

33. Robertson HM: **The insect chemoreceptor superfamily is ancient in animals.** *Chemical Senses* 2015, **40:**609-614.

34. Robertson HM, Warr CG, Carlson JR: **Molecular evolution of the insect chemoreceptor gene superfamily in *Drosophila melanogaster*.** *Proceedings of the National Academy of Sciences of the United States of America* 2003, **100:**14537-14542.

35. Saina M, Busengdal H, Sinigaglia C, Petrone L, Oliveri P, Rentzsch F, Benton R: **A cnidarian homologue of an insect gustatory receptor functions in developmental body patterning.** *Nature Communications* 2015, **6**.

36. Joseph RM, Carlson JR: ***Drosophila* chemoreceptors: A molecular interface between the chemical world and the brain.** *Trends in Genetics* 2015, **31:**683-695.

37. Ioannidis P, Simao FA, Waterhouse RM, Manni M, Seppey M, Robertson HM, Misof B, Niehuis O, Zdobnov EM: **Genomic features of the damselfly *Calopteryx splendens* representing a sister clade to most insect orders.** *Genome Biology and Evolution* 2017, **9:**415-430.

38. Missbach C, Dweck HKM, Vogel H, Vilcinskas A, Stensmyr MC, Hansson BS, Grosse-Wilde E: **Evolution of insect olfactory receptors.** *Elife* 2014, **3**.

39. Scott JG, Warren WC, Beukeboom LW, Bopp D, Clark AG, Giers SD, Hediger M, Jones AK, Kasai S, Leichter CA, et al: **Genome of the house fly, *Musca domestica* L., a global vector of diseases with adaptations to a septic environment.** *Genome Biology* 2014, **15**.

40. Miyamoto T, Slone J, Song XY, Amrein H: **A fructose receptor functions as a nutrient sensor in the *Drosophila* brain.** *Cell* 2012, **151:**1113-1125.

41. Fan P, Manoli DS, Ahmed OM, Chen Y, Agarwal N, Kwong S, Cai AG, Neitz J, Renslo A, Baker BS, Shah NM: **Genetic and neural mechanisms that inhibit *Drosophila* from mating with other species.** *Cell* 2013, **154:**89-102.

42. Papanicolaou A, Schetelig MF, Arensburger P, Atkinson PW, Benoit JB, Bourtzis K, Castanera P, Cavanaugh JP, Chao H, Childers C, et al: **The whole genome sequence of the Mediterranean fruit fly, *Ceratitis capitata* (Wiedemann), reveals insights into the biology and adaptive evolution of a highly invasive pest species.** *Genome Biology* 2016, **17**.

43. Kent LB, Robertson HM: **Evolution of the sugar receptors in insects.** *Bmc Evolutionary Biology* 2009, **9**.

44. Apostolopoulou AA, Mazija L, Wust A, Thum AS: **The neuronal and molecular basis of quinine-dependent bitter taste signaling in *Drosophila* larvae.** *Front Behav Neurosci* 2014, **8:**6.

45. Choi J, van Giesen L, Choi MS, Kang K, Sprecher SG, Kwon JY: **A pair of pharyngeal gustatory receptor neurons regulates caffeine-dependent ingestion in *Drosophila* larvae.** *Front Cell Neurosci* 2016, **10:**181.

46. Delventhal R, Carlson JR: **Bitter taste receptors confer diverse functions to neurons.** *Elife* 2016, **5**.

47. Kwon JY, Dahanukar A, Weiss LA, Carlson JR: **Molecular and cellular organization of the taste system in the *Drosophila* larva.** *Journal of Neuroscience* 2011, **31:**15300-15309.

48. Lee Y, Kang MJ, Shim J, Cheong CU, Moon SJ, Montell C: **Gustatory receptors required for avoiding the insecticide L-canavanine.** *Journal of Neuroscience* 2012, **32:**1429-1435.

49. Lee Y, Moon SJ, Montell C: **Multiple gustatory receptors required for the caffeine response in *Drosophila*.** *Proceedings of the National Academy of Sciences of the United States of America* 2009, **106:**4495-4500.

50. Lee Y, Moon SJ, Wang YJ, Montell C: **A *Drosophila* gustatory receptor required for strychnine sensation.** *Chemical Senses* 2015, **40:**525-533.

51. Liman ER, Zhang YV, Montell C: **Peripheral coding of taste.** *Neuron* 2014, **81:**984-1000.

52. Ling F, Dahanukar A, Weiss LA, Kwon JY, Carlson JR: **The molecular and cellular basis of taste coding in the legs of *Drosophila*.** *Journal of Neuroscience* 2014, **34:**7148-7164.

53. Montell C: **A taste of the Drosophila gustatory receptors.** *Current Opinion in Neurobiology* 2009, **19:**345-353.

54. Moon SJ, Lee Y, Jiao Y, Montell C: **A *Drosophila* gustatory receptor essential for aversive taste and inhibiting male-to-male courtship.** *Current Biology* 2009, **19:**1623-1627.

55. Park JH, Kwon JY: **A systematic analysis of *Drosophila* gustatory receptor gene expression in abdominal neurons which project to the central nervous system.** *Molecules and Cells* 2011, **32:**375-381.

56. Thorne N, Chromey C, Bray S, Amrein H: **Taste perception and coding in *Drosophila*.** *Current Biology* 2004, **14:**1065-1079.

57. Weiss LA, Dahanukar A, Kwon JY, Banerjee D, Carlson JR: **The molecular and cellular basis of bitter taste in *Drosophila*.** *Neuron* 2011, **69:**258-272.

58. Miyamoto T, Amrein H: **Suppression of male courtship by a *Drosophila* pheromone receptor.** *Nat Neurosci* 2008, **11:**874-876.

59. Wang LM, Han XQ, Mehren J, Hiroi M, Billeter JC, Miyamoto T, Amrein H, Levine JD, Anderson DJ: **Hierarchical chemosensory regulation of male-male social interactions in *Drosophila*.** *Nature Neuroscience* 2011, **14:**757-762.

60. Andrews JC, Fernández MP, Yu Q, Leary GP, Leung AK, Kavanaugh MP, Kravitz EA, Certel SJ: **Octopamine neuromodulation regulates Gr32a-linked aggression and courtship pathways in *Drosophila* males.** *PLoS Genet* 2014, **10:**e1004356.

61. Bray S, Amrein H: **A putative Drosophila pheromone receptor expressed in male-specific taste neurons is required for efficient courtship.** *Neuron* 2003, **39:**1019-1029.

62. Ejima A, Griffith LC: **Courtship initiation is stimulated by acoustic signals in *Drosophila melanogaster*.** *PLoS One* 2008, **3:**e3246.

63. Shankar S, Chua JY, Tan KJ, Calvert MEK, Weng RF, Ng WC, Mori K, Yew JY: **The neuropeptide tachykinin is essential for pheromone detection in a gustatory neural circuit.** *Elife* 2015, **4**.

64. Watanabe K, Toba G, Koganezawa M, Yamamoto D: **Gr39a, a highly diversified gustatory receptor in *Drosophila*, has a role in sexual behavior.** *Behavior Genetics* 2011, **41:**746-753.

65. Gardiner A, Barker D, Butlin RK, Jordan WC, Ritchie MG: **Evolution of a complex locus: Exon gain, loss and divergence at the Gr39a locus in *Drosophila*.** *Plos One* 2008, **3**.

66. Kim H, Jeong YT, Choi MS, Choi J, Moon SJ, Kwon JY: **Involvement of a Gr2a-expressing *Drosophila* pharyngeal gustatory receptor neuron in regulation of aversion to high-salt foods.** *Molecules and Cells* 2017, **40:**331-338.

67. Shim J, Lee Y, Jeong YT, Kim Y, Lee MG, Montell C, Moon SJ: **The full repertoire of *Drosophila* gustatory receptors for detecting an aversive compound.** *Nature Communications* 2015, **6**.

68. Thorne N, Amrein H: **Atypical expression of *Drosophila* gustatory receptor genes in sensory and central neurons.** *Journal of Comparative Neurology* 2008, **506:**548-568.

69. Barbagallo B, Garrity PA: **Temperature sensation in *Drosophila*.** *Curr Opin Neurobiol* 2015, **34:**8-13.

70. Ni LN, Bronk P, Chang EC, Lowell AM, Flam JO, Panzano VC, Theobald DL, Griffith LC, Garrity PA: **A gustatory receptor paralogue controls rapid warmth avoidance in *Drosophila*.** *Nature* 2013, **500:**580-584.

71. Xiang Y, Yuan QA, Vogt N, Looger LL, Jan LY, Jan YN: **Light-avoidance-mediating photoreceptors tile the *Drosophila* larval body wall.** *Nature* 2010, **468:**921-926.

72. Benton R, Vannice KS, Gomez-Diaz C, Vosshall LB: **Variant ionotropic glutamate receptors as chemosensory receptors in *Drosophila*.** *Cell* 2009, **136:**149-162.

73. Croset V, Rytz R, Cummins SF, Budd A, Brawand D, Kaessmann H, Gibson TJ, Benton R: **Ancient protostome origin of chemosensory ionotropic glutamate receptors and the evolution of insect taste and olfaction.** *PLoS Genet* 2010, **6:**e1001064.

74. Rytz R, Croset V, Benton R: **Ionotropic Receptors (IRs): Chemosensory ionotropic glutamate receptors in *Drosophila* and beyond.** *Insect Biochemistry and Molecular Biology* 2013, **43:**888-897.

75. Croset V, Schleyer M, Arguello JR, Gerber B, Benton R: **A molecular and neuronal basis for amino acid sensing in the *Drosophila* larva.** *Sci Rep* 2016, **6:**34871.

76. Ganguly A, Pang L, Duong VK, Lee A, Schoniger H, Varady E, Dahanukar A: **A molecular and cellular context-dependent role for Ir76b in detection of amino acid taste.** *Cell Reports* 2017, **18:**737-750.

77. Hussain A, Zhang M, Ucpunar HK, Svensson T, Quillery E, Gompel N, Ignell R, Kadow ICG: **Ionotropic chemosensory receptors mediate the taste and smell of polyamines.** *Plos Biology* 2016, **14**.

78. Zhang YLV, Ni JF, Montell C: **The molecular basis for attractive salt-taste coding in *Drosophila*.** *Science* 2013, **340:**1334-1338.

79. Ai M, Blais S, Park JY, Min S, Neubert TA, Suh GS: **Ionotropic glutamate receptors IR64a and IR8a form a functional odorant receptor complex in vivo in *Drosophila*.** *J Neurosci* 2013, **33:**10741-10749.

80. Ai M, Min S, Grosjean Y, Leblanc C, Bell R, Benton R, Suh GS: **Acid sensing by the *Drosophila* olfactory system.** *Nature* 2010, **468:**691-695.

81. Grosjean Y, Rytz R, Farine JP, Abuin L, Cortot J, Jefferis GSXE, Benton R: **An olfactory receptor for food-derived odours promotes male courtship in *Drosophila*.** *Nature* 2011, **478:**236-U123.

82. Prieto-Godino LL, Rytz R, Bargeton B, Abuin L, Arguello JR, Dal Peraro M, Benton R: **Olfactory receptor pseudo-pseudogenes.** *Nature* 2016, **539:**93-97.

83. Prieto-Godino LL, Rytz R, Cruchet S, Bargeton B, Abuin L, Silbering AF, Ruta V, Dal Peraro M, Benton R: **Evolution of acid-sensing olfactory circuits in Drosophilids.** *Neuron* 2017, **93:**661-667.

84. Enjin A, Zaharieva EE, Frank DD, Mansourian S, Suh GS, Gallio M, Stensmyr MC: **Humidity sensing in *Drosophila*.** *Curr Biol* 2016, **26:**1352-1358.

85. Knecht ZA, Silbering AF, Cruz J, Yang L, Croset V, Benton R, Garrity PA: **lonotropic receptor-dependent moist and dry cells control hygrosensation in *Drosophila*.** *Elife* 2017, **6**.

86. Knecht ZA, Silbering AF, Ni LN, Klein M, Budelli G, Bell R, Abuin L, Ferrer AJ, Samuel ADT, Benton R, Garrity PA: **Distinct combinations of variant ionotropic glutamate receptors mediate thermosensation and hygrosensation in *Drosophila*.** *Elife* 2016, **5**.

87. Ni L, Klein M, Svec KV, Budelli G, Chang EC, Ferrer AJ, Benton R, Samuel ADT, Garrity PA: **The ionotropic receptors IR21a and IR25a mediate cool sensing in *Drosophila*.** *Elife* 2016, **5**.

88. Koh TW, He Z, Gorur-Shandilya S, Menuz K, Larter NK, Stewart S, Carlson JR: **The *Drosophila* IR20a clade of ionotropic receptors are candidate taste and pheromone receptors.** *Neuron* 2014, **83:**850-865.

89. Stewart S, Koh TW, Ghosh AC, Carlson JR: **Candidate ionotropic taste receptors in the *Drosophila* larva.** *Proceedings of the National Academy of Sciences of the United States of America* 2015, **112:**4195-4201.

90. Joseph RM, Sun JS, Tam E, Carlson JR: **A receptor and neuron that activate a circuit limiting sucrose consumption.** *Elife* 2017, **6:**e24992.

91. Sanchez-Alcaniz JA, Silbering AF, Croset V, Zappia G, Sivasubramaniam AK, Abuin L, Sahai SY, Munch D, Steck K, Auer TO, et al: **An expression atlas of variant ionotropic glutamate receptors identifies a molecular basis of carbonation sensing.** *Nat Commun* 2018, **9:**4252.

92. Leader DP, Krause SA, Pandit A, Davies SA, Dow JA: **FlyAtlas 2: a new version of the Drosophila melanogaster expression atlas with RNA-Seq, miRNA-Seq and sex-specific data.** *Nucleic acids research* 2017.

93. Sánchez-Gracia A, Rozas J: **Divergent evolution and molecular adaptation in the *Drosophila* odorant-binding protein family: inferences from sequence variation at the OS-E and OS-Fgenes.** *BMC Evolutionary Biology* 2008, **8:**323.

94. Olafson PU: **Molecular characterization and immunolocalization of the olfactory co-receptor Orco from two blood-feeding muscid flies, the stable fly (*Stomoxys calcitrans*, L.) and the horn fly (*Haematobia irritans irritans*, L.).** *Insect Mol Biol* 2013, **22:**131-142.

95. Kim DH, Kim SI, Chang KS, Ahn YJ: **Repellent activity of constituents identified in *Foeniculum vulgare* fruit against *Aedes aegypti* (Diptera: Culicidae).** *J Agric Food Chem* 2002, **50:**6993-6996.

96. Hallem EA, Ho MG, Carlson JR: **The molecular basis of odor coding in the *Drosophila* antenna.** *Cell* 2004, **117:**965-979.

97. Termtanasombat M, Mitsuno H, Misawa N, Yamahira S, Sakurai T, Yamaguchi S, Nagamune T, Kanzaki R: **Cell-based odorant sensor array for odor discrimination based on insect odorant receptors.** *J Chem Ecol* 2016, **42:**716-724.

98. Hieu TT, Jung J, Kim SI, Ahn YJ, Kwon HW: **Behavioural and electroantennogram responses of the stable fly (*Stomoxys calcitrans* L.) to plant essential oils and their mixtures with attractants.** *Pest Manag Sci* 2014, **70:**163-172.

99. Birkett MA, Agelopoulos N, Jensen KM, Jespersen JB, Pickett JA, Prijs HJ, Thomas G, Trapman JJ, Wadhams LJ, Woodcock CM: **The role of volatile semiochemicals in mediating host location and selection by nuisance and disease-transmitting cattle flies.** *Med Vet Entomol* 2004, **18:**313-322.

100. Ray A, van Naters W, Shiraiwa T, Carlson JR: **Mechanisms of odor receptor gene choice in *Drosophila*.** *Neuron* 2007, **53:**353-369.

101. Hallem EA, Carlson JR: **Coding of odors by a receptor repertoire.** *Cell* 2006, **125:**143-160.

102. Jeanbourquin P, Guerin PM: **Chemostimuli implicated in selection of oviposition substrates by the stable fly *Stomoxys calcitrans*.** *Medical and Veterinary Entomology* 2007, **21:**209-216.

103. Jeanbourquin P, Guerin PM: **Sensory and behavioural responses of the stable fly *Stomoxys calcitrans* to rumen volatiles.** *Medical and Veterinary Entomology* 2007, **21:**217-224.

104. Grillet M, Dartevelle L, Ferveur JF: **A *Drosophila* male pheromone affects female sexual receptivity.** *Proc Biol Sci* 2006, **273:**315-323.

105. Marshall B, Warr CG, de Bruyne M: **Detection of volatile indicators of illicit substances by the olfactory receptors of *Drosophila melanogaster*.** *Chem Senses* 2010, **35:**613-625.

106. Tangtrakulwanich K, Chen H, Baxendale F, Brewer G, Zhu JJ: **Characterization of olfactory sensilla of *Stomoxys calcitrans* and electrophysiological responses to odorant compounds associated with hosts and oviposition media.** *Med Vet Entomol* 2011, **25:**327-336.

107. Stocker RF: **Design of the larval chemosensory system.** In *Brain Development in Drosophila melanogaster.* Edited by Technau GM. New York, NY: Springer New York; 2008: 69-81

108. Ebrahim SA, Dweck HK, Stokl J, Hofferberth JE, Trona F, Weniger K, Rybak J, Seki Y, Stensmyr MC, Sachse S, et al: ***Drosophila* avoids parasitoids by sensing their semiochemicals via a dedicated olfactory circuit.** *PLoS Biol* 2015, **13:**e1002318.

109. Wanner KW, Willis LG, Theilmann DA, Isman MB, Feng Q, Plettner E: **Analysis of the insect OS-D-like gene family.** *J Chem Ecol* 2004, **30:**889-911.

110. Gong DP, Zhang HJ, Zhao P, Lin Y, Xia QY, Xiang ZH: **Identification and expression pattern of the chemosensory protein gene family in the silkworm, *Bombyx mori*.** *Insect Biochem Mol Biol* 2007, **37:**266-277.

111. Edgar RC: **MUSCLE: multiple sequence alignment with high accuracy and high throughput.** *Nucleic Acids Research* 2004, **32:**1792-1797.

112. Capella-Gutierrez S, Silla-Martinez JM, Gabaldon T: **trimAl: a tool for automated alignment trimming in large-scale phylogenetic analyses.** *Bioinformatics* 2009, **25:**1972-1973.

113. Trifinopoulos J, Nguyen LT, von Haeseler A, Minh BQ: **W-IQ-TREE: a fast online phylogenetic tool for maximum likelihood analysis.** *Nucleic Acids Res* 2016, **44:**W232-235.

114. Sogorb MA, Vilanova E: **Enzymes involved in the detoxification of organophosphorus, carbamate and pyrethroid insecticides through hydrolysis.** *Toxicology Letters* 2002, **128:**215-228.

115. Li XC, Schuler MA, Berenbaum MR: **Molecular mechanisms of metabolic resistance to synthetic and natural xenobiotics.** *Annual Review of Entomology* 2007, **52:**231-253.

116. Ranson H, Claudianos C, Ortelli F, Abgrall C, Hemingway J, Sharakhova MV, Unger MF, Collins FH, Feyereisen R: **Evolution of supergene families associated with insecticide resistance.** *Science* 2002, **298:**179-181.

117. Temeyer KB, Chen AC: **Acetylcholinesterase of *Stomoxys calcitrans* (L.) (Diptera: Muscidae): cDNA sequence, baculovirus expression, and biochemical properties.** *Veterinary Parasitology* 2012, **184:**92-95.

118. Ffrench-Constant RH, Daborn PJ, Le Goff G: **The genetics and genomics of insecticide resistance.** *Trends Genet* 2004, **20:**163-170.

119. Ding Y, Ortelli F, Rossiter LC, Hemingway J, Ranson H: **The *Anopheles gambiae* glutathione transferase supergene family: annotation, phylogeny and expression profiles.** *BMC Genomics* 2003, **4:**35.

120. Strode C, Wondji CS, David JP, Hawkes NJ, Lumjuan N, Nelson DR, Drane DR, Karunaratne SH, Hemingway J, Black WCt, Ranson H: **Genomic analysis of detoxification genes in the mosquito *Aedes aegypti*.** *Insect Biochem Mol Biol* 2008, **38:**113-123.

121. Tu CP, Akgul B: ***Drosophila* glutathione S-transferases.** *Methods Enzymol* 2005, **401:**204-226.

122. Enayati AA, Ranson H, Hemingway J: **Insect glutathione transferases and insecticide resistance.** *Insect Molecular Biology* 2005, **14:**3-8.

123. Bloomquist JR: **Chloride channels as tools for developing selective insecticides.** *Arch Insect Biochem Physiol* 2003, **54:**145-156.

124. Ffrench-Constant RH, Williamson MS, Davies TG, Bass C: **Ion channels as insecticide targets.** *J Neurogenet* 2016, **30:**163-177.

125. Grutter T, Changeux JP: **Nicotinic receptors in wonderland.** *Trends Biochem Sci* 2001, **26:**459-463.

126. Jones AK, Grauso M, Sattelle DB: **The nicotinic acetylcholine receptor gene family of the malaria mosquito, *Anopheles gambiae*.** *Genomics* 2005, **85:**176-187.

127. Jones AK, Raymond-Delpech V, Thany SH, Gauthier M, Sattelle DB: **The nicotinic acetylcholine receptor gene family of the honey bee, *Apis mellifera*.** *Genome Res* 2006, **16:**1422-1430.

128. Jones AK, Sattelle DB: **The cys-loop ligand-gated ion channel gene superfamily of the red flour beetle, *Tribolium castaneum*.** *BMC Genomics* 2007, **8:**327.

129. Sattelle DB, Jones AK, Sattelle BM, Matsuda K, Reenan R, Biggin PC: **Edit, cut and paste in the nicotinic acetylcholine receptor gene family of *Drosophila melanogaster*.** *Bioessays* 2005, **27:**366-376.

130. Jones AK, Bera AN, Lees K, Sattelle DB: **The cys-loop ligand-gated ion channel gene superfamily of the parasitoid wasp, *Nasonia vitripennis*.** *Heredity (Edinb)* 2010, **104:**247-259.

131. International Glossina Genome Initiative: **Genome sequence of the tsetse fly (*Glossina morsitans*): Vector of African trypanosomiasis.** *Science* 2014, **344:**380-386.

132. Anstead CA, Korhonen PK, Young ND, Hall RS, Jex AR, Murali SC, Hughes DST, Lee SF, Perry T, Stroehlein AJ, et al: ***Lucilia cuprina* genome unlocks parasitic fly biology to underpin future interventions.** *Nature Communications* 2015, **6**.

133. Benoit JB, Hansen IA, Attardo GM, Michalkova V, Mireji PO, Bargul JL, Drake LL, Masiga DK, Aksoy S: **Aquaporins are critical for provision of water during lactation and intrauterine progeny hydration to maintain tsetse fly reproductive success.** *Plos Neglected Tropical Diseases* 2014, **8**.

134. Simao FA, Waterhouse RM, Ioannidis P, Kriventseva EV, Zdobnov EM: **BUSCO: assessing genome assembly and annotation completeness with single-copy orthologs.** *Bioinformatics* 2015, **31:**3210-3212.

135. Chang CI, Chelliah Y, Borek D, Mengin-Lecreulx D, Deisenhofer J: **Structure of tracheal cytotoxin in complex with a heterodimeric pattern-recognition receptor.** *Science* 2006, **311:**1761-1764.

136. Ronquist F, Huelsenbeck JP: **MrBayes 3: Bayesian phylogenetic inference under mixed models.** *Bioinformatics* 2003, **19:**1572-1574.

137. Miller MA, Schwartz T, Pickett BE, He S, Klem EB, Scheuermann RH, Passarotti M, Kaufman S, O'Leary MA: **A RESTful API for Access to Phylogenetic Tools via the CIPRES Science Gateway.** *Evolutionary Bioinformatics* 2015, **11:**43-48.

138. Loytynoja A, Goldman N: **webPRANK: a phylogeny-aware multiple sequence aligner with interactive alignment browser.** *BMC Bioinformatics* 2010, **11:**579.

139. Sanchez R, Serra F, Tarraga J, Medina I, Carbonell J, Pulido L, de Maria A, Capella-Gutierrez S, Huerta-Cepas J, Gabaldon T, et al: **Phylemon 2.0: a suite of web-tools for molecular evolution, phylogenetics, phylogenomics and hypotheses testing.** *Nucleic Acids Res* 2011, **39:**W470-474.

140. Oakeshott JG, Claudianos C, Campbell PM, Newcomb RD, Russell RJ: **Biochemical genetics and genomics of insect esterases.** In *Insect Pharmacology: Channels, Receptors, toxins and enzymes.* Edited by Gilbert LI, Gill SS. London UK: Elsevier; 2005: 229-301
